# The 2.1 Å structure of protein F9 and its comparison to L1, two components of the conserved poxvirus entry-fusion complex

**DOI:** 10.1038/s41598-018-34244-7

**Published:** 2018-11-14

**Authors:** Ulrike S. Diesterbeck, Apostolos G. Gittis, David N. Garboczi, Bernard Moss

**Affiliations:** 1 Laboratory of Viral Diseases, National Institute of Allergy and Infectious Diseases, National Institutes of Health, Bethesda, Maryland USA; 2 Structural Biology Section, Research Technologies Branch, National Institute of Allergy and Infectious Diseases, National Institutes of Health, Rockville, Maryland USA

## Abstract

The poxvirus F9 protein is a component of the vaccinia virus entry fusion complex (EFC) which consists of 11 proteins. The EFC forms a unique apparatus among viral fusion proteins and complexes. We solved the atomic structure of the F9 ectodomain at 2.10 Å. A structural comparison to the ectodomain of the EFC protein L1 indicated a similar fold and organization, in which a bundle of five α-helices is packed against two pairs of β-strands. However, instead of the L1 myristoylation site and hydrophobic cavity, F9 possesses a protruding loop between α-helices α3 and α4 starting at Gly90. Gly90 is conserved in all poxviruses except Salmon gill poxvirus (SGPV) and *Diachasmimorpha longicaudata* entomopoxvirus. Phylogenetic sequence analysis of all *Poxviridae* F9 and L1 orthologs revealed the SGPV genome to contain the most distantly related F9 and L1 sequences compared to the vaccinia proteins studied here. The structural differences between F9 and L1 suggest functional adaptations during evolution from a common precursor that underlie the present requirement for each protein.

## Introduction

Poxviruses are large enveloped DNA viruses that replicate in the cytoplasm and exist in three distinctive infectious forms: mature virions (MV), wrapped virions (WV), and extracellular virions (EV)^1^. The MV consists of a nucleoprotein core surrounded by a single lipoprotein membrane thought to be derived from the endoplasmic reticulum; the WV contains two additional membranes acquired from the trans-Golgi network; during exocytosis the outer WV membrane is lost forming the EV, which has two membranes^2–4^. To enable replication, the viral core harboring the double-stranded DNA genome is released into the cytoplasm, which defines the entry step^5^. MVs can enter the cell at neutral pH at the plasma membrane or through an endocytic route at low pH^6,7^. EVs shed their outer membrane at the cell surface allowing MV-like particles to fuse with the plasma or endosomal membrane^8^ following macropinocytosis^9^. The fusion apparatus is embedded in the MV membrane and consists of eleven proteins namely A16^10^, A21^11^, A28^12^, F9^13^, G3^14^, G9^15^, H2^16^, J5^17^, L1^18^, L5^19^, and O3^20^, which comprise the entry fusion complex (EFC). These proteins were identified in vaccinia virus (VACV) based on the inability of mutants to mediate entry and their interaction to form a complex. All *Poxviridae* share orthologs of these proteins with the apparent exception of Salmon gill poxvirus (SGPV), for which homologs of G3, L5, and O3 were not identified^21^. Three heterodimeric sub-complexes, A16/G9, A28/H2, and G3/L5, have been identified in the VACV EFC^22–24^. Interactions between these and other components of the EFC remain under investigation. The EFC proteins have N- or C-terminal hydrophobic domains but lack signal peptides and are unglycosylated, suggesting that they do not normally traffic through the ER and likely insert directly into the viral membrane. The EFC proteins are not required for the attachment of virus particles to cells, which is mediated by other viral proteins that interact with glycosaminoglycans and chondroitin sulfate^25–27^.

The association of the 11 EFC proteins was demonstrated by immunoaffinity purification^17^, but the complex has not been physically characterized. Since each EFC component has a putative transmembrane domain, they may be organized in a two-dimensional network on the MV membrane. Except for L1 and F9, each of the EFC proteins is required for assembly or stability of the complex. For this reason, L1 and F9 are sometimes referred to as EFC-associated proteins and the others as core components^13,18^. L1 and F9 have approximately 20% amino acid identity. Nevertheless, each is required for entry suggesting that they arose from a common ancestral gene but evolved distinct functions^13^. Knock-down of F9 has a greater effect on the hemifusion step of entry than knock-down of L1^28^. In addition, the binding of a neutralizing antibody to a conformational epitope on L1^29,30^ reduces core entry but not the hemifusion step^28^. Polyclonal antibodies induced by immunizing rabbits with soluble F9 protein neutralize infectivity but were not tested in a hemifusion assay^13^. Recently, a human monoclonal antibody against F9 was isolated and characterized but did not neutralize virus in a plaque reduction test^31^.

Based on their primary structures, none of the EFC proteins resemble type 1, 2 or 3 entry proteins of other viruses. Until now, L1 was the only EFC component with a determined atomic structure^32^. The L1 structure exhibits a bundle of α-helices leaning against a pair of two-stranded β-sheets and a disorganized C-terminal tail that is proximal to the transmembrane domain. A hydrophobic cavity capable of accepting and shielding a conjugated myristic acid is located near the N-terminus^32^. Myristoylation of L1 is required for infectivity but not for assembly of the EFC and incorporation into viral particles^33^. The amino acid motif for adding a myristate moiety is conserved in all poxvirus orthologs of L1, except in Salmon gill poxvirus (SGPV)^21^. In contrast, none of the F9 orthologs have a predicted myristoylation site.

F9, the subject of this study, is a 23.8 kDa protein with a putative transmembrane domain near the C-terminus. Based on the amino acid sequence, F9 was predicted to have a structure similar to that of L1^32^. Here, we report the 2.10 Å structure of F9 and demonstrate a fold similar to L1 but without a hydrophobic cavity. A novel feature, not found in L1, is a loop protruding from the F9 surface. We also analyzed the phylogeny of F9 and L1, which likely arose from a common ancestral gene, within the family *Poxviridae*. Homology based three-dimensional models of SGPV F9 and L1, the most distant orthologs relative to VACV, were generated and compared to the atomic structures of VACV F9 and L1.

## Results

### The structure and topology of the poxvirus F9 protein

**Figure 1:**
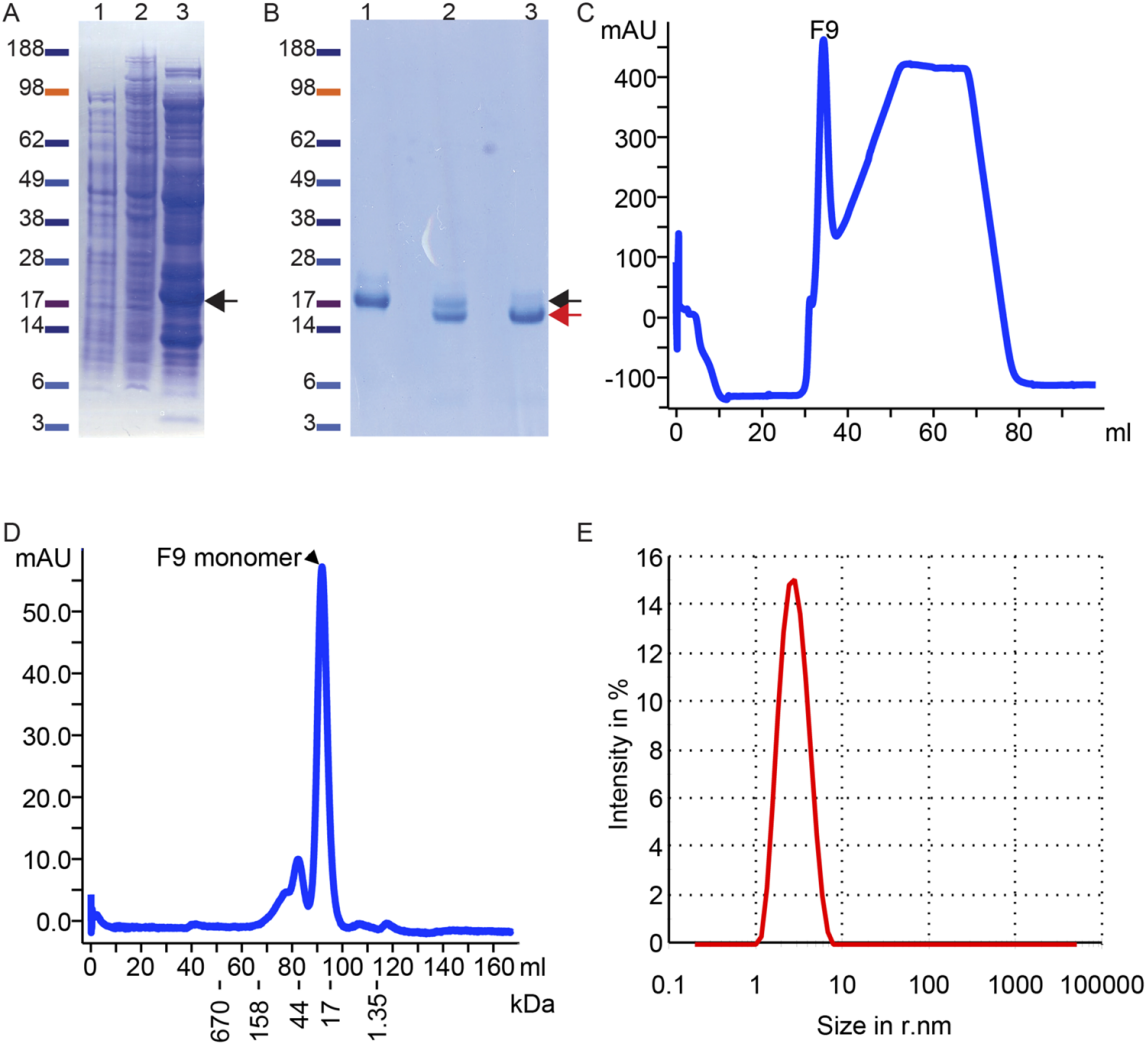
Expression and purification of F9. (**A, B**) Coomassie-stained SDS-polyacrylamide gels. (**A**) Non-induced (lane 1), IPTG induced (lane 2) *E. coli* cell lysates, and isolated inclusion bodies (lane 3). (**B**) Purification after refolding occurred first via immobilized metal affinity chromatography (IMAC) using Ni-NTA and the 6xHis tag fused N-terminal to F9 (lane 1); after cleavage of the tag (lane 2) the F9 was again purified via IMAC and the flow-through with mostly tag-free F9 was collected (lane 3). In (**A, B**) the black arrow marks the non-cleaved F9 whereas the red arrow labels the F9 without the 6xHis tag. (**C**) Protein (F9) was detected by OD_280_ during the first IMAC step. The second peak with plateau corresponds to increasing concentrations of imidazole. (**D**) The concentrated and cleaved F9 was subjected to size exclusion chromatography (SEC). (**E**) After concentrating the SEC purified F9 to 10 mg/ml, the integrity was measured by dynamic light scattering. Abbreviations: mAU, milli-absorbance units at OD_280nm_, r.nm, radius in nm. Full-sized Coomassie gels are shown in Supplemental Fig. 1. Gels were scanned and were not modified otherwise.

The VACV strain Western Reserve F9 ectodomain, consisting of residues 1–176 with an added N-terminal 6-histidine tag, was refolded from bacterially-produced inclusion bodies (Fig. 1A) and purified to near homogeneity by immobilized metal affinity chromatography (IMAC) and size exclusion chromatography (SEC) (Fig. 1B–D). The integrity of the recombinant F9 was studied with dynamic light scattering. Its polydispersity index of 0.14 demonstrated a uniform protein sample. At an F9 concentration of 10 mg/ml, the measured hydrodynamic radius^34^ of 2.93 nm suggested that F9 may not have been monomeric at high concentration (Fig. 1E). Using the 10 mg/ml F9 solution, large octahedral crystals were obtained in hanging drop vapor diffusion experiments.

**Figure 2:**
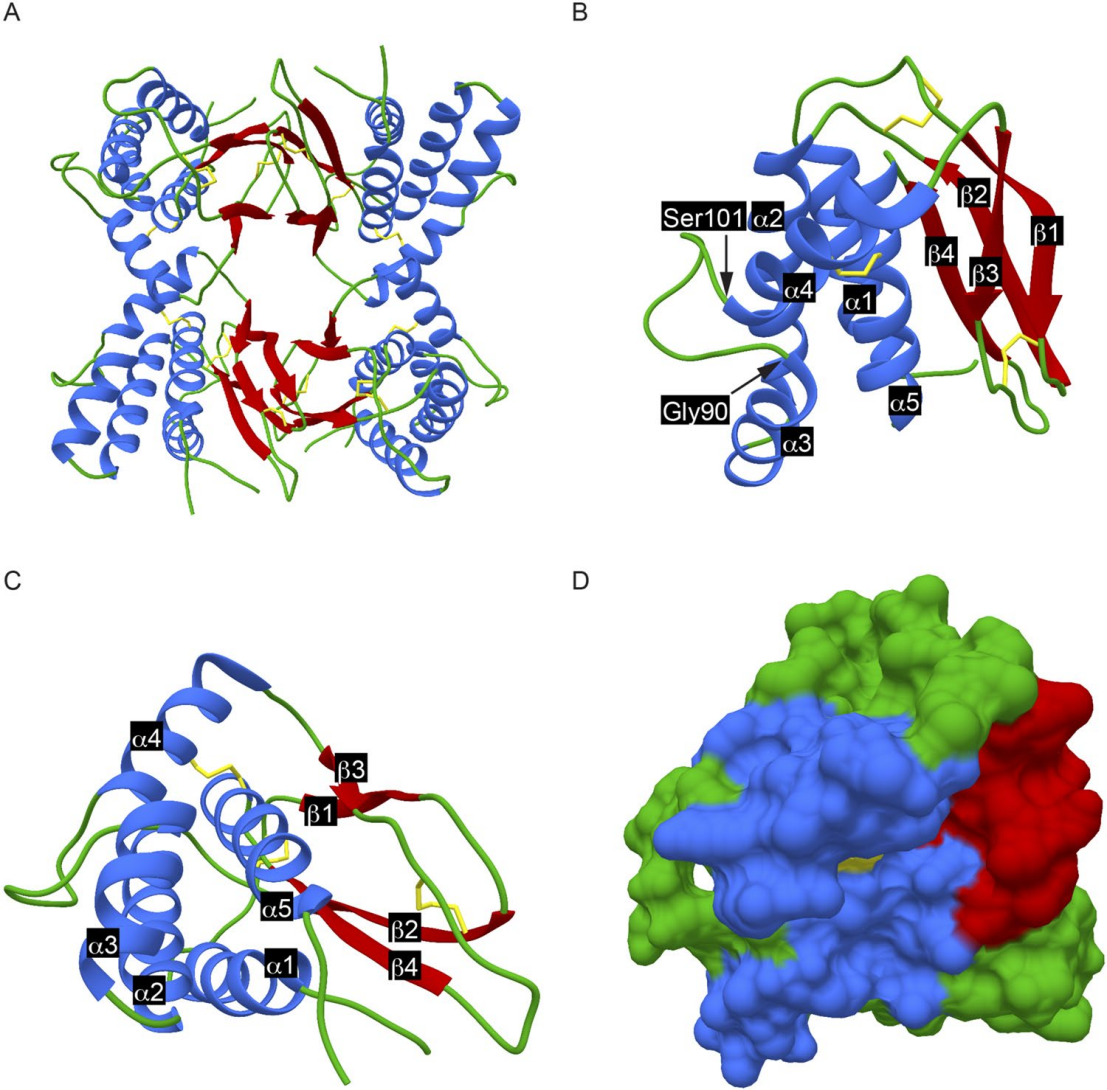
Asymmetric unit and three-dimensional structure of F9: (**A–C**) Structural representation of F9 as ribbon diagrams and (**D**) surface. Blue represents α-helices, red marks β-strands, green is connecting loops, and yellow denotes disulfide bonds. (**A**) F9 crystallized with four molecules in the asymmetric unit of the native crystal. A large contact site (1478 Å^2^ total buried area) was found between the β-sheets and smaller contact sites between the α-helices of neighboring molecules in the crystal. (**B, D**) Side projection of ribbon diagram (**B**) and surface (**D**) within the F9 molecule, a bundle of five α-helices (α1-α5) is packed against two pairs of β-sheets (β1-β4). Between Gly90 and Ser101 a loop protrudes from the surface. (**C**) The 90° x-axis turned bottom view shows the position of α5, β3, and β4 in near the center of the molecule.

The structure of F9 was determined to 2.10 Å resolution by X-ray crystallography using phase estimates calculated from X-ray data collected from sodium iodide-soaked F9 crystals (Table 1). Molecular replacement calculations using the L1 structural model had been unsuccessful, even though the 27% sequence identity between F9 and L1 ectodomains predicted a similar protein fold. The structure of F9, crystallized with two molecules in the asymmetric unit in the sodium-iodide crystals, was solved to 3.20 Å maximum resolution. F9 crystallized with four molecules in the asymmetric unit of the crystal used for data collection for refinement, resulting in the 2.10 Å structure (Fig. 2A). The F9 ectodomain exhibits a bundle of five α-helices (α) packed against two pairs of β-sheets (β). One pair of β-sheets consists of β1 and β3, whereas the other pair comprises β2 and β4. A short loop of four amino acid residues connects β1 and β2. The linker between β3 and β4 is an 11-amino acid polypeptide at the bottom of the molecule. Connections from and to the α-helices occur on the top of the F9 molecule. Between α3 and α4, a loop of 12 amino acids protrudes starting with Gly90 and ending with Ser101 (Fig. 2B, C). At the bottom of the F9 ectodomain, the N-terminal end of the α1 helix is located near to the end of the α5 helix. As the transmembrane domain begins after the α5 helix, both ends of F9 are close to each other and to the viral membrane. The strands α5, β3, and β4 are positioned in the middle as visible from the bottom of the molecule (Fig. 2C).

**Table 1:** F9 data collection and refinement statistics.

Data for experimental phases	F9-NaI
Space group	P4_1_22
Cell dimensions (a, b, c in Å)	82.73, 82.73, 143.14
Mosaicity (°)	0.72
Resolution range (Å)	29.25–3.20 (3.31–3.20)^a^
Total No of reflections	159117
No of possible unique reflections	8710 (857)
No of unique reflections	8707 (854)
I /σ(I) unaveraged	5.20 (2.30)
I /σ(I) averaged	20.20 (7.90)
Completeness (%)	99.90 (99.6)
Redundancy	18.27 (17.83)
R-merge^b^	0.095 (0.354)
R-means^c^	0.097 (0.365)
**Anomalous signal**	
DeltaA^d^ acentric	3.61
DeltaC^d^ centric	1.89
R(ano)^d^	1.91
**Data for refinement**	F9-native
Space Group	P2_1_2_1_2_1_
Cell dimensions (a, b, c in Å)	65.45, 75.08, 136.25
Resolution (Å)	30.00–2.10
No. of reflections	37485
R_work_/R_free_	22.57/26.85
**No. of atoms**	
Protein	4947
Chain A	1239
Chain B	1211
Chain C	1226
Chain D	1271
Ligand	898
Water	217
**Refinement**	
B-factors (Å^2^)	
Protein	39.33
Chain A	41.09
Chain B	40.87
Chain C	36.14
Chain D	39.22
Ligand	57.50
Water	43.97
**Root mean square deviations**	
Bond lengths (Å)	0.014
Bond angles (°)	1.728

^a^Values in parentheses are for highest-resolution shell.

^b^$${{\rm{R}}}_{{\rm{merge}}}({\rm{I}})$$ = $${\sum }_{{\rm{hkl}}}\,{\sum }_{{\rm{i}}=1}\,|{{\rm{I}}}_{{\rm{i}}}({\rm{hkl}})-\langle {\rm{I}}({\rm{hkl}})\rangle |$$/$${\sum }_{{\rm{hkl}}}\,{\sum }_{{\rm{i}}}{{\rm{I}}}_{{\rm{i}}}-({\rm{hkl}})$$. ^c^$${{\rm{R}}}_{{\rm{means}}}({\rm{I}})$$ = $${\sum }_{{\rm{hkl}}}\,{\sum }_{{\rm{i}}=1}\,\{\sqrt{[{{\rm{n}}}_{{\rm{hkl}}}/({{\rm{n}}}_{{\rm{hkl}}}-1)]}\}$$$$|{{\rm{I}}}_{{\rm{i}}}({\rm{hkl}})-\langle {\rm{I}}({\rm{hkl}})\rangle |$$/$${\sum }_{{\rm{hkl}}}\,\sum \,{{\rm{I}}}_{{\rm{i}}}({\rm{hkl}})$$

where I_i_(hkl) is the intensity of its i-th observation of reflection hkl including those of its symmetry mates. 〈I(hkl)〉 is the corresponding average intensity for all i measurements.

^d^Delta = 〈(I_+_ − I_−_)/σ(I)〉, is the average difference between the intensities of Bijvoet pairs, with respect to their standard deviation for the acentric (DeltaA), and the centric (DeltaC) reflections with R(ano) = DeltaA/DeltaC. I_+_ is the intensity for a reflection belonging to the set of symmetry equivalent reflections of reflection with indices (hkl) and I_-_ is the intensity for a reflection from the symmetry equivalent set of its Friedel mate (the symmetry equivalent set of the reflection with indices (−h, −k, −l)).

### Conserved disulfide bonds of F9

Poxviruses encode three proteins that form a unique cytoplasmic redox pathway responsible for the formation of intramolecular disulfide bonds in some poxvirus proteins. The disulfide bonds of both F9 and L1 are produced by this pathway^35^. F9 contains six cysteine residues that are conserved amongst all poxvirus orthologs. The atomic structure revealed that Cys33 and Cys55, Cys47 and Cys127, and Cys107 and Cys149 form three pairs of disulfide bonds. This follows a pattern in order of appearance in the sequence of 1–3, 2–5, and 4–6. Both Cys33 and Cys55 are located in loops at the top of the molecule. Cys47 is located in β2 and together with Cys127 it forms a disulfide bond, which stabilizes the β-sheet pairs at the bottom of the molecule. Both, Cys107 and Cys149 are found in α-helices. This disulfide bond stabilizes α4 and α5 (Fig. 2B, C).

### F9 and L1 share structural similarities but also reveal significant differences

**Figure 3:**
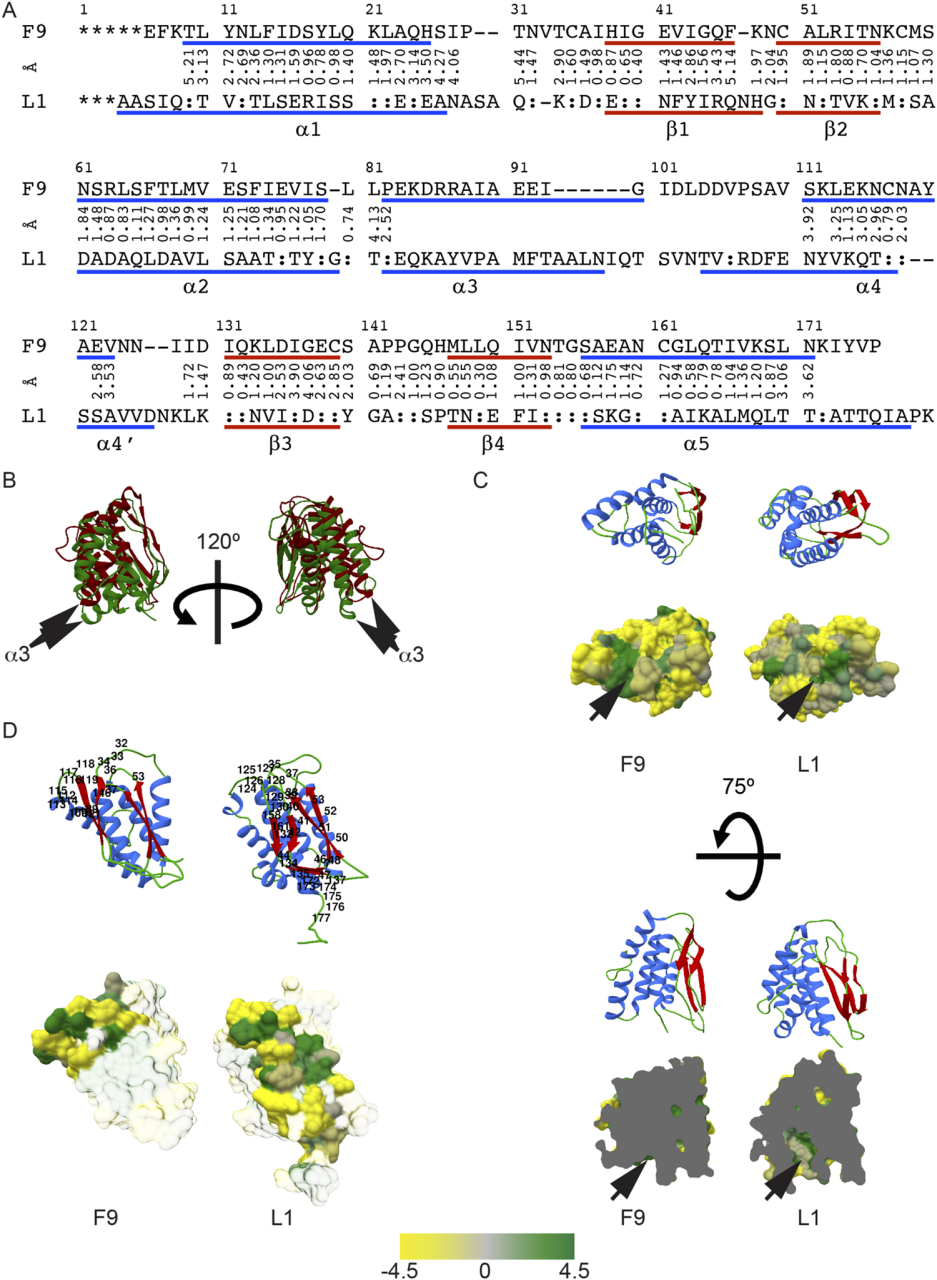
Three-dimensional structure alignment of F9 and L1 based on the α-carbons position. (**A**) Structural sequence alignment and distances of α-carbons in Å. Asterisks represent missing amino acid residues in the structure, dashes indicate gaps and colons label same amino acid residues. (**B**) The structural alignment of F9 and L1 calculated in PDBeFold^36^ was superimposed. F9 is displayed in red and L1 in green. The arrows label the α3 helices, which have different orientations and do not superimpose well in the overlaid models. (**C**) Two views of the L1 hydrophobic cavity presented in a surface and ribbon model. Upper part shows the entrance within a hydrophobic area from the bottom of the L1 and F9 molecule, while in the lower part both F9 and L1 were clipped from the side in the same way to make the cavity for the myristic moiety (black arrows) visible in L1. The hydrophobicity was calculated in Chimera according to Kyte and Doolittle^59^. Yellow (-4.5) represents hydrophilic areas while forest green (4.5) marks hydrophobic parts. (**D**) Residues predicted with meta-PPISP^37^ to mediate protein-protein interactions are numbered in the ribbon model. The hydrophobic surface presentation shows the same residues in opaque coloring.

The three-dimensional structure of F9 resembles the previously determined structure of L1. To reveal similarities and differences, we superimposed both structures using PDBeFold^36^ choosing the highest precision algorithm in the software. This resulted in a root-mean-square deviation (RMSD) of 2.06 Å calculated over 120 α-carbons (Fig. 3A). The orientations of the α3-helices were too different to be structurally aligned (Fig. 3A, B). In F9, α3 turns proximal whereas in L1 α3 is located sideward forming the entrance into the hydrophobic cavity (Fig. 3C, upper panel). This cavity is missing in F9, although a hydrophobic area similar to L1 is found (Fig. 3C, lower panel).

Putative protein-protein interaction sites were predicted with meta-PPISP^37^. For F9, residues Thr32-Ala34, His36, Ile37, Glu39, Asn53, Ala109, Tyr110, Glu112-Ile119, Lys121, and Glu146 involving β-strands 1 and 3 and helix α4 are predicted to be involved in protein-protein interactions. In L1, the prediction extends to β-strand 2 and the C-terminal part of the ectodomain including Ile7, Asp35, Glu37-Tyr42, Arg44, Asn46-Gly48, Asn50-Val53, Asn55, Asn124-Asn130, Ile132, Asp134, Glu135, Tyr137, Cys158, Lys161, and Thr172-Lys177 (Fig. 3D).

### Phylogenetic relationships between F9 and L1 orthologs

**Figure 4:**
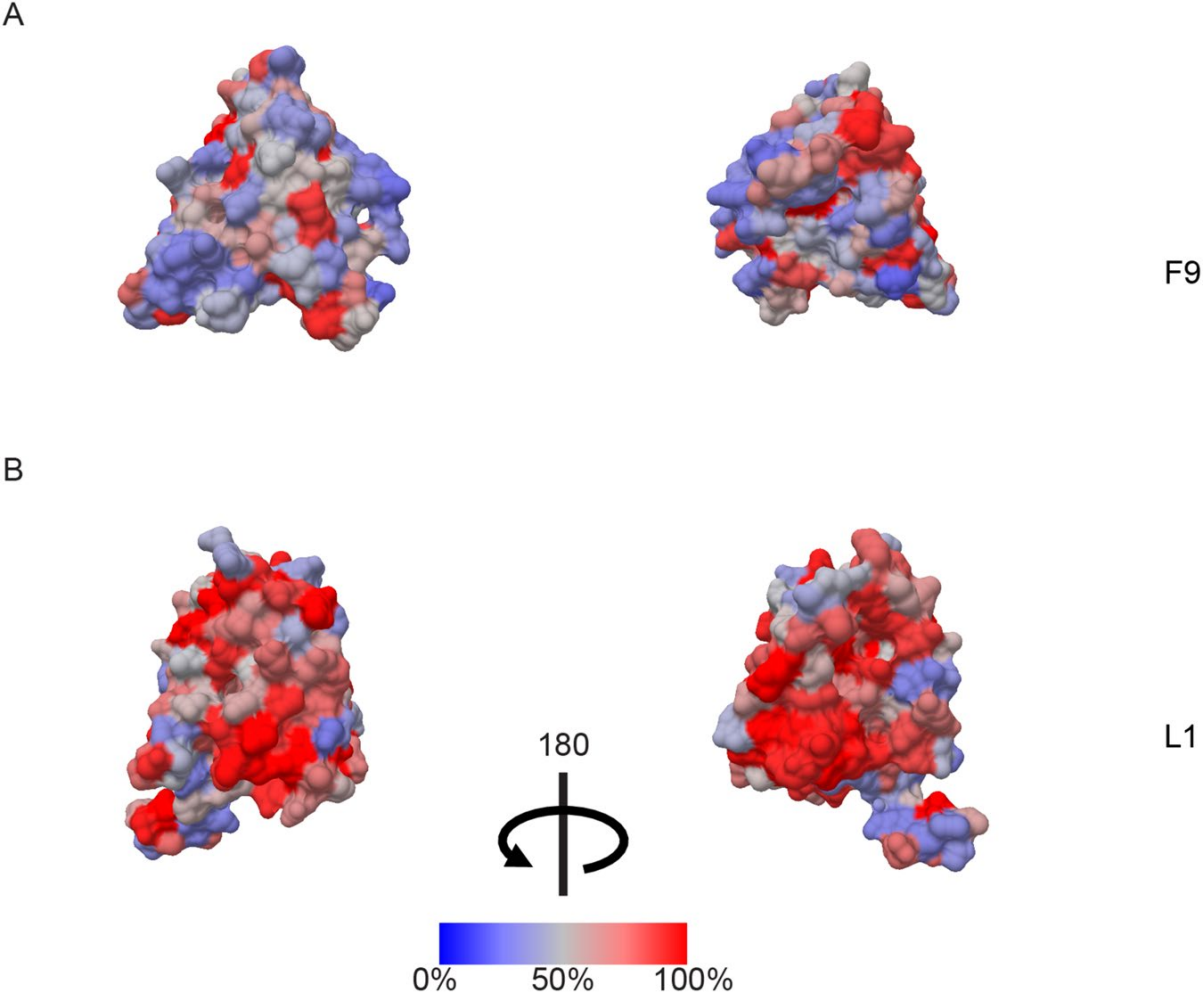
Percentage conservation of amino-acid residues in aligned F9 and L1 proteins (MSA, Supplemental file 1). Using a heat map display, the degree of amino acid conservation increases from blue to red. (**A**) Sequence conservation of F9 from two side views, (**B**) Sequence conservation of L1 from two side views.

The NCBI database was searched for full length *Poxviridae* F9 and L1 protein orthologs. The multiple sequence alignment (MSA, Supplemental file 1) generated with Multiple Alignment Using Fast Fourier Transformation (MAFFT^38^) revealed conserved amino acid residues and residues with conservative and semi-conservative changes in F9 and L1. The degrees of conservation were used to color the surface images of F9 and L1 in a heat map display or color ramp (Fig. 4).

We used the MSA to estimate the sequence identities in ClustalX (Supplemental Table 1). The lowest identity of 14% among all F9 orthologs was found between Nile crocodile poxvirus (YP_784226.1) and SGPV (YP_009162464.1). Between the F9 orthologs of Orthopoxviruses and SGPV the sequence identity was 21–23%. For L1 orthologs, the lowest identity of 20–21% was calculated between Orthopoxviruses and SGPV. In general, there were higher amino acid identities among the L1 ortholog sequences than among the F9 orthologs.

When F9 and L1 orthologs were compared to each other, the highest identity (24%) was found in Goatpoxvirus ‘Pellor’ (YP_001293215.1, YP_001293251.1). *Choristoneura biennis* entomopoxvirus had the lowest F9 to L1 identity of 14% (YP_008004349.1, YP_008004316.1). The identity between the full length F9 and L1 of VACV was 20%.

**Figure 5:**
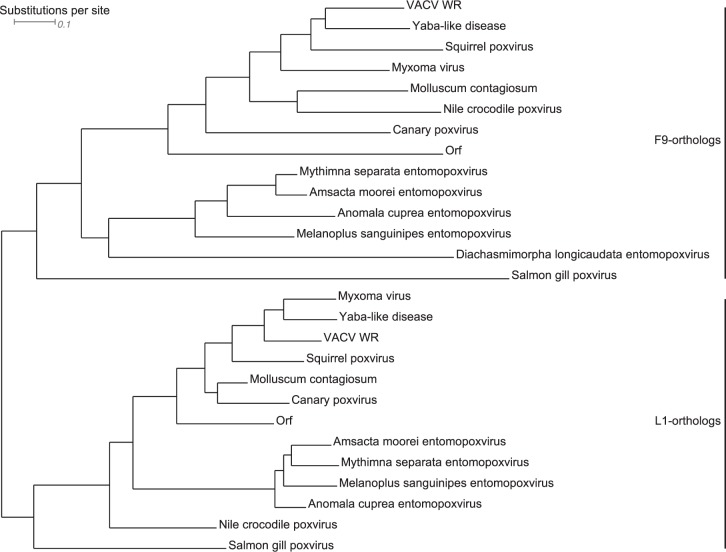
Phylogenetic tree of a selection of F9 and L1 orthologs in *Chordopoxvirinae* and *Entomopoxvirinae*. The root was forced between F9 and L1. The distance is given in substitutions per site. (The complete unrooted tree can be found in Supplemental file 2, Supplemental Fig. 2 presents the unrooted tree of the selected orthologs).

A phylogenetic tree was constructed with the neighbor joining method and 1,000 bootstraps using the conserved sites (90 amino acid residues). However, tree calculations including all gap free sites (177 amino acid residues) resulted in the same phylogeny with slightly increased distance values. The root was forced between F9 and L1, which form two distinctive clusters in an unrooted tree (Fig. 5, Supplemental Fig. 2, and Supplemental file 2). Clades are formed by orthologs of the respective genera. The F9 orthologs of Parapoxviruses exhibit a characteristic proline-rich N-terminal insertion of 14–23 amino acid residues (Supplemental file 1). SGPV possesses the most distant orthologs for both F9 and L1. VACV F9 and SGPV092 have a distance of 2.05 substitutions per site. In comparison, the distances of *Entomopoxvirinae* F9 orthologs to F9 are between 1.30 and 1.69 substitutions per site. Interestingly, the distance of F9 to SGPV097, the L1 ortholog, is only 1.53 substitutions per site. In case of VACV L1, the distance to SGPV097 is 1.17 substitutions per site. VACV L1 and its orthologs in *Entomopoxvirinae* have a distance of 0.88 to 1.03 substitutions per site.

### Comparison of VACV F9 and L1 with the SGPV homologs

**Figure 6:**
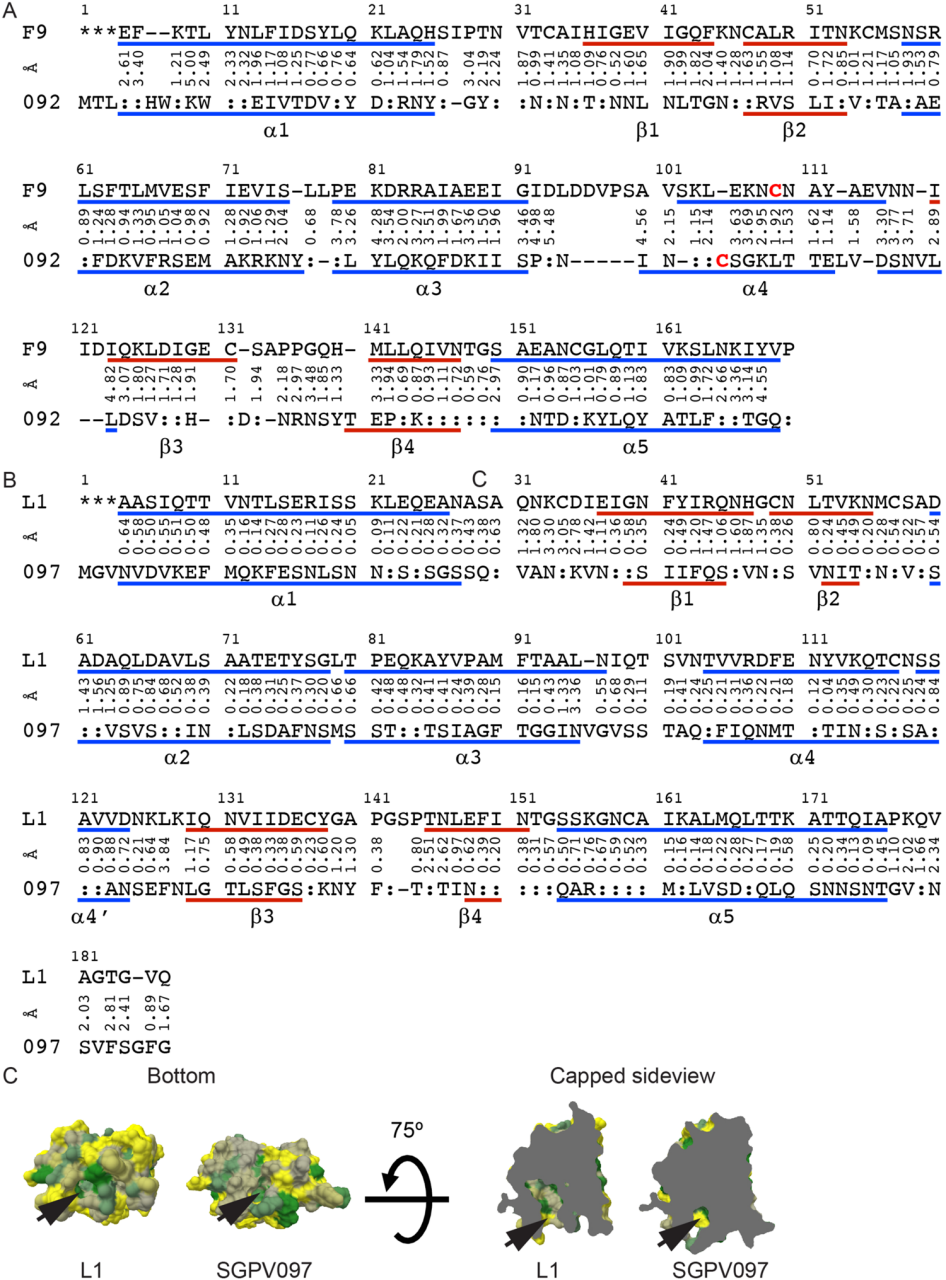
Structural modeling of SGPV092 and SGPV097. (**A**) Sequence alignment based on the three-dimensional models of SGPV092 (092) to F9. Blue lines indicate calculated β-sheets and red lines α-helices. The non-aligned Cys residues are marked in red and bold. (**B**) Sequence alignment based on the three-dimensional models of SGPV097 (097) to L1. Blue lines indicate calculated β-sheets and red lines α-helices. (**C**) Comparing the hydrophobic cavities of SGPV097 and L1. No myristoylation motif was present in SGPV097. Bottom view of the molecules (upper) and clipped side view (lower) of the cavity. The hydrophobicity is scaled with yellow hydrophilic (−4.5) to forest green hydrophobic (4.5).

In order to compare F9 from VACV WR with its most distantly related homolog SGPV092, the three-dimensional structure of SGPV092 was modeled using the F9 structure as a template for threading with iTASSER^39^. The protein sequences were aligned and cysteines in SGPV092 were presumed to form disulfide bonds as in F9. Distances between relevant cysteines forming disulfides were restricted to 2.00 Å in iTASSER. We then superimposed both structures in PBDeFold (Fig. 6A) resulting in an RMSD of 2.13 Å (n = 143 α-carbons). The strands β1 and β3 are predicted to be less structurally defined in SGPV092 than F9. The fourth cysteine does not align well structurally between F9 and SGPV092. It is found more N-terminal at position 97 in SGPV092 as compared to position 107 in F9. Position 97 in SGPV092 is located within the loop between α3 and α4. In addition, the model predicts a twisted conformation of the polypeptide chain within the loop. Assuming the cysteine at SGPV092 position 97 forms a disulfide with Cys142, as the corresponding cysteines do in F9, the loop will be shorter and more rigid in the SGPV protein.

In contrast to F9 and SGPV092, the structure of L1 and the predicted structure of SGPV097 align closely with an RSMD of 0.96 Å over the entire ectodomain (Fig. 6B). The secondary structures are similarly defined with the cysteine residues aligned in the same regions. Greater distances between α-carbons up to 3.84 Å RMSD were only found in loops. Phe108, located at the end of the cavity in L1 and required for structural integrity^33^, is conserved in all L1 orthologs except SGPV097.

## Discussion

Most viruses encode just one or two proteins that mediate membrane fusion and virus entry. Poxviruses are unique in requiring eleven transmembrane proteins that form the entry fusion complex (EFC). In the present study, we determined the protein structure of F9, one of the eleven EFC proteins. We demonstrated through X-ray crystallography that the overall structure of F9 is a bundle of α-helices leaning towards and packed against two pairs of β-sheets. Three disulfide bonds covalently link the helices and sheets and are not solvent accessible, which was anticipated due to the relatively reducing cytoplasmic environment where F9 is expressed. F9 exhibits a distinct protein fold not found in structures other than L1 as determined using the DALI server^40^ and a Z-score cut-off value of 5. The next closest structures to F9 after L1 overlap in a few helices with Z-scores less than 4.6.

F9 resembles the structure of L1^32^. The DALI comparison of F9 and L1 (PDB code: 1ypy) has a Z-score of 15. Despite their similarity, F9 and L1 are each required for fusion and entry of the viral core suggesting distinct functions^13,28,35^. Within the EFC, there are two groups of sequence-related proteins: A16, G9, J5, and F9, L1. These two groups of related proteins are present in all members of both the Chordopoxvirus and Entomopoxvirus subfamilies suggesting that their sequences and functions diverged early in poxvirus evolution. With regard to the divergence of F9, its differing orientation of helix α3, its absence of a myristoylation site, and its lack of a hydrophobic cavity likely contribute to F9 functional differences from L1. Nevertheless, the comparable parts of the α-helices of F9 and L1 align with an RMSD of 1.77 Å. The best overlap between F9 and L1 was found within the β-sheet region with an RMSD of 1.68 Å over all four β-strands.

Some residues have been predicted to be involved in protein-protein interactions. This hydrophobic surface area might interact with other VACV EFC proteins for quaternary structure formation. Larger hydrophobic interfaces have been described for obligate complexes^41^. These regions are also highly hydrophobic and therefore might be shielded from aqueous solvent by binding other members of the EFC. However, there is no evidence that F9 and L1 are interacting directly with each other in the EFC^18^. Although the large interaction surface between the β-sheets areas of adjacent F9 molecules (1478 Å^2^ total buried area) and L1 molecules (1081 Å^2^ total buried area) in the crystals suggest a homodimer formation, their biological relevance, if any, still has to be proven. In F9, the area predicted to interact with proteins comprises α4. A stabilization within that surface would also provide a stable environment for the emerging loop but allow flexibility within the α1-α3, and β1 and β2 area.

A database search to determine the conservation of full length F9 and L1, revealed a higher degree of conservation in L1. We chose to model the most distant orthologs of F9 and L1 to estimate structural conservation. Analyses by Kaczanowski and Zielenkiewicz^42^ indicated that the three-dimensional structure of proteins is evolutionary more conserved than expected by sequence homology. The phylogenetic analyses suggested that the F9 ortholog in SGPV, SGPV092 (2.0504 substitutions/site), is even more distant to VACV WR F9 than the subfamily of *Entomopoxvirinae* (1.3044 to 1.6919 substitutions per site). The results are also supported by the identities calculated with ClustalX^43^. Differences in the structures of the VACV and SGPV orthologs of F9 are predicted within the loop region between α3 and α4. Assuming all disulfide bonds are formed, the protruding loop formed in F9 would be significantly shorter and partly defined in a helical formation in SGPV092. Furthermore, there is no Gly in SGPV092 corresponding to Gly90 in F9 that might allow the formation of a longer protruding loop by breaking the α-helix.

The only other F9 ortholog without a Gly in this area is from *Diachasmimorpha longicaudata* entomopox. The L1 ortholog in SGPV (SGPV097) does not exhibit a predicted N-terminal myristoylation site. The threaded structural model shows a small cavity not large enough to incorporate a myristic moiety. In addition, the Phe108 (L1) which is located at the end of the hydrophobic cavity might have a counterpart in Phe105 (SGPV097) that is modeled at the N-terminal end of α4 and would, therefore, not be facing the inside of the hydrophobic cavity. Phe108 of L1 is conserved in all other poxvirus orthologs and shown to be essential for conformation and complete disulfide bonding^33^. The N-terminal modification by myristate and the formation of a hydrophobic cavity in L1 for burying the myristic acid leads to a higher degree of conservation in L1 because of these constraints on sequence changes.

It should be noted that no orthologs for G3 and L5 were found in the SGPV genome^21^. We also screened the SGPV genome for the presence of the O3 protein, another component of the EFC. No related sequence was found by alignment nor was a putative O3-like hydrophobic protein identified within the haplotype block. With the differences in F9 and the absence of G3, L5, and O3, Salmon gill poxvirus (SGPV) may have developed a modified version of the poxvirus EFC.

## Materials and Methods

### Expression of F9

DNA encoding the first 176 amino acid residues of vaccinia virus (VACV) Western Reserve F9, lacking the transmembrane segment, and containing nucleotides for an N-terminal 6xHis tag, spacer and TEV protease cleavage site was codon optimized for *E. coli* and synthesized by Blue Heron Biotech in the expression vector pNAN^44^. The plasmid was transformed into NiCo21(DE3) cells (New England BioLabs). Cultures were grown until OD_600nm_ of 0.5–0.6 and the T7 promoter-driven protein expression was induced with the addition of 1 mM isopropyl β-D-thiogalactoside (IPTG). Cells were harvested by centrifugation and the cell pellet washed once with resuspension buffer (50 mM Tris-HCl pH 8.0, 25% sucrose, 0.1% NaN_3_, 1 mM EDTA, 10 mM 1,4-dithiothreitol (DTT)). The cell pellet was suspended in resuspension buffer and stirred for 1 h. Five volumes of lysis buffer (50 mM Tris-HCl pH 8.0, 1% Triton X-100, 1% Na-deoxycholate, 0.1% NaN_3_, 100 mM NaCl, 10 mM (DTT), 5 mM MgCl_2_) with 1 mg/ml lysozyme and 750 U benzonase were added. Samples were stirred for 1 h at room temperature. Further cell lysis was achieved by three cycles of freeze-thawing. The inclusion body fraction was harvested by centrifugation, then washed and homogenized three-times in lysis buffer without MgCl_2_. After three washes in wash buffer (50 mM Tris-HCl, 0.1% NaN_3_, 100 mM NaCl, 1 mM EDTA, 10 mM DTT) inclusion bodies were denatured overnight at 4 °C in 100 mM Tris-HCl, pH 8.0, 6 M guanidine-HCl, 10 mM DTT, 1 mM EDTA. Insoluble material was removed by centrifugation at 10,000 × g for 1 h at 4 °C. Inclusion bodies were stored at −80 °C until use.

### Refolding and Purification of F9

Approximately 10 µmol F9 inclusion bodies were refolded by rapid dilution into 1 l of 50 mM Tris-HCl, pH 8.0, 50 mM NaCl, 500 mM arginine, 2 mM EDTA, 40 mM sucrose, 10 mM DTT, 5 mM cystamine-HCl. After refolding at 4 °C overnight, 50 mM cystamine-HCl was added to react with free cysteines. The solution was dialyzed twice against 50 mM Tris-HCl, 50 mM NaCl, pH 8.0. After filtration through a 22 µm filter membrane, F9 protein was loaded onto a Ni-NTA column (GE) equilibrated with 50 mM Tris-HCl, 50 mM NaCl, pH 8.0. The column was washed with three column volumes of equilibration buffer. Bound F9 was eluted with a linear increasing imidazole gradient. Fractions containing eluted protein were combined and dialyzed against 50 mM Tris-HCl, 50 mM NaCl, pH 8.0 overnight to remove imidazole. The concentration was measured spectrophotometrically at 280 nm using a molar extinction coefficient of 10,805 M^−1^cm^−1^. To remove the N-terminal tag, F9 protein was incubated with TEV protease (10:1 F9 to protease by weight) overnight at room temperature. Uncleaved F9 and recombinant protease were removed by binding to Ni-NTA. Highly pure F9 was obtained by SEC using a Superdex-200 column and 10 mM Tris-HCl, 50 mM NaCl, pH 8.0 running buffer. The monomeric fraction was collected and concentrated to 10 mg/ml with centrifugal filter units (EMD). After removing precipitated material by high-speed centrifugation, the integrity of the concentrated protein was monitored by dynamic light scattering. The purity of the protein was >95% as assessed by SDS-polyacrylamide gel electrophoresis.

### Expression and purification of recombinant tobacco etch virus (TEV) protease

Plasmid pRK793 (Addgene) encoding recombinant 6 × His-TEV(S219V)-5 × Arg protease was transformed into NiCo21(DE3) cells. Production and purification was done according to Tropea *et al*.^45^. Briefly, the TEV protease was produced as a fusion with maltose binding protein (MBP), which cleaves itself from the fusion *in vivo*. The resulting soluble TEV protease was released from the cells by sonication and purified with Ni-NTA chromatography. Eluted TEV protease was dialyzed and subsequently loaded onto a Superdex column. Concentrated TEV protease was flash frozen in liquid nitrogen and stored at −80 °C until use. The purified protease was concentrated to ~1.5 mg/ml and flash frozen in liquid nitrogen.

### Crystallization of F9

Native crystals were obtained in hanging drops by vapor diffusion with two buffer conditions. One mother liquor contained 3.0–3.2 M NaCl and 0.1 M 2-(N-morpholino)ethanesulfonic acid (MES). For cryo-protection and phasing the crystals were incubated in 3.5 M NaCl, 0.1 M MES, 1 M NaI for 30 s at room temperature. Crystals were also obtained from a solution containing 25% PEG 8000, 10% ethanol, 1–5% cocktail mixture of low molecular alcohols, 30% sucrose at pH 8.5 buffered with Tris in the presence of 50 mM NaCl. The drops were set up with 1 µl protein and 1 µl mother liquor. For X-ray data collection, crystals were harvested and flash frozen in liquid nitrogen.

### Data Collection and structure determination

X-ray data on F9 crystals rapidly soaked with NaI^46,47^ were collected on a RIGAKU MicroMax-002 + high-intensity microfocus sealed tube X-ray generator equipped with an R-Axis IV++ image plate detector using Cu radiation. The data processed with XDS^48^ showed the presence of a strong anomalous signal (Table 1) and were supplied to the AutoSol module of the Phenix software package^49^ for phasing by single anomalous diffraction (SAD). AutoSol located nine iodide sites, and anomalous difference maps confirmed that the space group was P4_1_22. Auto building of the F9 protein model was able to locate two F9 molecules per asymmetric unit, which was consistent with the prediction based on the calculation of the Matthews coefficient^50–52^. The auto-built model was 60% complete. This partial model was further improved by employing cycles of refinement using the programs CNS^53^ and Phenix followed by manual model rebuilding using the programs O^54^ and Coot^55^ into new electron density maps calculated after each refinement cycle until no further improvement of the model could be achieved at the 3.20 Å maximum resolution of the P4_1_22 data set. This model was subsequently used as a search model for obtaining a molecular replacement solution in a 2.10 Å data set from an F9 crystal that belonged to the P2_1_2_1_2_1_ space group and contained four molecules in the asymmetric unit. Multiple cycles of refinement with stepwise increase in the resolution followed by manual rebuilding using the same programs as above produced the final model that includes residues 6–164 for all four molecules with an Rwork/Rfree of 22.57/26.85 and good geometry. The F9 structure as accession code 6CJ6 was submitted to the RCSB Protein Data Bank at www.rcsb.org.

### Comparison of the F9 and L1 atomic structures

The F9 chain A was set as query and the chain A of L1 database entry 1ypy was used as the target in PDBeFold^36^. The precision was set on ‘highest’ to calculate the RMSD and the structural alignment. The output pdb-files were used to visualize the alignment in Chimera^56^. Protein-protein interaction sites for comparison of both structures and for comparison to contact areas within the crystals were predicted with meta-PPISP^37^.

### Phylogenetic analysis of F9 and L1 in *Poxviridae*

Using BLASTp^57^ and full-length F9 or L1 from VACV WR as query sequence, we set searching parameters to BLOSUM45 (more divergent alignments) and organism *Poxviridae*. Partial sequences were removed from the sequence data sets. Sequences of L1 orthologs recovered together with the F9 sequence set were identified by their myristoylation site and removed and vice versa. The collected sequences were aligned in MAFFT^38^ with BLOSUM45 and ‘leaving gappy regions’. The MSA was used to identify the conservation state of each residue. Using a proportional gradient, the surface conservation was visualized with Chimera^56^. The MSA was also entered into ClustalX^43^ to calculate the pairwise % sequence identity scores. We used the sequence identity to estimate the relationship between F9 and L1 orthologs within one virus species. Phylogenetic trees were constructed by the Neighbor joining method. The confidence values of nodes were calculated with 1,000 bootstrap replications. The SGPV L1 ortholog SGPV097 did not present a predictable myristoylation site. SGPV F9 homolog SGPV092 and SGPV097 were present in both dataset and used as outgroups to root the respective trees. After combining both datasets and aligning F9 and L1 orthologs together, the root of the phylogenetic tree was forced between F9 and L1 orthologs. Dendroscope^58^ (www.dendroscope.org) was used for visualization.

### Three-dimensional modeling of SGPV092 and SGPV097

Atomic structures of F9 and L1 were used as query structures to obtain a model of the respective SGPV proteins using iTASSER. The most likely model 1 was processed. In the case of F9, both proteins were aligned using BLOSUM45 and distances of Cys in SGPV092, which correspond to disulfide bonds in F9, were restricted to 2.00 Å. More constraints for modeling SGPV092 on F9 were necessary to obtain closed disulfide bonds. Senkevich *et al*.^35^ have shown that missing disulfide bonds destabilize the protein and thus impair its function. VACV F9 and SGPV092 as well as VACV L1 and SGPV097 were superimposed by their structures using PBDeFold with ‘highest’ precision. The VACV proteins were defined as query and the SGPV sequences were defined as target. The three-dimensional structural alignments were collected in a RMSD distance matrix.

## Electronic supplementary material

Supplemental Material

Supplemental table 1

Quality report

## Data Availability

All data generated or analyzed during this study are included in this manuscript and its Supplementary information files.
