# Supplementary material for: The 2.1 Å structure of protein F9 and its comparison to L1, two components of the conserved poxvirus entry-fusion complex
Source: Sci Rep. 2018 Nov 14;8:16807. doi: 10.1038/s41598-018-34244-7 (PMC6235832; doi:10.1038/s41598-018-34244-7)

The 2.1 Å structure of protein F9 and its comparison to L1, two components of the conserved poxvirus entry-fusion complex

Ulrike S. Diesterbeck[1]*, Apostolos G. Gittis[2], David N. Garboczi[2], Bernard Moss[1]

[1] Laboratory of Viral Diseases, National Institute of Allergy and Infectious Diseases, National Institutes of Health, Bethesda, Maryland, USA

[2] Structural Biology Section, Research Technologies Branch, National Institute of Allergy and Infectious Diseases, National Institutes of Health, Rockville, Maryland, USA.

* Correspondence: Ulrike.Diesterbeck@gmail.com

Legends to Supplemental files

Supplemental file 1: Multiple sequence alignment of full length F9 and L1 orthologs. For the alignment, the BLOSUM45 matrix was used and aligned with MAFFT [38]. The default parameters were changed to 'leave gappy regions'.

Supplemental file 2: Unrooted phylogenetic trees in Newick format calculated by Neighbor joining method and 1000 Bootstraps using 90 conserved amino acid residues (A, F9_L1_conserved) or 177 amino acid residues in all gap-free sites (B, F9_L1_gapfree) and the multiple sequence alignment of Supplemental file 1.

Legend to Supplemental table

Supplemental table 1: The pairwise sequence identities from the multiple sequence alignment (Supplemental file 1) were calculated using ClustalX [43].

Legend to Supplemental figures

Supplemental figure 1: Unmodified and scanned Coomassie gels to Figure 1.

Supplemental figure 2: Unrooted tree of F9 and L1 orthologs. The same dataset as presented in Figure 5 was used. Arrows display the division of branching F9 and L1 orthologs.

Supplemental file 1

```
>Query_119423
----------------MAETKEFKTLYNLFIDSYLQKLAQH-SIPTNVTCAIHIGEVIGQ F-
KNCALRITNKCMSNSRLSFTLMVESFIEVISLLPEKDRRAIAEEIG-IDL-------D
DVPSAVSKLEKNC--N---AYAEVNNIIDIQK-LDIGECSAPPGQ-HMLLQIVNTGSAEA
NCGLQTIVKSLNKIYVPPI--IENRLPYYD-------------PWFL---VGVA-IILVI
FTVAICSIR---------RNLALKYRYGTFLYV---------------------------
------------------
>gi|66275845|ref|YP_232930.1| SS bond formation pathway protein
[Vaccinia virus]
----------------MAETKEFKTLYNLFIDSYLQKLAQH-SIPTNVTCAIHIGEVIGQ F-
KNCALRITNKCMSNSRLSFTLMVESFIEVISLLPEKDRRAIAEEIG-IDL-------D
DVPSAVSKLEKNC--N---AYAEVNNIIDIQK-LDIGECSAPPGQ-HMLLQIVNTGSAEA
NCGLQTIVKSLNKIYVPPI--IENRLPYYD-------------PWFL---VGVA-IILVI
FTVAICSIR---------RNLALKYRYGTFLYV---------------------------
------------------
>gi|325558218|gb|ADZ29598.1| SS bond formation pathway protein [Cowpox
virus]
----------------MAETKEFKTLYNLFIDSYLQKLAQH-SIPTNVTCAIHIGEVIGQ F-
KNCALRITNKCMSNSRLSFTLMIESFIEVISLLPEKDRRAIAEEIG-IDL-------D
DVPSAVSKLEKNC--N---AYAEVNNIIDIQK-LDIGECSAPPGQ-HMLLQIVNTGSAEA
NCGLQTIVKSLNKIYVPPI--IENRLPYYD-------------PWFL---VGVA-IILVI
FTVAICSIR---------RNLALKYRYGTFLYV---------------------------
------------------
>gi|325558002|gb|ADZ29383.1| SS bond formation pathway protein [Cowpox
virus]
----------------MAETKEFKTLYNLFIDSYLQKLAQH-SIPTNVTCAIHIGEVIGQ F-
KNCALRITNKCMSNSRLSFTLMVESFIEVISLLPEKDRRAIAEEIG-IDL-------D
DVPSAVSKLEKNC--N---AYAEVNNIIDIQK-LDIGECSAPPGQ-HMLLQIVNTGSAEA
NCGLQTIVKSLNKIYIPPI--IENRLPYYD-------------PWFL---VGVA-IILVI
FTVAICSIR---------RNLALKYRYGTFLYV---------------------------
------------------
>gi|325514063|gb|ADZ24057.1| SS bond formation pathway protein [Cowpox
virus]
----------------MAETKEFKTLYNLFIDSYLQKLAQH-SIPTNVTCAIHIGEVIGQ F-
KNCALRITNKCMSNSRLSFTLMVESFIEVISLLPEKDRRAIAEEIG-IDL-------D
DVPSAVSKLEKNC--N---AYAEVNNIIDIQK-LDIGECSAPPGQ-HILLQIVNTGSAEA
NCGLQTIVKSLNKIYVPPI--IENRLPYYD-------------PWFL---VGVA-IILVI
FTVAICSIR---------RNLALKYRYGTFLYV---------------------------
------------------
>gi|1169132833|gb|ARB50282.1| SS bond formation pathway protein
[Cowpox virus]
----------------MAETKEFKTLYNLFIDSYLQKLAQH-SIPTNVTCAIHIGEVIGQ F-
KNCALRITNKCMSNSRLSFTLLIESFIEVISLLPEKDRRAIAEEIG-IDL-------D
DVPSAVSKLEKNC--N---AYAEVNNIIDIQK-LDIGECSAPPGQ-HMLLQIVNTGSAEA
NCGLQTIVKSLNKIYVPPI--IENRLPYYD-------------PWFL---VGVA-IILVI
FTVAICSIR---------RNLALKYRYGTFLYV---------------------------
------------------
>gi|111184232|gb|ABH08152.1| HSPV049 [Horsepox virus]
----------------MAETKEFKTLYNLFIDSYLQKLAQH-SIPTNVTCAIHIGEVIGQ F-
KNCALRITNKCMSNSRLSFTLMVESFIEVISLLPEKDRRAIAEEIG-IDL-------D
DVPSAVSKLEKNC--N---AYAEVNNIIDIQK-LDIGKCSAPPGQ-HMLLQIVNTGSAEA
NCGLQTIVKSLNKIYVPPI--IENRLPYYD-------------PWFL---VGVA-IILVI
FTVAICSIR---------RNLALKYRYGTFLYV---------------------------
------------------
>gi|554571866|gb|AGY97569.1| CPXV056 protein [Cowpox virus]
----------------MAETKEFKTLYNLFIDSYLQKLAQH-SIPTNVTCAIHIGEVIGQ F-
KNCALRITNKCMSNSRLSFTLMVESFIEVISLLPEKDRRAIAEEIG-IDL-------D
DVPSAVSKLEKNC--N---AYAEVNNIIDIQK-LNIGECSAPPGQ-HMLLQIVNTGSAEA
```

```
NCGLQTIVKSLNKIYVPPI--IENRLPYYD-------------PWFL---VGVA-IILVI
FTVAICSIR---------RNLALKYRYGTFLYV---------------------------
------------------
>gi|22164637|ref|NP_671550.1| EVM032 [Ectromelia virus]
----------------MAETKEFKTLYNLFIDSYLQKLAQH-SIPTNVTCAIHIGEVIGQ F-
KNCALRITNKCMSNSRLSFTLMVESFIEVISLLPEKDRRAIAEEIG-IDL-------D
DVPSAVSKLEKNC--N---AYAEVNNIIDIQK-LNIGECSAPPGQ-HMLLQIVNTGSADA
NCGLQTIVKSLNKIYVPPI--IENRLPYYD-------------PWFL---VGVA-IILVI
FTVAICSIR---------RNLALKYRYGTFLYV---------------------------
------------------
>gi|38348914|gb|AAR17890.1| SS bond formation pathway protein
[Vaccinia virus]
----------------MAETKEFKTLYNLFIDSYLQKLAQH-SIPTNVTCAIHIGEVIGQ F-
KNCALRITNKCMSNSRLSFTLMVESFIEVISLLPEKDRRAIAEEIG-IDL-------D
DVPSVVSKLEKNC--N---AYAEVNNIIDIQK-LDIGECSAPPGQ-HMLLQIVNTGSAEA
NCGLQTIVKSLNKIYVPPI--IENRLPYYD-------------PWFL---VGVA-IILVI
FTVAICSIR---------RNLALKYRYGTFLYV---------------------------
------------------
>gi|17974954|ref|NP_536468.1| C15L [Monkeypox virus Zaire96I16]
----------------MAETKEFKTLYNLFIDSYLQKLAQH-SIPTNVTCAIHIGEVIGQ F-
KNCALRITNKCMSNSRLSFTLMVESFIEVISLLPEKDRRAIAEEIG-IDL-------D
DVPSVVSKLEKNC--N---AYAEVNNIIDIQK-LNIGECSAPPGQ-HMLLQIVNTGSAEA
NCGLQTIVKSLNKIYVPPI--IENRLPYYD-------------PWFL---VGVA-IILVI
FTVAICSIR---------RNLALKYRYGTFLYV---------------------------
------------------
>gi|44971400|gb|AAS49750.1| RPXV037 [Rabbitpox virus]
----------------MAETKEFKTLYNLFIDSYLQKLAQH-SIPTNVTCAIHIGEVIGQ F-
KNCALRITNKCMSNSRLSFTLMVESFIEVISLLPEKDRRAIAEEIG-IDL-------D
DVPSAVSKLEKNC--N---AYAEVNNIIDIQK-LDIGECSAPPGQ-HMLLQIVNTGSAEA
NCGLQTIVKSLNKIYVPPI--IENRLPYYD-------------PWFL---VGVV-IILVI
FTVAICSIR---------RNLALKYRYGTFLYV---------------------------
------------------
>gi|1210076734|emb|SNB49914.1| CPXV056 protein [Cowpox virus]
----------------MAETKEFKTLYNLFIDSYLQKLAQH-SIPTNVTCAIHIGEVIGQ F-
KNCALRITNKCMSNSRLSFTLMIESFIEVISLLPEKDRRAIAEEIG-IDL-------D
DVPSAVSKLEKNC--N---AYAEVNNIIDIQK-LDIGECSAPPGQ-HMLLQIVNTGSAEA
NCGLQTIVKSLNKIYVPPI--IENRLPYYD-------------PWFL---VGVA-IILVI
FTVAICSIR---------RNLDLKYRYGTFLYV---------------------------
------------------
>gi|20178423|ref|NP_619844.1| CPXV056 protein [Cowpox virus]
----------------MAETKEFKTLYNLFIDSYLQKLAQH-SIPTNVTCAIHIGEVIGQ F-
KNCALRITNKCMSNSRLSFTLMVESFIEVISLLPEKDRRAIAEEIG-IDL-------N
DLPSAVSKLEKNC--N---AYAEVNNIIDIQK-LDIGECSAPPGQ-HMLLQIVNTGSAEA
NCGLQTIVKSLNKIYIPPI--IENRLPYYD-------------PWFL---VGVA-IILVI
FTVAICSIR---------RNLALKYRYGTFLYV---------------------------
------------------
>gi|90660286|gb|ABD97400.1| unknown [Cowpox virus]
----------------MAETKEFKTLYNLFIDSYLQKLAQH-SIPTNVTCAIHIGEVIGQ F-
KNCALRITNKCMSNSRLSFTLMVESFIEVISLLPEKDRRAIAEEIG-IDL-------D
DVPSAVSKLEKNC--N---AYAEVNNIIDIQK-LDIGECSAPPGQ-HMLLQIVNTGSAEA
NCGLQTIVKSLNKIYVPPI--IENRLPYYD-------------PWFL---VCVA-IILVI
FTVAICSIR---------RNLALKYRYGTFLYV---------------------------
------------------
>gi|137849|sp|P24361.1|F9_VACCW RecName: Full=Protein F9
----------------MAETKEFKTLYNLFIDSYLQKLAQH-SIPTNVTCAIHIGEVIGQ F-
KNCALRITNKCMSNSRLSFTLMVESFIEVISLLPEKDRRRIAEEIG-IDL-------D
DVPSAVSKLEKNC--N---AYAEVNNIIDIQK-LDIGECSAPPGQ-HMLLQIVNTGSAER
NCGLQTIVKSLNKIYVPPI--IENRLPYYD-------------PWFL---VGVA-IILVI
FTVAICSIR---------RNLALKYRYGTFLYV---------------------------
------------------
```

>gi|18640278|ref|NP_570434.1| CMLV044 [Camelpox virus]
----------------MAETKEFKTLYNLFIDSYLQKLAQY-SIPTNVTCAIHIGEVIGQ F-
KNCALRITNKCMSNSRLSFTLMVESFIEVISLLPEKDRRVIAEEIG-IDL-------D
DVPSAVSKLEKNC--N---AYAEVNNIIDIQK-LNIGECSAPPGQ-HMLLQIVNTGSAEA
NCGLQTIVKSLNKIYVPPI--IENRLPYYD-------------PWFI---VGVA-IILVI
FTVAICSIR---------RNLALKYRYGTFLYV---------------------------

>gi|113195226|ref|YP_717356.1| hypothetical protein TATV_DAH68_049
[Taterapox virus]
----------------MAETKEFKTLYNLFIDSYLQKLAQH-S-PTNVTCAIHIGEVIGQ F-
KNCALRITNKCMSNSRLSFTLMVESFIEVISLLPEKDRRAIAEEIG-IDL-------D
DVPSVVSKLEKNC--N---AYAEVNNIIDIQK-LNIGECSAPPGQ-HMLLQIVNTGSAEA
NCGLQTIVKSLNKIYVPPI--IENRLPYYD-------------PWFL---VGVA-IILVI
FTVAICSIR---------RNLALKYRYGTFLYV---------------------------

>gi|9627554|ref|NP_042077.1| SS bond formation pathway protein
[Variola virus]
----------------MAETKEFKTLYNLFIDSYLQKLAQH-SIPTNVTCAIHIGEVIGQ F-
KNCALRITNKCMSNTRLSFTLMVESFIEVISLLPEKDRRAIAEEIG-IDL-------N
DVPSAVSKLEKNC--N---AYAEVNNIIDIQK-LNIGECSAPPGQ-HMLLQIVNTGSAGA
NCGLQTILKSLNKIYVPPI--IENRLPYYD-------------PWFL---VGVA-IILVI
FTVAICSIR---------RNLALKYRYGTFLYV---------------------------

>gi|94487114|gb|ABF26214.1| hypothetical protein VARV_KUW67_1629_036
[Variola virus]
----------------MAETKEFKTLYNLFIDSYLQKLAQH-SIPTNVTCAIHIGEVIGQ F-
KNCALRITNKCMSNTRLSFTLMVESFIEVISLLPEKDRRAIAEEIG-IDL-------N
DVPSAVSKLEKNC--N---AYAEVNNIIDIQK-LNIGECSAPPGQ-HMLLQIVNTGSAGA
NCGLQTILKSLNKIYVPPI--IENRLPYYE-------------PWFL---VGVA-IILVI
FTVAICSIR---------RNLALKYRYGTFLYV---------------------------

>gi|544838|gb|AAB29629.1| C13L product [variola virus VAR, India1967,
Peptide, 212 aa]
----------------MAETKEFKTLYNLFIDSYLQKLAQH-SIPTNVTCAIHIGEVIGQ F-
KNCALRITNKCMSNTRLSFTLMVESFIEVISLLPEKDRRAIAEEIG-IDL-------N
DVPSAVSKLEKNC--N---AYAEVNNIIDIQK-LNIGECSAPPGQ-HMLLQIVNTGSAGA
NCGLDTILKSLNKIYVPPI--IENRLPYYD-------------PWFL---VGVA-IILVI
FTVAICSIR---------RNLALKYRYGTFLYV---------------------------

>gi|94489329|gb|ABF28418.1| hypothetical protein VARV_UNK44_harv_036
[Variola virus]
----------------MAETKEFKTLYNLFIDSYLQKLAQH-SIPTNVTCAIHIGEVIGQ F-
KNCALRITNKCMSNTRLSFTLMVESFIEVISLLPEKDRRAIAEEIG-IDL-------N
DVPSAVSKLEKNC--N---AYAEVNNIIDIQK-LNIEECSAPPGQ-HMLLQIVNTGSAGA
NCGLQTILKSLNKIYVPPI--IENRLPYYD-------------PWFL---VGVA-IILVI
FTVAICSIR---------RNLALKYRYGTFLYV---------------------------

>gi|831934475|ref|YP_009143355.1| SS bond formation pathway protein
[Raccoonpox virus]
----------------MAETKEFKTLYNLFIDSYLQKLAQN-SIPTNVTCAIHIGEVVGK F-
KNCALRITNKCMSNSRLSFTLMTESFIEVIALLPEKDRKAIAEEIG-IDL-------N
DVPSTVSKLEKNC--N---AYADVNNIIDIQK-LDIGECTAPPGQ-HVLLQIVNTGSAEA
NCGLQTIIKSLNKVYVPPI--IENRLPYYD-------------PWFI---VGAT-IILVI
FTIAICSIR---------RNLALKYRYGTFLYV---------------------------

>gi|1070062745|ref|YP_009282739.1| SS bond formation pathway protein
substrate [Skunkpox virus]
----------------MAETREFKTLYNLFMDSYLQKLAQH-SIPTNVTCAIHIGEVVGK F-
KNCALRITNKCVSDSRLSFTLMIESFIEVVSLLPEKDRKAIAEEIG-INL-------D
DVPSTVSKLEKNC--H---AYADVNNIIDIQK-LDIGECMAPPGQ-HILLQIVNTGSAEA

NCGLQTIIKSLNKVYVPPI--IENRLPYYE-------------PWFL---IGAT-IILVI
FTVAICSIR---------RNLALKYRYGTFLYV--------------------------

>gi|1070099067|ref|YP_009281793.1| SS bond formation pathway protein substrate [Volepox virus]
----------------MAETREFKTLYNLFMDSYLQKLAQH-SIPTNVACAIHIGEVIGK F-
KNCALRITNKCVTNSRLSFTLMMESFIEVVSLLPEKDRKAIAEEIG-INL-------D
DVPSAVSKLEKNC--N---AYADVNNIIDIQK-LDIGECMAPPGQ-HILLQIVNTGSAEA
NCGLQTIIKSLNKVHVPPI--IENRLPYYE-------------PWFL---IGAT-IILVI
FTIAICSIR---------RNLALKYRYGTFLYV--------------------------

>gi|1229243048|gb|AST09440.1| SS bond formation pathway protein substrate [NY_014 poxvirus]
----------------MAETKEFKTLYNLFIDRYLQKLAQH-SIPTNISCAIHIGEIIGK F-
KNCALRITNKCMSNSKLSFTLMIESFIEVVSLLPEKDRKAIAEEIG-IDL-------N
DPPNAVSRLERNC--N---ASADVSNIVDIQE-FNVGECQAPPGQ-HILLQIINTGSAEA
NCGLQTIIKSLNRVYVPNT--IDNRLPFYK-------------PWFI---AGVV-IILVI
FSIAVCCIR---------RKLALKYRYGTFLYV--------------------------

>gi|1229242845|gb|AST09238.1| substrate for poxvirus SS bond formation pathway [Murmansk poxvirus]
----------------MAETKEFKTLYNLFIDRYLQKLAQH-SIPTNISCAIHIGEIIGK F-
KNCALRITNKCMSNSKLSFTLMIESFIEVVALLPEKDRKAIAEEIG-IDL-------N
DPPNAVSRLERNC--N---ASADVSNIVDIQE-FNVGECQAPPGQ-HILLQIINTGSAEA
NCGLQTIIKSLNKVYVPNT--IDNRLPFYK-------------PWFI---AGVV-IILVI
FAIAVCCIR---------RKLALKYRYGTFLYV--------------------------

>gi|345107212|ref|YP_004821377.1| substrate for poxvirus SS bond formation pathway [Yokapox virus]
----------------MAETKEFKTLYNLFIDKYLQKLSQH-SIPTNVSCVIHIGEILGN F-
KNCALRITNKCMKNSKLSFTLMIESFIEIASLLPEKDRKAIAEEIG-IDL-------E
DPPHFISRLEKNC--N---ATSDVSNIVDIQK-FNIGECKAPPGH-HINLQIVNTGSAEA
NCGLQTIVRSLNKIHVPNI--IDNKLPFYK-------------PWFI---AGAV-IILII
LVISICCIR---------RKMALKYRYGTFLYI--------------------------

>gi|38229187|ref|NP_938280.1| 24L [Yaba monkey tumor virus]
----------------MEEIQRVNTLYNLFVERYLQRLSSH-SVPTNISCGIHIGEVKGE L-
KRCNLRIINKCLNNPRLSFILMIESLLEVIDKLPEREKNEIANEIG-IDI-------H
SNPEIISKLERKC--N---ASSDVNNIINIQS-FDTGNCIAPENT-YILLQVINTGSAES
NCGLESILRSINKKYIQKNK-IINKPSLTE-----------K-PWFI---ICFV-IIFTV
FVIVISSLK---------RKIGFRYRYGSFLYV--------------------------

>gi|146746361|gb|ABQ43497.1| hypothetical protein [Tanapox virus]
----------------MTEIQRVNTLYDLFIEKYLQRLSLH-SVPTNINCGIHIGEVNGE L-
KRCNLRIINKCLNNPRLSFILMIESFLEVIDTLPEREKKEIADELG-IDI-------N
AGSKIISELERKC--N---ASSDVNNIINIQS-FNAGNCIAPENT-YILLQVINTGSAES
NCGLEAILKSMNKRYIQENK-IINKLPLSE-----------K-PWFI---ICVV-VIFMV
FVIAISSLR---------RKIGFRYKYGSFLYV--------------------------

>gi|12085007|ref|NP_073409.1| 24L protein [Yabalike disease virus]
----------------MTEIQRVNTLYDLFIEKYLQRLSLH-SVPTNINCGIHIGEVNGE L-
KRCNLRIINKCLNNPRLSFILMIESFLEVIDTLPEREKKEIANELG-IDI-------N
AGSKIISELERKC--N---ASSDVNNIINIQS-FNAGNCIAPENT-YILLQVINTGSAES
NCGLEAILKSMNKRYIQENK-IINKLPLSE-----------K-PWFI---ICVV-VIFMV
FVIAISSLK---------RKIGFRYKYGSFLYV--------------------------

>gi|62637409|ref|YP_227407.1| hypothetical protein DpV83gp031 [Deerpox virus W84883]
---------------MEENQVKINTLYNLFVDRYLQNLSLY-SVPVNTTCGIHIGEIRGV F-
NRCKLKIINRCLNNPKLSFVILIKTFKDIINILPQKEREELADEIG-IDLK------N

DDISYVSELERQC--N---ASADVNNIINIET-FDVGNCSAPEDK-YILLQIINSGTAEA
NCGLNAVMNALNKRYVPDPT-IYNSLPYNK-------------PWFI---IGSV-IICFI
FILGICSIK---------RKISYKYKYSPYLYV--------------------------

>gi|115503098|gb|ABI99016.1| hypothetical protein DpV84gp031 [Deerpox virus W117084]
---------------MEENQVKINTLYNLFVDRYLQNLSLY-SVPVNTTCGIHVGEIRGV F-
NRCKLRIINRCLNNPKLSFVILVKTFKDIVNILPQKEREELADEIG-IDLK------N
DDVSYVSELERRC--N---ASADVNNIINVET-FDVGNCSAPEDK-YILLQIINSGTAEA
NCGLNAVMNALNKRYVPDPT-IYNSLPFSK-------------PWFI---IGSV-IICFI
FILGICSIK---------RKISYKYKYSSYLYV--------------------------

>gi|15150463|ref|NP_150458.1| LSDV024 hypothetical protein [Lumpy skin disease virus NI2490]
----------------MENHFQVDTLYNLFIERYLQNLSLY-SVPVNTTCGIHISEIKGT L-
KGCKLKIINLCIDDKELSFYLLIKTFKEFANTLSQNEKYELANEVG-IDLD------N
QDKNYIPEIIRKC--T---SSAAVTNVIDIQT-LDIGECIAPNGK-NILLQIINSGTSEA
NCVMNTIMKSMSNRYTINNY-IDNRIPFIN-----------DVHWFI---FFVL-IIFII
FLLGLCSIK---------RKINIKYKYGSFLYV--------------------------

>gi|22595717|gb|AAN02749.1| hypothetical protein [Lumpy skin disease virus]
----------------MENHFQVDTLYNLFIERYLQNLSLY-SVPVNTTCGIHISEIKGT L-
KGCKLKIINLCIDDKELSFYLLIKTFKEFANTLSQNEKYELANEVG-IDLD------N
QDKNYIPEIIRKC--T---SSAAVTNVIDIQT-FDIGECIAPNGK-NILLQIINSGTSEA
NCVMNTIMKSMSNRYTINNY-IDNRIPFIN-----------DVHWFI---FFVL-IIFII
FLLGLCSIK---------RKINIKYKYGSFLYV--------------------------

>gi|557370484|gb|AGZ95339.1| hypothetical protein [Goatpox virus FZ]
----------------MENHFQVDTLYNLFIERYLQNLSLY-SVPVNTTCGIHISEIKGT L-
KGCKLKIINLCIDDKELSFYLLIKTFKEFANTLSQNEKYELANEVG-IDLD------N
QDKNYIPEIIRKC--T---STSAVTNVIDIQT-LDIGECIAPNGK-NILLQIINSGTSEA
NCVMNTIMKSMSNRYTINNY-IDNRIPFIN-----------DVHWFI---FFVL-IIFII
FLLGLCSIK---------RKINIKYKYGSFLYV--------------------------

>gi|13876676|gb|AAK43564.1| unknown [Lumpy skin disease virus]
----------------MENHFQVDTLYNLFIERYLQNLSLY-SVPVNTTCGIHISEIKGT L-
KGCKLKIINLCIDDKELSFYLLIKTFKEFANTLSQNEKYELANEVG-IDLD------N
QDKNYIPEIIRKC--T---SSAAVTNVIDIQT-FDIGECIAPNGK-NILLQIINFGTSEA
NCVMNTIMKSMSNRYTINNY-IDNRIPFIN-----------DVHWFI---FFVL-IIFII
FLLGLCSIK---------RKINIKYKYGSFLYV--------------------------

>gi|148912901|ref|YP_001293215.1| hypothetical protein GTPV_gp021 [Goatpox virus Pellor]
----------------MENHFQVDTLYNLFIERYLQNLSLY-SVPVNTTCGIHISEIKGT L-
KGCKLKIINLCIDDKELSFYLLIKTFKEFANTLSQNEKYELANEVG-IDLD------N
QDKNYIPEIIRKC--T---STSTVTNVIDIQT-FDIGECIAPNGK-NILLQIINSGTSEA
NCVMNTIMKSMSNRYTINNY-IDNRIPFIN-----------DVHWFI---FFVL-IIFII
FLLGLCSIK---------RKINIKYKYGSFLYV--------------------------

>gi|21492478|ref|NP_659597.1| hypothetical protein SPPV_21 [Sheeppox virus]
----------------MENHFQVDALYNLFIERYLQNLSLY-SVPVNTTCGIHISEIKGT L-
KGCKLKIINLCIDDKELSFYLLIKTFKEFANTLSQNEKYELANEVG-IDLD------N
QDKNYIPEIIRKC--T---SSAAVTNVIDIKT-FDIGECIAPNGK-NILLQIINSGTSEA
NCVMNTIMKSMSNRYTINNY-IDNRIPFIN-----------DVHWFI---FFVL-IIFII
FLLGLCSIK---------RKINIKYKYGSFLYV--------------------------

>gi|18640107|ref|NP_570181.1| SPV021 hypothetical protein [Swinepox virus]

----------------MENPVRINTLYNVFVERYIENLSIY-SIPINSTCGIHIGEIKGT F-
KRCFLKILNMCINDKELSFNILIKTLKDVTSTLSQKEKEELSKEIG-IDIL------N
NDPKYVPEIIRNC--S---SSADVTNIIDIQT-LDVGKCIAPYDK-QILLQIVNSGTAEA
NCVMNSIMNSMNRRYIDNAN-IYNYLNLTN-----------R-PWFI---FSII-IIAII
FVIGICSIK---------RRIGIKYKYGTFLYV---------------------------

>gi|377830027|ref|YP_005296228.1| unnamed protein product [Cotia virus SPAn232]
----------------MEKKMQINTLYNLFIDMYLKNLSLY-SIPVNTTCAIHIGEVSGI L-
QRCKINIVNICISNKKLNFMLLVRTFNDIVSTLPEKDKIELADEIG-IDLNN-----N
NDHNYISNLERKC--T---ASADIENIIDIQK-FNIGYCIAPEGK-TIQIQVVNSGTAEA
NCAIQTIMNSMNKRYIPDNV-INNKLKMPK-----------I-IWII---IITS-ICMIV
FVIGICLLR---------RKINFRYRYGNFLYV---------------------------

>gi|1119035593|ref|YP_009329644.1| protein F9 [BeAn 58058 virus]
----------------MEMKMQINTLYNLFINRYLQNLSLY-SIPVNTTCGIHIGEVSGV I-
ERCKINIVNVCISNKKMNFMLLVRTFNDIVNMMPEKDKIELADEIG-IDLNN-----S
NDHNYISNLERKC--T---ASADIENVIDIQK-FNIGYCKAPEGK-TIQLQVVNSGTAEA
NCAIQTIMKSMNKRYIPDNL-INNKLKIPN-----------I-TWIV---LISS-ICLII
FIIGVCLLR---------RKINFRYRYGNYLYV---------------------------

>gi|9633655|ref|NP_051733.1| M019L [Myxoma virus]
----------------MEPTAQLNTLYTLFVKRYLHNLSLY-TTPVNTTCAIHIGEIRGV F-
ERCKLQVLNFCSSNKELSFKLLVKTFREISEILPDKERDALAKEIG-IDLT------K
ADDDPNQDVVRNC--N---ASSDINNVIDIQK-FDAGYCVAPENK-HILLQIVNSGSAEA
NCVISSIVRATNKRALPDEF-VNNRLRVSN-----------R-PWFI---VFAV-FVLIV
FVICVCSIK---------RKINLKYRYGSFLYV---------------------------

>gi|539191030|gb|AGU99702.1| M019L [Myxoma virus]
----------------MEPTAQLNTLYTLFVKRYLHNLSLY-TTPVNTTCAIHIGEVRGV F-
ERCKLQILNFCSSNKELSFKLLVKTFREISEILPDKERDALAKEIG-IDLT------K
ADDDPNQDVVRNC--N---TFSDITNVIDIQK-FDAGYCVAPENK-HILLQIVNSGSAEA
NCVISSIVRATNKRALPDEF-IDNKLRVSN-----------R-PWFI---VFAV-FVLIV
FVICVCSIK---------RKINLKYRYGSFLYV---------------------------

>gi|982818174|gb|AMB18352.1| M019L [Myxoma virus]
----------------MEPTAQLNTLYTLFVKRYLHNLSLY-TTPVNTTCAIHIGEVRGV F-
ERCKLQILNFCSSNKELSFKLLVKTFREISEILPDKERDALAKEIG-IDLT------K
ADDDPNQDVVRNC--N---TFSDITNVIDIQK-FDAGYCVAPENK-HILLQIVNSGSAEA
NCVISSIVRATNKRALPDEF-VDNKLRVSN-----------R-PWFI---VFAV-FVLIV
FVICVCSIK---------RKINLKYRYGSFLYV---------------------------

>gi|9633830|ref|NP_051908.1| gp019L [Rabbit fibroma virus]
----------------MEPVAQLNTLYTLFVKRYLHNLSLY-TTPVNTTCAIHIGEIQGV F-
ERCKLQILNFCSNNKELSFKLLIKTFREISEILPDKERDALAKEIG-IDLT------K
ADDDPSQDIVRNC--N---VFSDVKNVIDIQK-FDAGYCVAPENK-HILLQIVNSGSAEA
NCVISSIVRATNKIALPDEF-INNKLRVLN-----------S-PWFI---VFAV-FVLIV
FVICVCSIK---------RKINLKYRYGSFLYV---------------------------

>gi|1215207784|gb|ASK51226.1| SS bond formation pathway protein [Eptesipox virus]
---------------MENDPIVVDTLYNLFINRYLQKLGQY-SIPTNIACGIHIGKIIGE F-
DRCALRVTNKCFNNSKLSFKIMIESLKEILLLVSEKEKIKILEEIG-IDL-------N
NDDKSISKLERNC--K---AYAETSNFIDIDV-FNAGICKAPPGK-FILLQVLNSGTVEA
NCGLETIMKSLNKKILPSYN-TNNKLDLTS-----------R-PWFI---AGGV-LILLI
IIFAICSIK---------KRISVKYNYGKYLYV---------------------------

>gi|1046611009|ref|YP_009268732.1| ss bond formation pathway protein [Pteropox virus]
----------------MAATKPVKTLYDMFADRYMELLAIY-AVPTNITCAIHIGNISGT L-

NGCALKIINRCNNNTKLSYSLLTQAFLETISKLPINEQKKIADKVG-IDL-------N
LKPGETSKLEQQC--S---ATASVTSIIDVQN-LNIGTCVSPPGQ-HILIQFINTGSASA
NCGINTILQALVNNYEI-PI-IKPTISFNN-------------PWII---ALSV-IITFI
IIISVSSLR---------RRIKLAYKYGATLRV---------------------------

>gi|571797936|ref|YP_008658435.1| envelope protein poxlipid membrane protein [Squirrelpox virus]
----------------MDNTVAEETLYDIFVSKYLQRIAEH-AAPTQTACAVHIGEIRGT M-
RGCELRVVNRCMNNARLSFSLMRDAFEETVAMLPESERRAVAAEVG-FDP-------G
APRDQPSVLERNC--S---ASAAVNDIVDIQK-LDIGECFAPPGR-HILVQIVNSGSAEA
NCGLESVARSLNKRATKELAPLHSSVPLAG-------------LWKL---GVAV-LVALI
AVVLVCAMR---------RRIALKYRYGAFVYV---------------------------

>gi|1158620706|gb|AQY16587.1| MC016 [Molluscum contagiosum virus subtype 2]
-----------------MASPALSTLYGAFVARYLRKLSLY-STTNSVTCAIHVGRIVGT L-
QNCSVRVLNRCNNNDQLSFRLLLEAFAETVSLLPPKQRAEIAAQVG-IDL-------E
AASHEESRLERKC--R---AHAALVQNLDVQT-LNVGTCIAPPGR-SLAMQVVNSGSAAA
NCGLEAIIRSLSQRPAFVPVDARRALRPGA-------------LARL---LAAA-AAVLL
ALLALAALR---------RRLRLRYQYATDLYV---------------------------

>gi|9628948|ref|NP_043967.1| MC016L [Molluscum contagiosum virus subtype 1]
-----------------MASAALSTLYGAFVARYLRKLSLY-STTNSVTCAIHVGRIVGT L-
QNCSVRILNRCNNNDQLSFRLLLEAFAETVNLLPPKQRAEIAAQVG-VDL-------E
AASHEESRLERKC--R---AHAALVQSIDVQT-LNVGTCIAPPGR-SLAIQVVNSGSAAA
NCGLEAIIRSLSQRPAFVPVDARRALCPGA-------------LARL---LAAA-AAVLL
ALLALAALR---------RRLRLRYQYATDLYV---------------------------

>gi|115531700|ref|YP_784226.1| hypothetical protein CRV036 [Nile crocodilepox virus]
--------------------MGPGPLFDLVVANYLRRLALY-AGVARSDCAIHVGEIRGR
LGGNCRVRLVNKCNSNAAVSFALLLESLEEVLDLAAPADKRAIGRSLG-VDF-------D
TFRSRTTDLERRC--R---AEADLRNDLDVQT-IHLGECDSP--V-PLEFQFVNSGSAVA
NCGLAAVFRALSARATSAP--VEARVPVGR-----------ADRWLA---AGAL-LAIAA
CLAIVALLR---------TTVTLRYRYATYLDGRIKG-----------------------

>gi|659488485|ref|YP_009046346.1| hypothetical protein fep_114 [Pigeonpox virus]
----------------MEYQQVLSNLYYLFSEKYLEKLSQH-PDTSNVRCGIHIGYLSGD A-
KNCIVSIINACNSNEQKSFQLLIESLIETIENLPEKQQKEIAGNIG-INID------D
YKAGKKTELQQHC--E---AYANLTQHIDIQH-FNIGTCYSPNDK-YTDIKVINTGSALS
NCGVEVILNKIKTNNTTVP--IDNKLSMDSF----------SIKWFV---IYIV--LCVL
ILLLLGYIY---------RTVRIKYTYGVYI-----------------------------

>gi|659488247|ref|YP_009046109.1| hypothetical protein pepv_116 [Penguinpox virus]
----------------MENQEVLSNLYYLFSEKYLEKLSQH-PDTSNVRCGIHIGYLSGD A-
KNCIVSIINACNSDEQKSFQLLIESLIETIENLPEKQQKKIAGRIG-INID------D
YKAGKKTELQQHC--E---AYANLTQHIDIQH-FNIGTCYSPNDK-YTDIKVINTGSALS
NCGVEIILNRIKTNNTTVP--IDNKLSMDSF----------SIKWFV---IYIV--LCVL
ILLLLGYIY---------RTVRIKYIYGVYI-----------------------------

>gi|9634782|ref|NP_039075.1| hypothetical protein FPV112 [Fowlpox virus]
----------------MENQQVISNLYYLFSEKYLEKLNQH-PDTSNVRCGIHIGYLSGE A-
KNCIVSIINACNSNEQKSFQLLIESLIETIENLPEKQQKEIAKSIG-INID------D
YKAGKKTELQQHC--E---AYANLTQHIDIQH-FNIGTCYSPNDK-YTDIKIINTGSALS
NCGVEIILNKIKTNNPIVP--IDNKLSMESF----------SIKWFI---IYIV--LCVL
ILLLLGYIY---------RTVRIKYTYGVYI-----------------------------

>gi|1173596604|gb|ARE67656.1| SWPV1128 [Shearwaterpox virus]
----------------MQNKQVLSNLYYLFSEKYLEKLNKY-RDTSNIRCGIHIGRLSGD A-
RNCRISIVNACNSDGEKNFELLIESVIETIENFPKNEQDIITADLG-INIE------D
FKLGKKTELQRHC--E---THAALIQHIDIQN-FNIGTCYSANGK-YTDIKVINTGSALS
NCGVEIVLNKLLKSNKSVP--IDNKLSMNNF----------SIKWLI---IYIV--FFLS
ILIILGYIY---------RTVRLKYIYGVYI-----------------------------

>gi|40556077|ref|NP_955162.1| CNPV139 conserved hypothetical protein
[Canarypox virus]
----------------MENNQVLSNLYYLFSEKYLEKLSKY-QDVSSINCGIHIGRISGY A-
KNCRISIVNACNSDGEKNFQLLLEALVETIENFPNKERNRIAADLG-VNIE------D
YKLGKKTDLQLHC--E---SYASLTQHIDIQN-FNIGTCYSPNDK-YVDIKVINTGSAIS
NCGVEIVLNKLLRSNKSVP--IDNKLSMDTF----------SIKWLV---IYIV--LILS
ILIILGYIY---------RTVRLKYIYGVYI-----------------------------

>gi|946699624|ref|YP_009177103.1| hypothetical protein [Turkeypox
virus]
----------------MENQQIISNLYYLFSENYLEKLSHY-PDTSNTRCGIHIGRISGD L-
RNCRINVINACNADGEKSFQLLLESMIETVDRLPVKEKKNILDKLG-INID------A
YTSGRKTDLQLHC--E---TYASLTQYIDIQN-ITIGSCYSPNNK-YVDIKVINTGNALS
NCGVEIILDKLLRTNFGIP--IDNKLSMDIF----------SIKWLV---LYII--LCLS
ILFILGYIY---------RTVRLKYVYGVYI-----------------------------

>gi|1215835429|ref|YP_009389413.1| vaccinia virus F9Llike protein
[Seal parapoxvirus]
MP---PPTSPI-LRQPDPTLAAPGSLYDVFLAKFLRALAAR-AAPASAACAVRVGAVRGR L-
RNCELMVLNRCHTDASDALALASEALAETLAALPQADRIAVANELG-VDP-------K APKLLP---
DPACA-S---AESTLAQNVDIQT-LNLGDCGAPDGR-RVRIALVNSGHAAA
NCALARVATALARRTPVRRE---GSLASGS-----------MPPWSV---LLSVATVTVI
GAVAVSLLK---------RALKLRFRFAASSADRTKLRV--------------------

>gi|28261211|gb|AAO31709.1| vaccinia virus F9Llike protein [Bovine
papular stomatitis virus]
MP---PPTPPT-PPQPDPVQADTGSLYDAFLARFLRALAAR-SAPASAACAVRVGAVRGR L-
RNCELVVLNRCHTDATEALVLASSALADTLASIPREDRIAVAKELG-IDP-------D KPQLTP---
DPACT-A---AEGALAQSIDLQT-LDIGDCGVPGGR-RVRIALVNSGHAAT
NCALAKVASALTKRVPARRQ---EGLAAGG-----------MPPWSV---LLSVAAVVVV
GTVAVSLLR---------RALRLRFRFATRLRV--------------------------

>gi|806824981|gb|AKC03555.1| putative membrane protein [Bovine papular
stomatitis virus]
MP---PPTLPT-PPQPDPVRADTGSLYDAFLAQFLRALAAR-AAPASAACAVRVGAVRGR L-
RNCELVVLNRCHTDATEALALASSALADTLASIPREDRIAVAKELD-IDP-------D KPQLTP---
DPACT-A---AEGALAQSIDLQT-LDIGDCGVPGGR-RVRIALVNSGHAAT
NCALAKVASALTKRVPVRRQ---EGLAAGG-----------MPPWSV---LLSVAAVVVV
GTVAVSLLR---------RALRLRFRFATRLRV--------------------------

>gi|41057566|ref|NP_958039.1| ORF131 putative membrane protein [Bovine
papular stomatitis virus]
MP---PPTLPT-PPQPDPVRADTGSLYDAFLARFLRALAAR-AAPASAACAVRVGAVRGR L-
RNCELVVLNRCHTDATEALALASSALADTLASIPREDRIAVAKELG-IDP-------D KPQLTP---
DPACT-A---AEGALAQSIDLQT-LDIGDCGVPGGR-RVRIALVNSGHAAT
NCALAKVASALTKRVPVRRQ---EGLAAGG-----------MPPWSV---LLSVAAVVVV
GTVAVSLLR---------RALRLRFRFATRLRV--------------------------

>gi|806824721|gb|AKC03297.1| putative membrane protein [Bovine papular
stomatitis virus]
MP---PPTPPT-PPQPDPVRADTGSLYDAFLARFLRALAAR-AAPASAACAVRVGAVRGR L-
RNCELVVLNRCHTDATEALALASSALADTLASIPREDRIAVAKELG-IDP-------D KPQLTP---

DPACA-A---AEGALAQSIDLQT-LDIGDCGVPGGR-RVRIALVNSGHAAT
NCALAKVASALTKRVPVRRQ---EGLAAGG-----------MPPWSV---LLSVAAVVVV
GTVAVSLLR---------RALRLRFRFATRLRV---------------------------

>gi|28261202|gb|AAO31701.1| vaccinia virus F9Llike protein [Orf virus strain D1701]
MP---PRTPPT-PHSPEPTPAAPGSLYDVFLARFLRQLAAR-AAPASAACAVRVGAVRGR L-
RNCELVVLNRCHADAAGALALASAALAETLAELPRADRLAVARELG-VDP-------E HPELTP---
DPAC--A---GESALAQNIDIQT-LDLGDCGDPKGR-RLRVALVNSGHAAA
NCALARVATALTRRVPASRH----GLAEGG-----------TPPWTL---LLAVAAVTVL
SVVAVSLLR---------RALRVRYQFARPA-ALRA------------------------

>gi|576864655|gb|AHH34314.1| putative membrane protein [Orf virus]
MP---PRTPPT-PHSPEPTPAAPGSLYDVFLARFLRRLAAR-AAPASAACAVRVGAVRGR L-
RNCELVVLNRCHADAAGALALASAALAETLAELPRADRLAVARELG-VDP-------E HPELTP---
DPAC--A---GESALAQNIDIQT-LDLGDCGDPKGR-RLRVALVNSGHAAA
NCALARVATALTRRVPASRH----GLAEGG-----------TPPWTL---LLAVAAATVL
GVVAISLLR---------RALRVRYRFARPA-ALRA------------------------

>gi|632123611|gb|AHZ33829.1| membrane protein [Orf virus]
MP---PRTPPTPPHSPEPTPAAPGSLYDVFLARFLRRLAAR-AAPASAACAVRVGAVRGR L-
RNCELVVLNRCHADAAGALALASAALAETLAELPRADRLAVARELG-VDP-------E HPELTP---
DPAC--A---GESALAQNIDIQT-LDLGDCGDPRGR-RLRVALVNSGHAAA
NCALARVATALTRRVPASRH----GLAEGG-----------TPPWTL---LLAVAAVTVL
GVVAVSLLR---------RALRVRYRFARPV-ALRA------------------------

>gi|915529|gb|AAA86391.1| similar to vaccinia virus F9L, SwissProt Accession Number P21018 [Orf virus]
MP---PRTPPTPPHSPEPTPAAPGSLYDVFLARFLRRLAAR-AAPASAACAVRVGAVRGR L-
RNCELVVLNRCHADAAGALALASAALAETLAELPRADKLAVALELG-VDP-------E HPELTP---
DPAC--A---GESALAQNIDIQT-LDLGDCGDPKGR-RLRVALVNSGHAAA
NCALARVATALTRRVPASRH----GLAEGG-----------TPPWTL---LLAVAAVTVL
GVVAVSLLR---------RALRVRYRFARPA-ALRA------------------------

>gi|41018618|gb|AAR98226.1| ORF131 putative membrane protein [Orf virus]
MP---LQTPPTPPHSPEPTPAAPGSLYDVFLARFLRRLAAR-AAPASAACAVRVGAVRGR L-
RNCELVVLNRCHADAAGALALASAALADTLAELPRADKLAVARELG-VDP-------E HPELMP---
DPAC--A---GESALAQNIDIQT-LDLGDCGDPKGR-RLRVALVNSGHAAA
NCALARVATALTRRVPASRH----GLAEGG-----------VPPWTL---LLAVAAVTVL
GVVAISLLR---------RALRVRYRFARPA-ALRA------------------------

>gi|913204007|gb|AKU76621.1| Membrane protein [Orf virus]
MP---PRTPPTPPHYPEPTPAAPGSLYDVFLARFLRRLAAR-AAPASAACAVRVGAVRGR L-
RNCELVVLNRCHADAADALALASAALAETLATLPAADRLAVARELG-VDP-------E HPELTP---
DPAC--A---GESELAQNIDIQT-LDLGDCGDPRGR-RLRVALVNSGHAAA
NCALARVAAALTRRVPASQH----GIAEGG-----------TPPWTL---LLAVAAVTVL
GVVAVSLLQ---------RALRVRYRFARPA-TLRA------------------------

>gi|913204395|gb|AKU77006.1| Membrane protein [Orf virus]
MP---PRTPPTPPHYPEPTPAAPGSLYDVFLARFLRRLAAR-AAPASAACAVRVGAVRGR L-
RNCELVVLNRCHADAADALALASAALAETLATLPAADRLAVARELG-VDP-------E HPELTP---
DPAC--A---GESALAQNIDVQT-LDLGDCGDPRGR-RLRVALVNSGHAAA
NCALARVATALTRHVPASRH----GIAEGG-----------TPPWTL---LLAVAAVTVL
GVVAVSLLR---------RALRIRFRYSKPIQTLRV------------------------

>gi|41057194|ref|NP_957908.1| ORF131 putative membrane protein [Orf virus]
MP---PRTPPTPPHYPEPTPAAPGSLYDVFLARFLRRLAAR-AAPASAACAVRVGAVRGR L-
RNCELVVLNRCHADAADALALASAALAETLAALPAADRLAVARELG-VDP-------E HPELTP---

```
DPAC--A---GESALAQNIDVQT-LDLGDCGDPRGR-RLRVALVNSGHAAA
NCALARVATALTRHVPASRH----GIAEGG-----------TPPWTL---LLAVAAVTVL
GVVAISLLR---------RALQIRFRYSKPIQTLRV------------------------
------------------
>gi|913204140|gb|AKU76753.1| Membrane protein [Orf virus]
MP---PRTLPTPPHYPEPTPAAPGSLYDVFLARFLRRLAAR-AAPASAACAVRVGAVRGR L-
RNCELVVLNRCHADEADALALASAALAETLAALPAADRLAVARELG-VDP-------E HPELTP---
DPAC--A---GESALAQNIDIQT-LDLGDCGDPRGR-RLRVALVNSGHAAA
NCALARVATALTRRVPVSQH----GLAEGG-----------TPPWTL---LLAVAAVTVL
GVVAISLLR---------RALRIRFRYSKPIQTLRV------------------------
------------------
>gi|913204266|gb|AKU76878.1| Membrane protein [Orf virus]
MP---PRTPPTPPHYPEPTPAAPGSLYDVFLARFLRRLAAR-AAPASAACAVRVGAVRGR L-
RNCELVVLNRCHADAADALALASAALAETLAELPRADRLAIARELG-VDP-------E KPELTP---
DPAC--A---GESALAQNIDIQT-LDLGDCRDPKGR-RLRVALVNSGHVAA
NCALARVATALTRRVPASRH----GLAEGG-----------TPPWTL---LLAVAAVTVL
GVVAISLLR---------RALRIRFRYSKPIQTLRV------------------------
------------------
>gi|289183766|ref|YP_003457307.1| membrane protein [Pseudocowpox
virus]
MPPRTPRTPPTPPRPPEPTPAAPGSLYDVFLARFLRRLAAR-AAPASAACAVRVGAVRGR L-
RNCELVVLNRCHTDAADSLALASAALAETLAELPRADKLAVAKELG-VDP-------E HPQLTP---
DPAC--A---GESALAQNIDVQT-LDLGDCGDPRGR-RLRVALVNSGHAAA
NCALARVATALTRRVPASRH----GLAEGG-----------TPPWTL---LLAVAAVTVL
GVVAVSLLR---------RALRIRFQYSKPLNTLRV------------------------
------------------
>gi|288804230|gb|ADC53896.1| membrane protein [Pseudocowpox virus]
MP---PRTPPTPPRPPEPTPAAPGSLYDVFLARFLRRLAAR-AAPASAACAVRVGAVRGR L-
RNCELVVLNRCHTDAADSLALASAALAETLAELPRADKLAVAKELG-VDP-------E HPQLTP---
DPAC--A---GESALAQNIDVQT-LDLGDCGDPRGR-RLRVALVNSGHAAA
NCALARVAAALTRRVPASRH----GLAEGG-----------TPPWTL---LLAVAAVTVV
GVVAISLLR---------RALRIRFQYSKPLNTLRV------------------------
------------------
>gi|738809510|ref|YP_009112870.1| putative membrane protein
[Parapoxvirus red deer/HL953]
M--------PTPPRADPAAVADPGSLYDVFLARFLRELAAR-AAPASAACAVRVGAVRGR L-
RNCELVVLNRCHTDSADALALASDALAATLSELPRADRLSVARELG-VDP-------Q KPVLAP---
DAACASA---AESALAQSIDLQT-LDLGDCGDPRGR-RVRVALVNSGHAST
NCVLARVAAALLRRMPHRPA---SSLAMGG-----------MPPWKL---LLAVAAASVV
GVIAVSLLR---------RALRLRFRFAAPMASTHATLKI--------------------
------------------
>gi|582973317|ref|YP_009001691.1| SS bond formation pathway protein
[Anomala cuprea entomopoxvirus]
------------------MDALGLSIFNDIYNRFITKVNAM-VQHTNINCNINVGSIETT
NINNCNILLSNTCISNETSTFTLLLQSIADVIRLLPEERRKIIENALG-VTVD------D
IDLNNDVGFIYSC--R---NEALLNSNINIES-IILRNCSSTT---PIDMIFSNTGSVES
NCGLRYVNEALINKNKETI--NYDLKYIFRL----------GLMEYI---IIIL-LIFFT
YIIYLMINFYIYN-----KSKSLYYSRNTVLNNDDNILTNIYIRHNNGKRNFY-------
------------------
>gi|506498247|ref|YP_008004044.1| SS bond formation pathway protein
substrate (CopF9L) [Adoxophyes honmai entomopoxvirus 'L']
-------------------MSIGITIFNDVYTRFVDKLNRI-GSTTSINCNIDIGKISTS
NINNCNIILSNKCVSNEITSFSLLLQSLGEVMLLLPEDRKNQLENLLG-ISTE------D
IVNESDTGFIQQC--R---AQAIVDNSINIGK-IEINNCYSRN---PVDFLFLNSGSAES
NCGIKFISDTLLKLNDTSP--ILSLKLLFNI----------KMFDYI---IILI-LLILL
YILYIFLSFLLPK-----TGKTIYYSRNTILNANDKILENIHLRHFEGIKDFL-------
------------------
>gi|506498553|ref|YP_008004349.1| SS bond formation pathway protein
substrate (CopF9L) [Choristoneura biennis entomopoxvirus]
-------------MNNRNNINIGRTIFNDIYTRFIDKLNRI-SNTTNINCNIDINEIRTR
```

```
NINNCNIVLSNKCVSNEITSFTLLLQSLGEVMLLLPEDRRTQIENILG-LKTE------D
IINENDIGFIHDC--T---TNAIVDNNINIAT-IEINNCYSRF---PTDFLFLNAGSADS
NCGIKYISDALLKLDERQP--ELSLQLLFNI----------KMFDYI---VILI-TIILI
YILFIFLSFLLPR-----NNKTIYYSRNTILNKNDKILENIHLRHYDGIDRLL-------
------------------
>gi|506498856|ref|YP_008004650.1| SS bond formation pathway protein
substrate (CopF9L) [Choristoneura rosaceana entomopoxvirus 'L']
-------------MNNRNNINIGRTIFNDIYTRFIDKLNRI-SNTTNINCNIFINKIITK
NINNCNIVLSNKCVSNEITSFTLLLQSLGEVMLLLPEDRRIQLENILG-LKTE------D
IINENDTGFIHDC--T---TDATVDNNINIATAIEINNCYSRF---PTDFLFLNAGSADS
NCGIKYISDALLKLDERQP--ELSLQLLFNI----------KMFDYI---VILI-TIILI
YILFIFLSFLLPR-----NNKTIYYSRNTILNKNDKILENIHLRHYDGINRLL-------
------------------
>gi|9964557|ref|NP_065025.1| hypothetical protein AMV243 [Amsacta
moorei entomopoxvirus]
-------MNESQLQTRNSSINIGRTIFNDVYTRFIDKLNRI-SSTTNINCNININEIRTS
NINNCNIVLSNKCVSNEITSFTLLLQSLGETMLLLPEDRRTQIENILG-ISTD------D
IINENDTGFINNC--R---ASAVVDNSINIGT-IEINDCYSNF---PTDFLFLNAGSADA
NCGIKYISDALLKLDNRKP--ELSLQLLFNI----------KMIDYI---IILI-TILSI
YILFIFMSFLIPR-----NKKSIYYSRNTILNKNDKILENIYLRHYDGINNFI-------
------------------
>gi|506497986|ref|YP_008003785.1| SS bond formation pathway protein
substrate (CopF9L) [Mythimna separata entomopoxvirus 'L']
-------------------MNIGRTIFNDIYTRFLDKLNRI-SASANINCGIDIGKIETS
NITNCNIILSNKCTSNEITSFTLLLQSLGEVMLLLPELRRIEIENILG-IKTE------D
IIAENDVGFIQDC--K---VQAIVDNNINIGI-IEINNCFSTR---PIDFLFLNSGSVES
NCGIKYISDALLNIDERQQ--QLTLELLFRL----------GMFDYI---LILS-VIILL
YFLFIFLSFLIPR-----NYKNMYYSRNTILNKNDKILENINLRHNI-------------
------------------
>gi|9631330|ref|NP_048165.1| ORF MSV094 putative membrane protein
(vaccinia F9L), similar to SW:P24361 [Melanoplus sanguinipes
entomopoxvirus]
------------------MDQLGLDIYNQIYERYLIKLNRN-TANASINCNIDVGLIETE
NINNCNLTFTNECVSNSSTNFTLLLESLVEVLKLLPPEQSSKIEKKLG-ITLS------E
LENGEDNGFVQEC--K---AQASVNNSISVNE-IKITNCYSTGSQ-PAEFIFVNSGTVES
NCGMSKISEALQNTNIIIEKEDEVVTNIFGV----------DLLGII---VLLV-TLILA
YIFYCIIVMHIYK-----TKSAIYFSRNTIVPEDELILHNINIRRTIPIINDR-------
------------------
>gi|51317197|gb|AAT99854.1| putative membrane protein [Diachasmimorpha
longicaudata entomopoxvirus]
-------------MDLSGFKEDDFFLYEKIMNTFFTKLYIS-SAIASGTCNITIKRLVLK
NSSTCKIDVKNVCMLNSSKTTSLLIESIKDNEKFITKDIEKKL-KRFN-I---------D
YKQWDNSELKRTC--E---AFASVENTIDIDT-FSIENCYSSTDS-IASIILLNSSDASA
NCVSKNIVKALGASDDGSVA-ELXKIERDKQ----------NNRSII---QAIL-TIFIG
IIFLVGLLVWCVD-----FKNIFVFSFKSLLKPPKRI-----------------------
------------------
>Query_247142
----------------MGAAASIQTTVNTLSERISSKLEQEANASAQTKCDIEIGNFYIR
QNHGCNLTVKNMCSADADAQLDAVLSAATETYSGLTPEQKAYVPAMFT-AALNIQTSVNT
VVRDFENYVKQTC--N---SSAVVDNKLKIQN-VIIDECYGAPGS-PTNLEFINTGSSKG
NCAIKALMQLTTKATTQIAPKQVA-----GT----------GVQFYM---IVIG-VIILA
ALFMYYAKRMLFT--STNDKIKLILANKENVHWTTYMDTFF---RTSPMVIATTDMQN--
------------------
>gi|66275885|ref|YP_232970.1| IMV membrane protein [Vaccinia virus]
----------------MGAAASIQTTVNTLSERISSKLEQEANASAQTKCDIEIGNFYIR
QNHGCNLTVKNMCSADADAQLDAVLSAATETYSGLTPEQKAYVPAMFT-AALNIQTSVNT
VVRDFENYVKQTC--N---SSAVVDNKLKIQN-VIIDECYGAPGS-PTNLEFINTGSSKG
NCAIKALMQLTTKATTQIAPKQVA-----GT----------GVQFYM---IVIG-VIILA
ALFMYYAKRMLFT--STNDKIKLILANKENVHWTTYMDTFF---RTSPMVIATTDMQN--
------------------
```

>gi|113195268|ref|YP_717398.1| IMV membrane protein [Taterapox virus]
----------------MGAAASIQTTVNTLSERISSKLEQEANASAQTKCDIEIGNFYIR
QNHGCNLTVKNMCSADADAQLDAVLSAATETYSGLTPEQKAYVPAMFT-AALNIQTSVNT
VVRDFENYVKQTC--N---SSAVVDNKLKIQN-VIIDECYGAPGS-PTNLEFINTGSSKG
NCAIKALMQLTTKATTQIAPRQVA-----GT----------GVQFYM---IVIG-VIILA
ALFMYYAKRMLFT--STNDKIKLILANKENVHWTTYMDTFF---RTSPMVIATTDMQN--

>gi|1210075651|emb|SNB56540.1| CPXV099 protein [Cowpox virus]
----------------MGAAASIQTTVNTLSERISSKLEQEANASAQTKCDIEIGNFYIR
QNHGCNLTVKNMCSADADAQLDAVLSAATETYSGLTPEQKAYVPAMFT-AALNIQTSVNT
VVRDFENYVKQTC--N---SSAVVDNKLKIQN-VIIDECYGAPGS-PTNLEFINTGSSKG
NCAIKALMQLTTKATTQIAPRQVA-----GT----------GVQFYM---IVIG-VIILA
ALFMYYAKRMLFT--STNDKIKLILANKENVHWTTYMDTFF---RTSPMVIATTDM----

>gi|1210075080|emb|SNB57671.1| CPXV099 protein [Cowpox virus]
----------------MGAAASIQTTVNTLSERISSKLEQEANASAQTKCDIEIGNFYIR
QNHGCNLTVKNMCSADADAQLDAVLSAATETYSGLTPEQKAYVPAMFT-AALNIQTSVNT
VVRDFENYVKQTC--N---SSAVVDNKLKIQN-VIIDECYGAPGS-PTNLEFINTGSSKG
NCAIKALMQLTTKATTQIAPRQVA-----GT----------GVQFYM---VVIG-VIILA
ALFMYYAKRMLFT--STNDKIKLILANKENVHWTTYMDTFF---RTSPMVIATTDMQN--

>gi|732554838|gb|AIZ72837.1| IMV membrane protein [Vaccinia virus]
----------------MGAAASIQTTVNTLSERISSKLEQEANASAQTKCDIEIGNFYIR
QNHGCNLTVKNMCSADADAQLDAVLSAATETYSGLTPEQKAYVPAIFT-AALNIQTSVNT
VVRDFENYVKQTC--N---SSAVVDNKLKIQN-VIIDECYGAPGS-PTNLEFINTGSSKG
NCAIKALMQLTTKATTQIAPRQVA-----GT----------GVQFYM---IVIG-VIILA
ALFMYYAKRMLFT--STNDKIKLILANKENVHWTTYMDTFF---RTSPMVIATTDMQN--

>gi|9627594|ref|NP_042117.1| hypothetical protein VARVgp073 [Variola
virus]
----------------MGAAASIQTTVNTLSERISSKLEQEANASAQTKCDIEIGNFYIR
QNHGCNLTVKNMCSADADAQLDAVLSAATETYSGLTPEQKAYVPAMFT-AALNIQTSVNT
VVRDFENYVKQTC--N---SSAVVDNKLKIQN-VIIDECYGAPGS-PTNLEFINTGSSKG
NCAIKALMQLTTKATTQIAPRQVA-----GT----------GVQFYM---IVIG-VIILA
ALFMYYAKRMLFT--STNDKIKLILANKENVHWTTYMDTFF---RTSPMVIATTDIQN--

>gi|90660326|gb|ABD97440.1| IMV membrane protein [Cowpox virus]
----------------MGAAASIQTTVNTLSERISSKLEQEANASAQTKCDIEIGNFYIR
QNHGCNLTVKNMCSADADAQLDAVLSAATETYSSLTPEQKAYVPAMFT-AALNIQTSVNT
VVRDFENYVKQTC--N---SSAVVDNKLKIQN-VIIDECYGAPGS-PTNLEFINTGSSKG
NCAIKALMQLTTKATTQIAPRQVA-----GT----------GVQFYM---IVIG-VIILA
ALFMYYAKRMLFT--STNDKIKLILANKENVHWTTYMDTFF---RTSPMVIATTDMQN--

>gi|325558042|gb|ADZ29423.1| IMV membrane protein [Cowpox virus]
----------------MGAAASIQTTVNTLSERISSKLEQEANASAQTKCDIEIGNFYIR
QNHGCNLTVKNMCSADADAQLDAVLSAATETYSSLTPEQKAYVPAMFT-AALNIQTSVNT
VVRDFENYVKQTC--N---SSAVVDNKLKIQN-VIIDECYGAPGS-PTNLEFINTGSSKG
NCAIKALMQLTTKATTQIAPRQVA-----GT----------GVQFYM---IVIG-VIILA
ALFMYYAKRMLFT--STNDKIKLILANKENVHWTTYMDTFF---RTSPMVIATTDIQN--

>gi|20178464|ref|NP_619885.1| CPXV099 protein [Cowpox virus]
----------------MGAAASIQTTVNTLSERISSKLEQEANASAQTKCDIEIGNFYIR
QNHGCNLTVKNMCSADADAQLDAVLSAATETYSSLTPEQKAYVPAMFT-AALNIQTSVNT
VVRDFENYVKQTC--N---SSAVVDNKLKIQN-VIIDECYGAPGS-PTNLEFINTGSSKG
NCAIKALMQLTTKATTQIAPRQVA-----GS----------GVQFYM---IVIG-VIILA
ALFMYYAKRMLFT--STNDKIKLILANKENVHWTTYMDTFF---RTSPMVIATTDIQN--

>gi|1143682|emb|CAA53878.1| unnamed protein product [Variola virus]
----------------MGAAASIQTTVNTLSERISSKLEQEANASAQTKCDIEIGNFYIR
QNHGCNLTVKNMCSADADAQLDAVLSAATETYSGLTPEQKTYVPAMFT-AALNIQTSVNT

```
VVRDFENYVKQTC--N---SSAVVDNKLKIQN-VIIDECYGAPGS-PTNLEFINTGSSKG
NCAIKALMQLTTKATTQIAPRQVA-----GT----------GVQFYM---IVIG-VIILA
ALFMYYAKRMLFT--STNDKIKLILANKENVHWTTYMDTFF---RTSPMVIATTDIQN--
------------------
>gi|29029580|gb|AAN78221.1| M1R [Monkeypox virus]
----------------MGAAASIQTTVNTLSERISSKLEQEANASAQTKCDIEIGNFYIR
QNHGCNITVKNMCSADADAQLDAVLSAATETYSGLTPEQKAYVPAMFT-AALNIQTSVNT
VVRDFENYVKQTC--N---SSAVVDNKLKIQN-VIIDECYGAPGS-PTNLEFINTGSSKG
NCAIKALMQLTTKATTQIAPRQVA-----GT----------GVQFYM---IVIG-VIILA
ALFMYYAKRMLFT--STNDKIKLILANKENVHWTTYMDTFF---RTSPMIIATTDMQN--
------------------
>gi|17974993|ref|NP_536507.1| M1R [Monkeypox virus Zaire96I16]
----------------MGAAASIQTTVNTLSERISSKLEQEANASAQTKCDIEIGNFYIR
QNHGCNITVKNMCSADADAQLDAVLSAATETYSGLTPEQKAYVPAMFT-AALNIQTSVNT
VVRDFENYVKQTC--N---SSAVVDNKLKIQN-VIIDECYGAPGS-PTNLEFINTGSSKG
NCAIKALMQLTTKATTQIAPRQVA-----GT----------GVQFYM---IVIG-VIILA
ALFMYYAKRMLFT--STNDKIKLILANKENVHWTTYMDTFF---RTSPMIIATTDIQN--
------------------
>gi|300872703|gb|ADK39105.1| myristylprotein [Monkeypox virus]
----------------MGAAASIQTTVNTLSERISSKLEQEANASAQTKCDIEIGNFYIR
QNHGCNITVKNMCSADADAQLDAVLSAATETYSGLTPEQKAYVPAMFT-AALNIQTSVNT
VVRDFENYVKQTC--N---SSAVVDNKLKIQN-VIIDECYGAPGS-PTNLEFINTGSSKG
NCAIKALMQLTTKATTQIAPRQVA-----GT----------GVQFYM---IVIG-VIILA
ALFMYYAKRILFT--STNDKIKLILANKENVHWTTYMDTFF---RTSPMIIATTDIQN--
------------------
>gi|562817396|gb|AHB35726.1| IMV membrane protein [Vaccinia virus]
----------------MGAAASIQTTVNTLSERISSKLEQEANASAQTKCDIEIGNFYIR
QNHGCNLAVKNMCSADADAQLDAVLSAATETYSGLTPEQKAYVPAMFT-AALNIQTSVNT
VVRDFENYVKQTC--N---SSAVVDNKLKIQN-VIIDECYGAPGS-PTNLEFINTGSSKG
NCAIKALMQLTTKATTQIAPRQVA-----GT----------GVQFYM---IVIG-VIILA
ALFMYYAKRMLFT--STNDKIKLILANKENVHWTTYMDTFF---RTSPMVIATTDMQN--
------------------
>gi|661921024|gb|AIE40517.1| myristylprotein [Monkeypox virus]
----------------MGAAASIQTTVNTLSERISSKLEQEANASAQTKCDIEIGNFYIR
QNHGCNITVKNMCSADADAQLDAVLSAATETYSGLTPEQKAYVPAMFT-AALNIQTSVNT
VVRDFENYVKQTC--N---SSAVVDNKLKIQN-VIIDECHGAPGS-PTNLEFINTGSSKG
NCAIKALMQLTTKATTQIAPRQVA-----GT----------GVQFYM---IVIG-VIILA
ALFMYYAKRMLFT--STNDKIKLILANKENVHWTTYMDTFF---RTSPMIIATTDIQN--
------------------
>gi|1210074512|emb|SNB53955.1| CPXV099 protein [Cowpox virus]
----------------MGAAASIQTTVNTLSERISSKLEQEANASAQTKCDIEIGNFYIR
QNHGCNLTVKNMCSADADAQLDAVLSAATETYSALTPEQKAYVPAMFT-AALNIQTSVNT
VVRDFENYVKQTC--N---SSAVVDNKLKIQN-VIMDECYGAPGS-PTNLEFINTGSSKG
NCAIKALMQLTTKATTQIAPRQVA-----GS----------GVQFYM---IVIG-VIILA
ALFMYYAKRMLFT--STNDKIKLILANKENVHWTTYMDTFF---RTSPMVIATTDMQN--
------------------
>gi|6969736|gb|AAF33948.1| TL1R [Vaccinia virus Tian Tan]
----------------MGAAASIQTTVNTLSERISSKLEQEANASAQTKCDIEIGNFYIR
QNHGCNLTVKNMCSADADAQLDAVLSAATETYSGLTPEQKAYVPAMFT-AALNIQTSVNT
VVRDFENYVKQTS--N---SSAVVDNKLKIQN-VIIDECYAAPGS-PTNLEFINTGSSKG
NCAIKALMQLTTKATTQIAPRQVA-----GT----------GVQFYM---IVIG-VIILA
ALFMYYAKRMLFT--STNDKIKLILANKENVHWTTYMDTFF---RTSPMVIATTDMQN--
------------------
>gi|22164677|ref|NP_671590.1| EVM072 [Ectromelia virus]
----------------MGAAASIQTTVNTLSERISSKLEQEANASAQTKCNIEIGNFYIR
QNHGCNLTVKNMCSADADAQLDAVLSAATETYSGLTPEQKAYVPAMFT-AALNIQTSVNT
VVRDFENYVKQTC--N---SSAVVDNKLKIQN-VIIDECYGAPGS-PTNLEFINTGSSKG
NCAIKALMQLTTKATTQIAPRQVA-----GA----------GVQFYM---IVIG-VIILA
ALFMYYAKRMLFT--STHDKIKLILANKENVHWTTYMDTFF---RTSPMVIATTDMQN--
------------------
```

```
>gi|1070062787|ref|YP_009282781.1| imv membrane protein [Skunkpox
virus]
----------------MGAAASIQTTVNTLSERISSKLEQEANASAQTKCDIEIGNFYIR
QNHGCNLSVKNMCSADADAQLDAVLSAATETYSSLTPEQKAYVPAMFT-AALNIQTSVNT
VVRDFENYVKQTC--N---SSAIVDNKLKIQN-VIIDECYGAPGS-PTNLEFINTGSSKG
NCAIKALMQLTTKATTQIAPRQVA-----GT----------GVQFYM---IVIA-VVILA
ALFMYYAKRMLFT--STNDKIKIILANKENVHWTTYMDTFF---RTSPMVIATTDIQN--
------------------
>gi|18640320|ref|NP_570476.1| CMLV086 [Camelpox virus]
----------------MGAAASIQTTVNTLSERISSKLEQEANASTQTKCDVEIGNFYIR
QNHGCNLTVKNMCSADADAQLDAVLSAATETYSGLTPEQKAYVPAMFT-AALNIQTSVNT
VVRDFENYVKQTC--N---SSAVVDNKLKIQN-VIIDECYGAPGS-TTNLEFINTGSSKG
NCAIKALMQLTTKATTQIAPRQVA-----GT----------GVQFYI---IVIG-VIILA
ALFMYYAKRMLFT--STNDKIKLILANKENVHWTTYMDTFF---RTSPMVIATTDMQN--
------------------
>gi|1070099109|ref|YP_009281835.1| imv membrane protein [Volepox
virus]
----------------MGAAASIQTTVNTLSERISSKLEQEANASAQTKCDIEIGNFYIR
QNHGCNLSVKNMCSADADAQLDAVLSAATETYSSLTPEQKAYVPAMFT-AALNIQTSVNT
VVRDFENYVKQTC--N---SSAIVDNKLKIQN-VIIDECYGAPGS-PTNLEFINTGSSKG
NCAIKALMQLTTKATTQIAPKQTT-----GA----------GVQFYM---IVIA-VVILA
ALFMYYAKRMLFT--STNDKIKIILANKENVHWTTYMDTFF---RTSPMVIATTDMQN--
------------------
>gi|831934516|ref|YP_009143396.1| IMV membrane protein [Raccoonpox
virus]
----------------MGAAASIQTTVNTLSERISSKLEQEANASAQTKCDIEIGNFYIR
QNHGCNLTVKNMCSADADAQLDAVLSAATETYSSLTPEQKAYVPAMFT-AALNIQTSVNT
VVRDFENYVRQTC--N---SSAVVDNKLKIQN-VIMDECYGAPGA-PTNLEFINTGSSKG
NCAIKALMQLTTKATTQIAPRQIA-----GT----------GVQFYI---IAIA-VVVLA
VLFMYYAKRMLFT--STNDKIKIILANKENVHWTTYMDTFF---RTSPMVIATTDMQN--
------------------
>gi|345107256|ref|YP_004821421.1| IMV membrane protein [Yokapox virus]
----------------MGAAASIQTTVNTLSERISSKLIQEANASAETKCDIDIGSFIIR
KNNGCNVTVKNLCSANADAQLEAVLSAATETYASLTPEQKAYVPAMFT-AALNIQTSVNT
VVRDFENYVRQTC--N---SSAVVDNKLKIQN-VFIDECNAIPGE-NTTLEFINAGTSKG
NCAIKSLLDVTTKATTQITPKQIA-----GT----------GIQFYI---IAIV-VIVLA
FLFIYYAKRMLFT--STNDKIKLILANKENVHWTTYMDVFF---RNNSPLVMTTEDDL--
------------------
>gi|1236513682|ref|YP_009408468.1| IMV membrane protein [NY_014
poxvirus]
----------------MGAAASIQTSVNTLSERISTKLIQEANASAETNCEIEIGSFIIR
KNNGCNVTVKNLCSANADAQLEAVLSAATETYSSLTPEQKAYVPAMFT-AALNIQTTVNT
VVRDFENYIKQTC--N---SDAVVNNKLKIQN-IFIDECTALPGS-NTTLEFINTGTSKG
NCAIKALMDITTKANTQIAPRQIA-----GT----------GVQFYI---IAIA-VVVLG
FLFIYYAKRMLFT--STNDKIKIILANKENVHWTTYMDVFF---RNNSPLVMNTDDE---
------------------
>gi|1236513479|ref|YP_009408266.1| IMV membrane protein [Murmansk
poxvirus]
----------------MGAAASIQTSVNTLSERISTKLIQEANASAETNCEIEIGSFIIR
KNNGCNVTVKNLCSANADAQLEAVLSAATETYSSLTPEQKAYVPAMFT-AALNIQTTVNT
VVRDFENYVKQTC--N---SDAVVNNKLKIQN-IFIDECTALPGS-NTTLEFINTGTSKG
NCAIKALMDITTKANTQIAPRQIA-----GT----------GVQFYI---IAIV-VVVLG
FLFIYYAKRMLFT--STNDKIKIILANKENVHWTTYMDVFF---RNSSPLVMNTDDE---
------------------
>gi|377829969|ref|YP_005296264.1| unnamed protein product [Cotia virus
SPAn232]
----------------MGVAASVQTTVNTLNEKISTSLEQTAEASATTKCDIDIGNIIFK
ENKGCNVTVKNLCSANATAQLDSVVKAATETYDSLTPEQKAYVPGLMT-AALNIQTSVNT
VIKDFENHVRQKC--T---SKAVTDNKLKIQN-ILIDECSGTPSG-PTNLEFINTGTSQG
ICAIKTLMDVTTKATSKIAPSQSA-----GS----------GFQYYI---IGFV-VIILA
```

MLFLYYAKKMLFT--STNDKIKIILANKPDVHWTSYIDTFF---SNPTTVV---------

>gi|1119035641|ref|YP_009329692.1| protein L1 [BeAn 58058 virus]
----------------MGAAASIQTTVNTLNEKISSTLEQTAEASATTKCDVDIGNIIFK
QNRGCNVTVKNLCSSNATAQLDSVVKAATETYESLTPDQKAYVPGLMT-AALNIQTSVNT
VIKDFENHVKQKC--T---SKAVTENKLKIQN-ILIDDCAGTPSG-PTNLEFINTGTSQG
ICAIKTLMDVTTKATSKIAPSQSA-----GS----------GFQYYI---IGFV-IVILA
MLFLYYAKKMFFT--STNDKIKIILANKPDVHWTSYIDTFF---SNPTTVV---------

>gi|115503135|gb|ABI99053.1| myristylated IMV envelope protein
[Deerpox virus W117084]
----------------MGAAASVQTTVNTLNEKISNKLEQTAEASAIAKCDIEIGSITFR
QNRGCNVTVKNLCSAKADAQLDAVLKAATETYDSLTPEQKAYVPGLMT-AALNIQTSVNT
VVKDFESYVKQKC--T---SKAVIDNKLKIQN-IFIDECAAPPGT-PTNFEFINSGTSQG
ICAIKTLMDVTTKASTTISPSQSS-----GY----------GYQAYV---IAAV-AVIFA
MLFLYYAKRMLFM--STQDKIKIILANKPEVHWTSYLDTFF---SNIPTIVDENNAK---

>gi|62637446|ref|YP_227444.1| Myristylated IMV envelope protein
[Deerpox virus W84883]
----------------MGAAASVQTTVNTLNEKISNKLEQTAEASAIAKCDIEIGSITFR
QNRGCNVTVKNLCSAKADAQLDAVLKAATETYDSLTPEQKAYVPGLMT-AALNIQTSVNT
VVKDFESYVKQKC--T---SKAIIDNKLKIQN-IFIDECAAPPGT-PTNFEFINSGTSQG
ICAIKTLMDVTTKASTTISPSQSS-----GY----------GYQAYV---IAAV-AVIFA
MLFLYYAKRMLFM--STQDKIKIILANKPEVHWTSYLDTFF---SNIPTIVDENNAK---

>gi|148912937|ref|YP_001293251.1| hypothetical protein GTPV_gp056
[Goatpox virus Pellor]
----------------MGAAASIQTTVNTLNEKISSKLEQTAEATAEAKCDIEIGSIVFR
QNKGCNVTVKNLCSSKAESQLDAILKAATETYDLLTPDQKAYVPGLMT-AALNIQTSVNT
VVKDFETYVKQKC--T---SKSVIDNKLKIHN-IFIDECAAPTGT-TTNFEFINSGTSQG
ICAIKTLMDVTTKASTKISPSQSS-----GY----------GYQFYI---IAAV-VVILS
MVFLYYVKKMLFT--STKDKIKIILANKPEVHWTSYLDTFF---SNTPTIIEK-------

>gi|154268989|gb|ABS72327.1| L1R [Goatpox virus]
----------------MGAAASIQTTVNTLNEKISSKLEQTAEATSEAKCDIEIGSIVFR
QNKGCNVTVKNLCSSKAESQLDAILKAATETYDLLTPDQKAYVPGLMT-AALNIQTSVNT
VVKDFETYVKQKC--T---SKSVIDNKLKIHN-IFIDECAAPTGT-TTNFEFINSGTSQG
ICAIKTLMDVTTKASTKISPSQSS-----GY----------GYQFYI---IAAV-VVILS
MVFLYYVKKMLFT--STKDKIKIILANKPEVHWTSYLDTFF---SNTPTIIEK-------

>gi|15150499|ref|NP_150494.1| LSDV060 putative myristylated IMV
envelope protein [Lumpy skin disease virus NI2490]
----------------MGAAASIQTTVNTLNEKISSKLEQTAEATAEAKCDIEIGSIVFR
QNKGCNVTVKNLCSSKAESQLDAILKAATETYDSLTPDQKAYVPGLMT-AALNIQTSVNT
VVKDFETYVKQKC--T---SKSVIDNKLKIHN-IFIDECAAPTGT-TTNFEFINSGTSQG
ICAIKTLMDVTTKASTKFSPSQSS-----GY----------GYQFYI---IAAV-VVILS
MVFLYYVKKMLFT--STKDKIKIILANKPEVHWTSYLDTFF---SNTPTIIEK-------

>gi|21492513|ref|NP_659632.1| Myristylated IMV envelope protein
[Sheeppox virus]
----------------MGAAASIQTTVNTLNEKISSNLEQTAEATAEAKCDIEIGNIVFR
QNKGCNVTVKNLCSSKAESQLDAILKAATETYDSLTPDQKAYVPGLIT-AALNIQTSVNT
VVKDFETYVKQKC--T---SKSVIDNKLKIHN-IFIDECVAPTGT-TTNFEFINSGTSQG
ICAIKTLMDVTTKASTKFSPSQSS-----GY----------GYQFYI---IAAV-VVILS
MVFLYYVKKMLFT--STKDKIKIILANKPEVHWTSYLDTFF---SNTPTIIEK-------

>gi|18640143|ref|NP_570217.1| SPV057 putative myristylated IMV
envelope protein [Swinepox virus]
----------------MGAAASIQTTVNTLNQKISNKLEQSAEASAEAKCDIEIGNITFR
QNRGCNVIVKNLCSAQANAQLDAVIKAATETYDSLTPDQKAYVPGLLT-AALNIQTSVNT

```
VVKDFETYVKQTC--T---SKSVIDNKLKIRN-IFIDDCAAPAGT-TTNFEFINSGTSQG
ICAIKTLMDVTTKASTNIAPNQSS-----GY----------GYQYYI---IAVV-IVILS
MIFLYYAKKMLFT--STKDKIKLILASKPDVHWTSYIDTFF---SNVPTVVEEPKLN---
------------------
>gi|38229222|ref|NP_938315.1| 60R [Yaba monkey tumor virus]
----------------MGAAASIQTTVNTLNEKISNTLEQSASAEAQTNCDIEIGSIVFR
QNRGCNVTVKNLCSSDADAQLEAVIKAANETYESLTPEQKAYVPGLMT-AALNIQTSVNT
VVKDFENHVKQTC--N---SKSVVDNKLKIQN-IFIDECAAPPGT-ITNFEFINSGTSKG
ICAIKTLMNVTTKASTNISPNQSS-----GY----------GYQVYV---IAAI-AIVLS
MIFLYYAKKMFFT--STKDKIKLILASKPEVHWSSYIDTFF---SDSPTVIENIN-----
------------------
>gi|12085043|ref|NP_073445.1| 60R protein [Yabalike disease virus]
----------------MGAAASIQTTVNTLNEKISNTLEQDASATAQANCDIEIGSIIFR
QNRGCNVTVKNLCSANADAQLDAVLKAASDTYESLTPEQKAYVPGLMT-AALNIQTSVNT
VVKDFENHVKQKC--N---SQSVVDNKLKIQN-IFIDECSAPSGT-TTNFEFINSGTSKG
ICAIKTIMDVTTKASTNISPKQES-----GY----------GYQVYV---IAAI-AIILS
MIFLYYAKKMFFT--STKDKIKLILASKPEVHWSSYIDTFF---SNSPTVIENIN-----
------------------
>gi|1237089682|ref|YP_009408015.1| Myristylated IMV envelope protein
[Eptesipox virus]
----------------MGAAASIQTTVNTLSEKISNKLEQTAEATASTNCDIEIGNIIFR
QNKGCNVTVKNLCSSKADAQLDAVVKAAAETFETLTPEQKAYVPSLFT-AALNIQTSVNT
VVKDFETYVKQKC--K---SDSVIKNKLKVQN-ILIDECASPPGT-TTNFEFINSGTSQG
ICAIKTLMDVTTKASTNISPKQST-----GF----------GYQYYI---IAAV-VVILA
LLFVYYTKRMFFM--STQDKIKVILASKPDVHWTSYIDTFF---SHVPTVVDVSRQN---
------------------
>gi|539191066|gb|AGU99738.1| m55R [Myxoma virus]
----------------MGAAASVQTTVNTLTQKISSSLEQTSSASAQTNCEVEIGNISFK
KNTGCNVTVKNLCSANANSQLDSVLSAATETYDSLTPEQKAYVPSLMT-AALNIQTSVNT
VVKDFENYVRQKC--T---ADSVVNNKLKVQN-IVIDECAAADGS-STNFEFINTGSSQG
ICAVKTLLDVTTKASTDISPRQSS-----GY----------GYQFYV---IAAV-VLILS
MVFMYYAKKMLFT--STKDKIKIILANKPDVHWTSYLDTFF---STTHSV----------
------------------
>gi|9633691|ref|NP_051769.1| m55R [Myxoma virus]
----------------MGAAASVQTTVNTLTQKISSSLEQTSSASAQTNCEVEIGNISFK
KNTGCNVTVKNLCSANANSQLDSVLSAATETYDSLTPEQKAYVPSLMT-AALNIQTSVNT
VVKDFENYVRQKC--T---ADSVVNNKLKVQN-IVIDECAAADGS-STNFEFINTGSSQG
ICAVKTLLDVTTKASTDISPRQSS-----GY----------GYQFYV---IAAV-VLILS
MIFMYYAKKMLFT--STKDKIKIILANKPDVHWTSYLDTFF---STVHSV----------
------------------
>gi|9633864|ref|NP_051944.1| gp055R [Rabbit fibroma virus]
----------------MGAAASVQTTVNTLTQKISSSLEQTSSASAQTNCEVEIGNISFK
KNNGCNVTVKNLCSANANSQLESVLSAATETYDSLTPEQKAYVPSLMT-AALNIQTSVNT
VVKDFENYVRQKC--T---ADSVVNNKLKVQN-IIIDECAAADGS-MTNFEFINTGSSQG
ICAVKTLLDVTTKASTDISPRQSS-----GY----------GYQFYI---IAAV-VLILS
MVFIYYAKKMLFT--STKDKIKIILANKPDVHWTSYLDTFF---STTHSI----------
------------------
>gi|571797980|ref|YP_008658479.1| myristylprotein / IMV protein
[Squirrelpox virus]
----------------MGASLSLQTTVNVVSERIRNNLETTAGASATANCNVNIGSIIFH
KNSGCNLSVRNMCSAEADAQLEAVLKAATETYEGLTTEQKAYVPGLLT-TALNIQTSVST
VVKDFETHVRQSC--N---STAVVNDNITVQN-IQVDECTAPAGS-LITMEFVNTGTSKG
NCAVKALMDVLTKSSTTMATQQAA-----GV----------GLNPYL---IAAA-VAILV
LVLLYYAKKMFFT--STQDKIKIILANKPDVHWTTYLDTFF---SNSPSMVTG-------
------------------
>gi|1158620761|gb|AQY16642.1| MC069 [Molluscum contagiosum virus
subtype 2]
----------------MGAAASVQTTVTTLNERISNKLEQTASASATANCDVSIGNIYFG
RNHGCNVLVKNMCSANADAQLDAIVKAATEVYNELSEEQKAYAPSLLT-AALNIQTNVST
VTKDFETYVKQAC--K---ADAVVNNTIKVQN-LRVDECSAPSGM-LMTFEFINTGTSVG
```

NCAMKAVLDVLTKSSDRISGVQTA-----GT----------DIRWYV---IVAA-VVACV
LLVLWYAKRMLFT--STQDKIKLILASKPDVHWTTFLDTFF---SSAPTVL---------

>gi|9629001|ref|NP_044020.1| MC069R [Molluscum contagiosum virus subtype 1]
----------------MGAAASVQTTVTTLNERISNKLEQSASASATANCNVSIGNIYFG
RNHGCNVLVKNMCSANADAQLDAIVKAATEVYNELSEEQKAYAPSLLT-AALNIQTNVST
VTKDFETYVKQAC--K---ADAVVNNTIKVQN-LRVDECSAPSGM-LMTFEFINTGTSVG
NCAMKALLDVLTKSSDRISGVQNT-----GT----------DIRWYV---IVAA-VVACV
LLVLWYAKRMLFT--STQDKIKLILASKPDVHWTTFLDTFF---SSAPTVL---------

>gi|659488260|ref|YP_009046122.1| myristylated protein [Penguinpox virus]
----------------MGAAASIQTTVTTINKKISEKLEQSASASATANCDINIGNIIFK
KNKGCNVLVKNMCSANASAQLDAIVSAVREVYDQLTEQQKAYAPSLLT-AALNIQTNVST
ITQDFETYIKQKC--N---SEAVINNTINVQS-LEVDECSAPPGQ-IMTFEFINTGTATG
NCAMKSVLDVLTKSSDRVSGNQSA-----GS----------DFAKYL---YIIG-GIICF
LILLYYAKKLFFM--STNDKVKVILAKKPDVHWTTYIDTYF---RSSPVLV---------

>gi|659488498|ref|YP_009046359.1| myristylated protein [Pigeonpox virus]
----------------MGAAASIQTTVTTINKKISEKLEQSASASATANCDINIGNIIFK
KNKGCNVLVKNMCSANASAQLDAIVSAVREVYDQLTEQQKAYAPSLLT-AALNIQTNVST
ITQDFETYIKQKC--N---SEAVVNNTINVQS-LEVNECSAPPGQ-LMTFEFINTGTATG
NCAMKSVLDVLTKSSDRVSGNQSA-----GS----------DFAKYL---YIIG-GIICF
LILLYYAKKLFFM--STNDKVKVILAKKPDVHWTTYIDTYF---RSSPVLV---------

>gi|9634798|ref|NP_039091.1| Myristylated membrane protein [Fowlpox virus]
----------------MGAAASIQTTVTTINKKISEKLEQTASASATANCDINIGNIIFK
KNKGCNVLVKNMCSANASAQLDAIVSAVREVYDQLTEQQKAYAPSLLT-AALNIQTNVST
ITQDFETYIKQKC--N---SDAVINNIINVQS-LEVDECSAPPGQ-IMTFEFINTGTATG
NCAMKSVLDVLTKSSDRVSGNQST-----GN----------DFSKYL---YIIG-GIICF
LILLYYAKKLFFM--STNDKVKVLLAKKPDVHWTTYIDTYF---RSSPVLV---------

>gi|221404|dbj|BAA00225.1| unnamed protein product [Fowlpox virus]
----------------MGAAASIQTTVTTINKKICEKLEQTASASRTANCDINIGNIIFK
KNKGCNVLVKNMCSANASAQLDAIVSAVREVYDQLTEQQKAYAPSLLT-AALNIQTNVST
ITQDFETYIKQKC--N---SDAVINNIINVQS-LEVDECSAPPGQ-IMTFEFINTGTATG
NCAMKSVLDVLTKSSDRVSGNQST-----GN----------DFSKYL---YIIG-GIICF
LILLYYAKKLFFM--STNDKVKVLLAKKPDVHWTTYIDTYF---RSSPVLV---------

>gi|1173596344|gb|ARE67397.1| SWPV2ORF161 [Shearwaterpox virus]
----------------MGAAASIQTTVTTLNKRISEKLEQSASASATANCDIHIGNILFK
KNRGCNVLVKNMCSANASAQLDAIVSAVKEVYNDLTSEQKAYAPSLLT-AALNIQTNVST
ITQDFETYLKQKC--N---SDAVIKNTLSIQS-LEVDDCSAPPGQ-IMTFEFINTGTATG
NCAMKSVLDVLTKSSDRVSGNQES-----GN----------DFVKYL---YIIG-GVICF
LILLYYIKKLFFM--STNDKVKVILAKKPDVHWSTYLDTYF---RSSPVII---------

>gi|40556111|ref|NP_955196.1| CNPV173 putative myristylated IMV envelope protein [Canarypox virus]
----------------MGAAASIQTTVTTLNKRISEKLEQSAQASAAANCDIHIGNILFK
KNRGCNVLVKNMCSANASAQLDAIVSAVKEVYNDLSAEQKAYAPSLLT-AALNIQTNVST
ITQDFETYLKQKC--N---SDAVIKNTLSIQS-LEVDDCSAPPGQ-IMTFEFINTGTATG
NCAMKSVLDVLTKSSDRVSGNQES-----GN----------DFVKYL---YIIG-GVICF
LILLYYIKKLFFM--STNDKVKVILAKKPDVHWSTYLDTYF---RSSPVII---------

>gi|1174036708|gb|ARF02733.1| SWPV1149 [Shearwaterpox virus]
----------------MGAAASIQTTVTTLNKKISDKLEQTAQASASTNCDIHIGNILFK
KNRGCNVLVKNMCSANSAAQLDAIVSAVKDVYNDLSSEQKAYAPSLLT-AALNIQTNVST

ITQDFETYLKQKC--N---SDSVVKNTLSIQS-LEVDECSAPPGQ-IMTFEFINTGTATG
NCAMKSVLDVLTKSSDRVSGNQES-----GN----------DFAKYL---YIIG-AVICF
LVLLYYVKKLFFM--STNDKVKVILAKKPDVHWTTFLDTYF---RSSPILV---------

>gi|946699633|ref|YP_009177112.1| myristylated membrane protein [Turkeypox virus]
----------------MGAAASVQTTVTTINKKISEKLEQTASATATANCDINIGNIVFK
KNRGCNVLVKNMCSANSSAQLDAIVSAVKEVFNDLNETQKSYAPSLLT-AALNIQTNVST
VVEDFETYIRQKC--T---SSSVINNNINIQS-LQVDDCSAPPGQ-IMTFEFINTGTSTG
NCAMKSVLDVLTKSSDRVSGNQES-----SN----------DFVKYL---YIIG-GVICF
LLLLYYIKKLFFM--SVQDKVKIILAKKPDVHWTTFLDTYF---RTSPVLV---------

>gi|1046611057|ref|YP_009268780.1| imv membrane protein [Pteropox virus]
----------------MGASVSVQTVVNTINDKIRTKLEQQASASATAVCDVTIGSLIIR
KNLGCSVSVRNLCSAQADAQLDAILSAVTDVYNNLSDSQKAYVPELLT-ASLNIQTTVNT
AVKDFETYLKQTC--N---SDAIIHNKIKVQN-VVMDECASPPGA-ITHIDFTNTGTAKG
NCGVKAVLDVLTKASTKESSSQIA-----NI----------PTYYLI---IAIV-LVIFA
MIFLYYAKHMLVT--STTDKIKLILASKPDVHWTTYLDTFY---TYSPSVLSNKQV----

>gi|288804151|gb|ADC53817.1| IMV protein [Pseudocowpox virus]
----------------MGAAASIQTTVTTVSERIRNELEQSASASATADCDVTIGSLIIR
KNLGCSVSVRNMCSANASAQLDAVMKAVSSTFNDLSADQKAYVPGLLT-AALNIQTTVNT
AVKDFENYVKQTC--N---ADAIIHNKIKVQN-ILIDECASPAGGPATHLEFVNTGTAVG
NCGVQAVMDVLSKASTTVKSNQEA-----NK----------GYQTII---VAIV-VAILA
AIFAWYARHMLFM--STTDKIKLELAKKPVVHWTTYLDTFF---TEFPPSV---------

>gi|289183811|ref|YP_003457352.1| IMV protein [Pseudocowpox virus]
----------------MGAAASIQTTVTTVSERIRNELEQSASASATADCDVTIGSLIIR
KNLGCSVSVRNMCSANASAQLDAVMKAVSSTFNDLSADQKAYVPGLLT-AALNIQTTVNT
AVKDFENYVKQTC--N---ADAIIHNKIKVQN-ILIDECASPAGGPATHLEFVNTGTAVG
NCGVQAVMDVLSKASTTVKSNQEA-----GK----------GYQTII---IAIV-VAILA
AIFAWYARHMLFM--STTDKIKLELAKKPVVHWTTYLDTFF---TEFPPSV---------

>gi|913203922|gb|AKU76536.1| Myristylated IMV envelope protein [Orf virus]
----------------MGAAASIQTTVTTVSERIRNELEQSASASATADCDVTIGSLIIR
KNLGCSVSVRNMCSANAGAQLDAVMKAVSSTFNDLSSDQKAYVPGLLT-AALNIQTTVNT
AVKDFETYVKQTC--T---ADAVIHNKIKIQN-IVMEECASLPGSPATHLEFVNTGTAAG
NCGVKAVMDVLAKASTTVHDNQEA-----GK----------GYQTII---IAIV-VAILA
AIFAWYARHMLFM--STSDKIKLELAKKPVVHWTTYLDTFF---TEFPPSV---------

>gi|41057110|ref|NP_957824.1| ORF047 putative myristylated IMV envelope protein [Orf virus]
----------------MGAAASIQTTVTTVSERIRNELEQSASASATADCDVTIGSLIIR
KNLGCSVSVRNMCSANAGAQLDAVMKAVSSTFNDLSSDQKAYVPGLLT-AALNIQTTVNT
AVKDFETYVKQTC--T---ADAVIHNKIKIQN-IVMEECASLPGSPATHLEFVNTGTAAG
NCGVKAVMDVLAKASTTVRNNQEA-----GK----------GYQTII---IAIV-VAILA
AIFAWYARHMLFM--STSDKIKLELAKKPVVHWTTYLDTFF---TEFPPSV---------

>gi|632123526|gb|AHZ33744.1| myristylated IMV envelope protein [Orf virus]
----------------MGAAASIQTTVTTVSERIRNELEQSASASATADCDVTIGSLIIR
KNLGCSVSVRNMCSANAGAQLDAVMKAVSSTFNDLSSDQKAYVPGLLT-AALNIQTTVNT
AVKDFETYMKQTC--T---ADAVIHNKIKIQN-IVMEECASLPGSPATHLEFVNTGTAAG
NCGVKAVMDVLAKASTTVRNDQEA-----GK----------GYQTII---IAIV-VAILA
AIFAWYARHMLFM--STSDKIKLELAKKPVVHWTTYLDTFF---TEFPPSV---------

>gi|41018534|gb|AAR98142.1| ORF047 putative myristylated IMV envelope protein [Orf virus]

```
----------------MGAAASIQTTVTTVSERIRNELEQSASASATADCDVTIGSLIIR
KNLGCSVSVRNMCSANAGAQLDAVMKAVSSTFNDLSSDQKAYVPGLLT-AALNIQTTVNT
AVKDFETYMKQTC--T---ADAVIHNKIKIQN-IVMEECASLPGSPATHLEFVNTGTAVG
NCGVKAVMDVLAKASTTVRNDQEA-----GK----------GYQTII---IAIV-VAILA
AIFAWYARHMLFM--STSDKIKLELAKKPVVHWTTYLDTFF---TEFPPSV---------
------------------
>gi|74230759|gb|ABA00564.1| IMV protein [Orf virus]
----------------MGAAASIQTTVTTVSERIRNELEQSASASATADCDVTIGSLIIR
KNLGCSVSVRNMCSANAGAQLDAVMKAVSSTFNDLSSDQKAYVPGLLT-AALNIQTTVNT
AVKDFETYMKQTC--T---ADAVVHNKIKIQN-IVMEECASLPGSPATHLDFVNTGTAVG
NCGVKAVMDVLAKASTTVRNDQEA-----GK----------GYQTII---IAIV-VAILA
AIFAWYARHMLFM--STSDKIKLELAKKPVVHWTTYLDTFF---TEFPPSV---------
------------------
>gi|325073842|gb|ADY76895.1| PP188 [Orf virus]
----------------MGAAASIQTTVTTVSERIRNELEQSASASATADCDVTIGSLIIR
KNLGCSVSVRNMCSANAGAQLDAVMKAVSSTFNDLSSDQKAYVPGLLT-AALNIQTTVNT
AVKDFETYMKQTC--T---ADAVIHNKIKIQN-IVMEECASLPGSPATHLEFVNTGTAAG
KCGVKAVMEVLAKASTTVRNDQEA-----GK----------GYQTII---IAIV-VAILA
AIFAWYARHMLFM--STSDKIKLELAKKPVVHWTTYLDTFF---TEFPPSV---------
------------------
>gi|738809426|ref|YP_009112786.1| putative myristylated IMV envelope
protein [Parapoxvirus red deer/HL953]
----------------MGAAASIQTTVTTVSERIRNELEQTASASAKADCDVTIGSLIIR
KNLGCSVSVRNMCSANSSAQLDAVMKAVSSTFNDLSAEQKAYVPGLMT-AALNIQTTVTT
AVKDFETYVKQTC--N---ADSVIHNKIKVQN-IVMDECASPPGGQTTHLEFVNTGTASG
NCGVKAVMDVLAKASTTVKAEQEA-----NK----------GFNTIV---IAVV-VAILA
AVFAWYARHMFFM--STTDKIKLELAKKPVVHWTTYLDTFF---TEFPPSD---------
------------------
>gi|41057483|ref|NP_957956.1| ORF047 myristylated IMV envelope protein
[Bovine papular stomatitis virus]
----------------MGAAASIQTTVTTINERIRNELQQTASASATADCDVTIGSLIIR
KNLGCSVSVQNMCSADASAQLDAVMKAVSSTFNDLSADQKAYVPGLLT-AALNIQTTVTT
AVKDFENYVRQTC--T---ADSVIHNKIKVQS-IVMDECASPPGGQTTHLEFINTGTASG
NCGVKAVMDVLAKASTTVKDQQVA-----GK----------GYQTII---IAVV-VAILA
AVFAWYARRMLFT--STTDKIKLELAKKPVVHWTTYLDTFF---TEFPPSV---------
------------------
>gi|806824899|gb|AKC03473.1| myristylated IMV envelope protein [Bovine
papular stomatitis virus]
----------------MGAAASIQTTVTTINERIRNELQQTASASATADCDVTIGSLIIR
KNLGCSVSVQNMCSADASAQLDAVMKAVSSTFNDLSADQKAYVPGLLT-AALNIQTTVTT
AVKDFENYVRQTC--T---ADSVIHNKIKVQS-IVMDECASPPGGQTTHLEFINTGTASG
NCGVKAVMDVLAKASTTVKDQQVA-----GK----------GYQTII---IAVV-VAILA
AVFAWYARRMLFT--STADKIKLELAKKPVVHWTTYLDTFF---TEFPPSV---------
------------------
>gi|806824640|gb|AKC03216.1| myristylated IMV envelope protein [Bovine
papular stomatitis virus]
----------------MGAAASIQTTVTTINERIRNELQQTASASATADCDVTIGSLIIR
KNLGCSVSVQNMCSADASAQLDAVMKAVSSTFNDLSADQKAYVPGLLT-AALNIQTTVTT
AVKDFENYVRQTC--T---ADSVIHNKIKVQS-IVMDECASPPGGQTTHLEFINTGTASG
NCGVKAVMDVLAKASTTVKNEQVA-----GK----------GYQAIV---IAVV-VAILA
AIFAWYARRMLFT--STADKIKLELAKKPVVHWTTYLDTFF---TEFPPSV---------
------------------
>gi|1215835349|ref|YP_009389333.1| myristylated IMV envelope
proteinlike protein [Seal parapoxvirus]
----------------MGAAASIQTTVTTISERIRNELEQSASASATADCSVTIGSLIIR
KNTGCSVSVRNMCSANADAQLDAVMKAVSTTFNDLSADQKAYVPGLLT-AALNIQTTVTT
AVKDFETYVKQTC--K---SDSVVHNKIKVQN-IVMDECSSPPGGGTTHLEFVNTGTAVG
NCGVKAVMDVVAKASTSVRNEQVA-----NK----------GYQTIV---IAAV-VAILA
AVFAWYAKKMLFT--STRDKIRLALANKPVVHWTTYLDTFF---TELPSSV---------
------------------
```

```
>gi|115531746|ref|YP_784272.1| myristylated IMV envelope protein [Nile
crocodilepox virus]
----------------MGAAASLQTTVNNINQKISNKLVQTASASATANCKVNIGSLRFH
TNKGCAVNIRNLCSADAKASVQSILDAVSESYDGLTSAQKAFAPSLLT-AALNIQTDVNN
VVKDFETYVKQTC--L---ADSVLNSTITVND-IDMGDCQAPQGT-IMNFEFVNAGTASA
NCAMKAVLDVLTKSSDKIASDQVS-----NN----------DFRYYIYGAIAIA-AIVGL
FLTLYYVKKMFVM--STDDKIKLSLANREVPHWLTLLDTYLLGRRDTPHLLYE-------
------------------
>gi|9964531|ref|NP_064999.1| putative myristylated membrane protein
[Amsacta moorei entomopoxvirus]
----------------MGASASINTIVSDITNRVENSLIQTANASAQAICRVTIGSISFR
STQGCTIEVRNLCSAQAVAQVDAVVNATIDFYNNLTFEQKQEAPTWFT-VAYGINTTVTT
IENDFRNLVEQRC--K---SQAVLDSSITVDN-ILVNDCRAPGNE-IVRFTFVNSGTAAG
QCAISALLDLQVAGSNQVSASQSQ-----GL----------NIGNII---LYVA-IAIIV
IAISYVLIKFFGNKPTIKQQISLELAKNGAVSSQLIQLSRY---VSKIDDRD--------
------------------
>gi|506498821|ref|YP_008004615.1| IMV membrane protein (CopL1R)
[Choristoneura rosaceana entomopoxvirus 'L']
----------------MGVSASINTIVTDITNRVENSLIQSANASAQSVCRVSIGSISFQ
STQGCTVEIRNLCSAEAISQVDAVVDATIEFYNDLTFEQKQEAPKWFT-SAYGINTTVTT
IENDFRNLIDQRC--K---SEALLESTIEVQN-ILVKDCRAPGNE-IVRFIFTNSGTASG
QCAISALLDLQVSGANEVSASQSQ-----GT----------DISAIL---LYVI-IGVVV
VAVAYVIVKFFSNRPSPKQQINLELARHGAVSSKLIQLSQY---ISKVGHSD--------
------------------
>gi|506498520|ref|YP_008004316.1| IMV membrane protein (CopL1R)
[Choristoneura biennis entomopoxvirus]
----------------MGVSASINTIVTDITNRVENSLIQSANASAQAVCRVSIGSISFQ
STQGCTVEIRNLCSAEAISQVDAVVDATIEFYNDLTFEQKQEAPKWFT-SAYGINTTVTT
IENDFRNLIDQRC--K---SEALLESTIEVQN-ILVKDCRAPGNE-IVRFIFTNSGTASG
QCAISALLDLQVSGANEVSASQSQ-----GT----------DISAIL---LYVI-IGVVV
VAVAYVIVKFFSNRPSPKQQINLELARHGAVSSKLIQLSQY---ISKVGHSD--------
------------------
>gi|506498161|ref|YP_008003958.1| IMV membrane protein (CopL1R)
[Adoxophyes honmai entomopoxvirus 'L']
----------------MGVSASVNTIVSDITQRVENSLIQTAEASATANCRVSIGSISFT
STRGCTISVQNLCSAQAISQVDAVVEATIEFYNDLSFEQKQEAPSWFT-AAYGVNTTVST
IESDFRNLIEQRC--K---SEALLNSTIEVQN-FIVKDCTAPGNQ-IVNFTFINSGTAAG
QCAISALIDLQVSGSNTVSASQSQ-----GL----------DLGAII---IYVV-IAIIV
IAVAYVIVKFFSNKPTPNQQINLELAKMGAVSSKLIQLSKY---ISKME-----------
------------------
>gi|506497958|ref|YP_008003757.1| IMV membrane protein (CopL1R)
[Mythimna separata entomopoxvirus 'L']
----------------MGASASINTIVSDITNRVENRLEQTAEASATAICRVNIGSISFQ
STQGCVVSVSNLCSAQAVAQVDAVVNATIEFYNDLTFEQKQEAPTWFT-LAYGINTTVTT
IENDFKNLIEQRC--K---SDALLESEITVQN-ILVKDCRAPRDQ-IVNFTFTNTGTASG
QCAISALLDLQVSGSNQVSASQSQ-----GL----------DLSTII---IYVI-IAVIV
IAVAYVIVKFASTKLTPQQQVDLALAKVNSLSSRLMSYSNL---ISKTRY----------
------------------
>gi|582973196|ref|YP_009001570.1| putative myristylated membrane
protein [Anomala cuprea entomopoxvirus]
----------------MGASASINTIVSTITNRVENSLIQEANASAAAHCNVTIGSILFE
STEGCVIEVRNLCSAQAVAQVDAVVNATIDFYNDLSFEQKQEAPTWFT-AAFGINTTVSN
VTNDFRNLIEQRC--K---SDAELNNTIEVRN-ITVRSCRAPPNEGVITFSFINSGTAAG
QCAISALVDLQVAGSNTVSAKQSQ-----GF----------DLAGII---PYIV-AGIVI
VLVVYIIIKLIGMRLTPKQQIDLELAKHNATSSKLIQLSQF---ISNPNI----------
------------------
>gi|9631378|ref|NP_048254.1| ORF MSV183 putative myristylated membrane
protein, Molluscum contagiosum virus MC069R (vaccinia L1R) homolog,
similar to GB:U60315 [Melanoplus sanguinipes entomopoxvirus]
----------------MGASASVNTIVSNINNRVENSLTQEASASATANCNVEIGSITFK
```

STKGCVVEVSNLCSAQAEASVDAVVNATIDFYNDLSFEQKQEAPAWFT-AAFGVNTTTTN
ITNDFKNIVEQRC--K---ADAVLNSSITVNN-ITVADCVAPESEGVIKFTFTNSGTAAG
QCAISALLDLQVAGSNTVSAKQTT-----GT----------DWTVIF---AYVA-LVAGI
AIIAGIFYYIYKLRLSPKDKVNIELAKLGATSSKIIQLSQY---LRG-------------

>gi|918014487|ref|YP_009162469.1| myristylated IMV envelope protein L1R [Salmon gill poxvirus]
----------------MGVNVDVKEFMQKFESNLSNNLSQSGSSQAVANCKVNIGSIIFQ
SNVNCSVNITNNCVASADVSVSAVINALSDAFNSMSSTQKTSIAGFTGGINVGVSSTAQT
FIQNMTNTINQSC--S---ASAVANSEFNLGT-LSFGSCKNYFG--TTTINFINTGQARG
NCAMKLVSDLQLQSNNSNTGVQDSVFSGFGSILTAFGNLLGQYAKFI---IGFI-AILLF
TPIIYLIVKLFKK-----NKPAIGDNNSAVIFESPFIKELAFKSLNNEPLHWSLYSKFL-

>gi|918014482|ref|YP_009162464.1| myristylated IMV envelope protein F9L [Salmon gill poxvirus]
----------------MTLEFHWKKWYNEIVTDVLYDLRNY--SGYNVNCNITINNLNLT G-
NNCRVSLINVCTANAELFDKVFRSEMAKRKNYLPLYLQKQFDKIIS------------ -----
PDNINKLC--SGKLTTELVDSNVLLDS-VDI-HCDSNRNSYTEPLKIVNTGSANT
DCKYLQYATLFNKTGQPSDFFSVD-----TS----------RKNFII---YTLV-LGVLG
FGIFAGILMYLNS-----KNLKIDNYRTNVVYDPVSSEKIFKKFKVLHIAKEAKYFDKEH
KIAFGLIDNGLITTGKHV

Supplemental file 2

A: F9_L1_conserved

(((Query_119423 :0.0000,
gi_66275845_ref_YP_232930_1__SS_bond_formation_pathway_protein__Vaccinia_virus
:0.0000):0.0000[42],
gi_325558002_gb_ADZ29383_1__SS_bond_formation_pathway_protein__Cowpox_virus
:0.0000):0.0000[23],
gi_325514063_gb_ADZ24057_1__SS_bond_formation_pathway_protein__Cowpox_virus
:0.0000):0.0000[26], gi_111184232_gb_ABH08152_1__HSPV049__Horsepox_virus
:0.0000):0.0000[21], gi_554571866_gb_AGY97569_1__CPXV056_protein__Cowpox_virus
:0.0000):0.0000[24],
gi_38348914_gb_AAR17890_1__SS_bond_formation_pathway_protein__Vaccinia_virus
:0.0000):0.0000[11], gi_17974954_ref_NP_536468_1__C15L__Monkeypox_virus_Zaire96I16
:0.0000):0.0000[13], gi_44971400_gb_AAS49750_1__RPXV037__Rabbitpox_virus
:0.0000):0.0000[12], gi_20178423_ref_NP_619844_1__CPXV056_protein__Cowpox_virus
:0.0000):0.0000[12], gi_90660286_gb_ABD97400_1__unknown__Cowpox_virus
:0.0000):0.0000[12], gi_18640278_ref_NP_570434_1__CMLV044__Camelpox_virus
:0.0000):0.0000[7],
gi_113195226_ref_YP_717356_1__hypothetical_protein_TATV_DAH68_049__Taterapox_virus
:0.0000):0.0009[9], gi_137849_sp_P24361_1_F9_VACCW_RecName__Full=Protein_F9
:0.0099):0.0007[11],(gi_22164637_ref_NP_671550_1__EVM032__Ectromelia_virus
:0.0083,(((
gi_9627554_ref_NP_042077_1__SS_bond_formation_pathway_protein__Variola_virus :-
0.0052,
gi_544838_gb_AAB29629_1__C13L_product__variola_virus_VAR__India1967__Peptide__212_aa
:0.0162):0.0026[47],
gi_94487114_gb_ABF26214_1__hypothetical_protein_VARV_KUW67_1629_036__Variola_virus
:-0.0026):0.0013[74],
gi_94489329_gb_ABF28418_1__hypothetical_protein_VARV_UNK44_harv_036__Variola_virus
:-0.0013):0.0135[84]):0.0048[42]):0.0054[33],(((
gi_325558218_gb_ADZ29598_1__SS_bond_formation_pathway_protein__Cowpox_virus :-
0.0028,
gi_831934475_ref_YP_009143355_1__SS_bond_formation_pathway_protein__Raccoonpox_virus
:0.0353):0.0014[19], gi_1210076734_emb_SNB49914_1__CPXV056_protein__Cowpox_virus :-
0.0014):0.0037[15],
gi_1169132833_gb_ARB50282_1__SS_bond_formation_pathway_protein__Cowpox_virus
:0.0071):0.0042[17]):0.0208[63],(
gi_1070062745_ref_YP_009282739_1__SS_bond_formation_pathway_protein_substrate__Skunk
pox_virus :0.0223,
gi_1070099067_ref_YP_009281793_1__SS_bond_formation_pathway_protein_substrate__Volep
ox_virus :0.0214):0.0205[75]):0.0289[74],((
gi_1229243048_gb_AST09440_1__SS_bond_formation_pathway_protein_substrate__NY_014_pox
virus :-0.0029,
gi_1229242845_gb_AST09238_1__substrate_for_poxvirus_SS_bond_formation_pathway__Murma
nsk_poxvirus :0.0137):0.0050[66],
gi_345107212_ref_YP_004821377_1__substrate_for_poxvirus_SS_bond_formation_pathway__Y
okapox_virus :0.0852):0.0320[65]):0.0752[81],
gi_1215207784_gb_ASK51226_1__SS_bond_formation_pathway_protein__Eptesipox_virus
:0.3043):0.0599[54],(gi_38229187_ref_NP_938280_1__24L__Yaba_monkey_tumor_virus
:0.0420,(gi_146746361_gb_ABQ43497_1__hypothetical_protein__Tanapox_virus :0.0000,
gi_12085007_ref_NP_073409_1__24L_protein__Yabalike_disease_virus
:0.0000):0.0485[98]):0.1567[99]):0.0359[44],
gi_571797936_ref_YP_008658435_1__envelope_protein_poxlipid_membrane_protein__Squirre
lpox_virus :0.3216):0.0756[31],(((
gi_62637409_ref_YP_227407_1__hypothetical_protein_DpV83gp031__Deerpox_virus_W84883
:0.0141,
gi_115503098_gb_ABI99016_1__hypothetical_protein_DpV84gp031__Deerpox_virus_W117084

:0.0523):0.1875[99],((((
gi_15150463_ref_NP_150458_1__LSDV024_hypothetical_protein__Lumpy_skin_disease_virus_
NI2490 :-0.0042,((
gi_557370484_gb_AGZ95339_1__hypothetical_protein__Goatpox_virus_FZ :-0.0004,
gi_13876676_gb_AAK43564_1__unknown__Lumpy_skin_disease_virus :0.0221):0.0020[29],
gi_148912901_ref_YP_001293215_1__hypothetical_protein_GTPV_gp021__Goatpox_virus_Pell
or :0.0089):0.0095[43]):0.0021[43],
gi_22595717_gb_AAN02749_1__hypothetical_protein__Lumpy_skin_disease_virus :-
0.0021):0.0011[74],
gi_21492478_ref_NP_659597_1__hypothetical_protein_SPPV_21__Sheeppox_virus :-
0.0011):0.1587[100],
gi_18640107_ref_NP_570181_1__SPV021_hypothetical_protein__Swinepox_virus
:0.1251):0.0571[69]):0.0177[16],((
gi_377830027_ref_YP_005296228_1__unnamed_protein_product__Cotia_virus_SPAn232
:0.0680, gi_1119035593_ref_YP_009329644_1__protein_F9__BeAn_58058_virus
:0.0655):0.1845[99],(gi_9633655_ref_NP_051733_1__M019L__Myxoma_virus :-0.0001,((
gi_539191030_gb_AGU99702_1__M019L__Myxoma_virus :0.0000,
gi_982818174_gb_AMB18352_1__M019L__Myxoma_virus :0.0000):0.0234[91],
gi_9633830_ref_NP_051908_1__gp019L__Rabbit_fibroma_virus
:0.0314):0.0163[69]):0.1994[99]):0.0300[28]):0.0360[38]):0.0736[41],((
gi_1158620706_gb_AQY16587_1__MC016__Molluscum_contagiosum_virus_subtype_2 :0.0266,
gi_9628948_ref_NP_043967_1__MC016L__Molluscum_contagiosum_virus_subtype_1
:0.0288):0.2402[100],
gi_115531700_ref_YP_784226_1__hypothetical_protein_CRV036__Nile_crocodilepox_virus
:0.3511):0.1166[51]):0.0531[16],
gi_1046611009_ref_YP_009268732_1__ss_bond_formation_pathway_protein__Pteropox_virus
:0.3249):0.0540[25],((((
gi_659488485_ref_YP_009046346_1__hypothetical_protein_fep_114__Pigeonpox_virus :-
0.0020,
gi_659488247_ref_YP_009046109_1__hypothetical_protein_pepv_116__Penguinpox_virus
:0.0347):0.0075[60],
gi_9634782_ref_NP_039075_1__hypothetical_protein_FPV112__Fowlpox_virus
:0.0033):0.0939[96],(gi_1173596604_gb_ARE67656_1__SWPV1128__Shearwaterpox_virus
:0.0653,
gi_40556077_ref_NP_955162_1__CNPV139_conserved_hypothetical_protein__Canarypox_virus
:0.0518):0.0638[74]):0.0152[47],
gi_946699624_ref_YP_009177103_1__hypothetical_protein__Turkeypox_virus
:0.1378):0.3210[100]):0.0929[35],((
gi_1215835429_ref_YP_009389413_1__vaccinia_virus_F9Llike_protein__Seal_parapoxvirus
:0.0629,((((
gi_28261211_gb_AAO31709_1__vaccinia_virus_F9Llike_protein__Bovine_papular_stomatitis
_virus :0.0031,
gi_806824981_gb_AKC03555_1__putative_membrane_protein__Bovine_papular_stomatitis_vir
us :0.0184):0.0045[84],
gi_41057566_ref_NP_958039_1__ORF131_putative_membrane_protein__Bovine_papular_stomat
itis_virus :-0.0045):0.0023[83],
gi_806824721_gb_AKC03297_1__putative_membrane_protein__Bovine_papular_stomatitis_vir
us :-0.0023):0.1008[100],
gi_738809510_ref_YP_009112870_1__putative_membrane_protein__Parapoxvirus_red_deer/HL
953 :0.1170):0.0532[66]):0.0298[41],((((
gi_28261202_gb_AAO31701_1__vaccinia_virus_F9Llike_protein__Orf_virus_strain_D1701
:0.0172,((gi_576864655_gb_AHH34314_1__putative_membrane_protein__Orf_virus :-
0.0024, gi_41018618_gb_AAR98226_1__ORF131_putative_membrane_protein__Orf_virus
:0.0131):0.0012[42],
gi_915529_gb_AAA86391_1__similar_to_vaccinia_virus_F9L__SwissProt_Accession_Number_P
21018__Orf_virus :-0.0012):0.0044[46]):0.0095[44],
gi_913204007_gb_AKU76621_1__Membrane_protein__Orf_virus :0.0240):0.0115[48],
gi_632123611_gb_AHZ33829_1__membrane_protein__Orf_virus :-0.0008):0.0499[73],((
gi_913204395_gb_AKU77006_1__Membrane_protein__Orf_virus :0.0044,(
gi_41057194_ref_NP_957908_1__ORF131_putative_membrane_protein__Orf_virus :0.0014,(

gi_913204140_gb_AKU76753_1__Membrane_protein__Orf_virus :0.0146,
gi_913204266_gb_AKU76878_1__Membrane_protein__Orf_virus
:0.0178):0.0092[40]):0.0037[24]):0.0115[55],(
gi_289183766_ref_YP_003457307_1__membrane_protein__Pseudocowpox_virus :0.0000,
gi_288804230_gb_ADC53896_1__membrane_protein__Pseudocowpox_virus
:0.0000):0.0369[96]):0.0185[60]):0.0123[30]):0.5720[100]):0.2091[85],(((
gi_582973317_ref_YP_009001691_1__SS_bond_formation_pathway_protein__Anomala_cuprea_e
ntomopoxvirus :0.2638,((
gi_506498247_ref_YP_008004044_1__SS_bond_formation_pathway_protein_substrate__CopF9L
___Adoxophyes_honmai_entomopoxvirus__L :0.0431,((
gi_506498553_ref_YP_008004349_1__SS_bond_formation_pathway_protein_substrate__CopF9L
___Choristoneura_biennis_entomopoxvirus :-0.0022,
gi_506498856_ref_YP_008004650_1__SS_bond_formation_pathway_protein_substrate__CopF9L
___Choristoneura_rosaceana_entomopoxvirus__L :0.0355):0.0415[97],
gi_9964557_ref_NP_065025_1__hypothetical_protein_AMV243__Amsacta_moorei_entomopoxvir
us :0.0149):0.0472[75]):0.0136[52],
gi_506497986_ref_YP_008003785_1__SS_bond_formation_pathway_protein_substrate__CopF9L
___Mythimna_separata_entomopoxvirus__L :0.0519):0.1158[96]):0.0977[83],
gi_9631330_ref_NP_048165_1__ORF_MSV094_putative_membrane_protein__vaccinia_F9L___sim
ilar_to_SW_P24361__Melanoplus_sanguinipes_entomopoxvirus :0.2615):0.1931[91],
gi_51317197_gb_AAT99854_1__putative_membrane_protein__Diachasmimorpha_longicaudata_e
ntomopoxvirus :0.8421):0.0638[46]):0.1112[56],
gi_918014482_ref_YP_009162464_1__myristylated_IMV_envelope_protein_F9L__Salmon_gill_
poxvirus :1.1532):0.1634[76],
gi_918014487_ref_YP_009162469_1__myristylated_IMV_envelope_protein_L1R__Salmon_gill_
poxvirus :0.4699):0.1841[90],
gi_115531746_ref_YP_784272_1__myristylated_IMV_envelope_protein__Nile_crocodilepox_v
irus :0.2622):0.0580[63],(((((
gi_9964531_ref_NP_064999_1__putative_myristylated_membrane_protein__Amsacta_moorei_e
ntomopoxvirus :0.0761,(
gi_506498821_ref_YP_008004615_1__IMV_membrane_protein__CopL1R___Choristoneura_rosace
ana_entomopoxvirus__L :0.0129,
gi_506498520_ref_YP_008004316_1__IMV_membrane_protein__CopL1R___Choristoneura_bienni
s_entomopoxvirus :-0.0028):0.1461[100]):0.0207[16],
gi_506497958_ref_YP_008003757_1__IMV_membrane_protein__CopL1R___Mythimna_separata_en
tomopoxvirus__L :0.1151):0.0199[18],
gi_9631378_ref_NP_048254_1__ORF_MSV183_putative_myristylated_membrane_protein__Mollu
scum_contagiosum_virus_MC069R__vaccinia_L1R__homolog__similar_to_GB_U60315__Melanopl
us_ :0.1314):0.0197[11],
gi_582973196_ref_YP_009001570_1__putative_myristylated_membrane_protein__Anomala_cup
rea_entomopoxvirus :0.0740):0.0132[12],
gi_506498161_ref_YP_008003958_1__IMV_membrane_protein__CopL1R___Adoxophyes_honmai_en
tomopoxvirus__L :0.1061):0.3335[99]):0.1061[87],(
gi_1046611057_ref_YP_009268780_1__imv_membrane_protein__Pteropox_virus :0.1542,(((((
gi_288804151_gb_ADC53817_1__IMV_protein__Pseudocowpox_virus :0.0000,
gi_289183811_ref_YP_003457352_1__IMV_protein__Pseudocowpox_virus
:0.0000):0.0304[96],
gi_738809426_ref_YP_009112786_1__putative_myristylated_IMV_envelope_protein__Parapox
virus_red_deer/HL953 :0.0219):0.0158[58],((((
gi_913203922_gb_AKU76536_1__Myristylated_IMV_envelope_protein__Orf_virus :-0.0021,
gi_325073842_gb_ADY76895_1__PP188__Orf_virus :0.0123):0.0011[46],
gi_41057110_ref_NP_957824_1__ORF047_putative_myristylated_IMV_envelope_protein__Orf_
virus :-0.0011):0.0005[46],
gi_632123526_gb_AHZ33744_1__myristylated_IMV_envelope_protein__Orf_virus :-
0.0005):0.0066[70],(
gi_41018534_gb_AAR98142_1__ORF047_putative_myristylated_IMV_envelope_protein__Orf_vi
rus :0.0056, gi_74230759_gb_ABA00564_1__IMV_protein__Orf_virus
:0.0148):0.0037[25]):0.0023[38]):0.0256[57],((
gi_41057483_ref_NP_957956_1__ORF047_myristylated_IMV_envelope_protein__Bovine_papula
r_stomatitis_virus :0.0000,

gi_806824899_gb_AKC03473_1__myristylated_IMV_envelope_protein__Bovine_papular_stomat
itis_virus :0.0000):0.0000[97],
gi_806824640_gb_AKC03216_1__myristylated_IMV_envelope_protein__Bovine_papular_stomat
itis_virus :0.0000):0.0368[97]):0.0153[51],
gi_1215835349_ref_YP_009389333_1__myristylated_IMV_envelope_proteinlike_protein__Sea
l_parapoxvirus :0.0515):0.1480[99]):0.0399[67]):0.0687[78],((
gi_1158620761_gb_AQY16642_1__MC069__Molluscum_contagiosum_virus_subtype_2 :0.0111,
gi_9629001_ref_NP_044020_1__MC069R__Molluscum_contagiosum_virus_subtype_1
:0.0092):0.0661[98],((((
gi_659488260_ref_YP_009046122_1__myristylated_protein__Penguinpox_virus :-0.0033,(
gi_9634798_ref_NP_039091_1__Myristylated_membrane_protein__Fowlpox_virus :-0.0038,
gi_221404_dbj_BAA00225_1__unnamed_protein_product__Fowlpox_virus
:0.0139):0.0237[87]):0.0100[85],
gi_659488498_ref_YP_009046359_1__myristylated_protein__Pigeonpox_virus
:0.0002):0.0456[96],
gi_946699633_ref_YP_009177112_1__myristylated_membrane_protein__Turkeypox_virus
:0.0693):0.0196[57],((gi_1173596344_gb_ARE67397_1__SWPV2ORF161__Shearwaterpox_virus
:-0.0009,
gi_40556111_ref_NP_955196_1__CNPV173_putative_myristylated_IMV_envelope_protein__Can
arypox_virus :0.0110):0.0244[85],
gi_1174036708_gb_ARF02733_1__SWPV1149__Shearwaterpox_virus
:0.0589):0.0251[65]):0.1153[99]):0.0303[73]):0.0660[71],
gi_571797980_ref_YP_008658479_1__myristylprotein_/_IMV_protein__Squirrelpox_virus
:0.1769):0.0779[93],(((
gi_377829969_ref_YP_005296264_1__unnamed_protein_product__Cotia_virus_SPAn232
:0.0193, gi_1119035641_ref_YP_009329692_1__protein_L1__BeAn_58058_virus
:0.0426):0.0617[96],((gi_539191066_gb_AGU99738_1__m55R__Myxoma_virus :0.0000,
gi_9633691_ref_NP_051769_1__m55R__Myxoma_virus :0.0000):0.0002[83],
gi_9633864_ref_NP_051944_1__gp055R__Rabbit_fibroma_virus
:0.0099):0.1165[100]):0.0116[35],((((
gi_115503135_gb_ABI99053_1__myristylated_IMV_envelope_protein__Deerpox_virus_W117084
:-0.0003,
gi_62637446_ref_YP_227444_1__Myristylated_IMV_envelope_protein__Deerpox_virus_W84883
:0.0106):0.0117[91],(((
gi_148912937_ref_YP_001293251_1__hypothetical_protein_GTPV_gp056__Goatpox_virus_Pell
or :0.0000, gi_154268989_gb_ABS72327_1__L1R__Goatpox_virus :0.0000):0.0105[94],
gi_15150499_ref_NP_150494_1__LSDV060_putative_myristylated_IMV_envelope_protein__Lum
py_skin_disease_virus_NI2490 :-0.0001):0.0091[79],
gi_21492513_ref_NP_659632_1__Myristylated_IMV_envelope_protein__Sheeppox_virus
:0.0117):0.0524[96]):0.0207[58],(
gi_18640143_ref_NP_570217_1__SPV057_putative_myristylated_IMV_envelope_protein__Swin
epox_virus :0.0709,
gi_1237089682_ref_YP_009408015_1__Myristylated_IMV_envelope_protein__Eptesipox_virus
:0.1075):0.0106[21]):0.0081[22],(
gi_38229222_ref_NP_938315_1__60R__Yaba_monkey_tumor_virus :0.0387,
gi_12085043_ref_NP_073445_1__60R_protein__Yabalike_disease_virus
:0.0455):0.0413[82]):0.0421[62]):0.0487[81]):0.0622[92],(
gi_1236513682_ref_YP_009408468_1__IMV_membrane_protein__NY_014_poxvirus :0.0000,
gi_1236513479_ref_YP_009408266_1__IMV_membrane_protein__Murmansk_poxvirus
:0.0000):0.0452[99]):0.0106[50],
gi_345107256_ref_YP_004821421_1__IMV_membrane_protein__Yokapox_virus
:0.0491):0.0549[98],((
gi_1070062787_ref_YP_009282781_1__imv_membrane_protein__Skunkpox_virus :0.0000,
gi_1070099109_ref_YP_009281835_1__imv_membrane_protein__Volepox_virus
:0.0000):0.0205[95],
gi_831934516_ref_YP_009143396_1__IMV_membrane_protein__Raccoonpox_virus
:0.0000):0.0071[63]):0.0043[51],((
gi_90660326_gb_ABD97440_1__IMV_membrane_protein__Cowpox_virus :0.0000,
gi_325558042_gb_ADZ29423_1__IMV_membrane_protein__Cowpox_virus :0.0000):-0.0000[52],
gi_20178464_ref_NP_619885_1__CPXV099_protein__Cowpox_virus

:0.0000):0.0004[50]):0.0049[56],
gi_1210074512_emb_SNB53955_1__CPXV099_protein__Cowpox_virus :0.0054):0.0044[56],(((
gi_29029580_gb_AAN78221_1__M1R__Monkeypox_virus :0.0000,
gi_17974993_ref_NP_536507_1__M1R__Monkeypox_virus_Zaire96I16 :0.0000):0.0000[52],
gi_300872703_gb_ADK39105_1__myristylprotein__Monkeypox_virus :0.0000):-0.0000[47],
gi_661921024_gb_AIE40517_1__myristylprotein__Monkeypox_virus
:0.0000):0.0098[62]):0.0005[29],
gi_562817396_gb_AHB35726_1__IMV_membrane_protein__Vaccinia_virus
:0.0103):0.0001[14], gi_22164677_ref_NP_671590_1__EVM072__Ectromelia_virus
:0.0104):0.0000[8], gi_6969736_gb_AAF33948_1__TL1R__Vaccinia_virus_Tian_Tan
:0.0000):0.0000[7], Query_247142 :0.0000):0.0000[4],
gi_66275885_ref_YP_232970_1__IMV_membrane_protein__Vaccinia_virus
:0.0000):0.0000[3],
gi_113195268_ref_YP_717398_1__IMV_membrane_protein__Taterapox_virus
:0.0000):0.0000[3], gi_1210075651_emb_SNB56540_1__CPXV099_protein__Cowpox_virus
:0.0000):0.0000[3], gi_1210075080_emb_SNB57671_1__CPXV099_protein__Cowpox_virus
:0.0000):0.0000[3], gi_732554838_gb_AIZ72837_1__IMV_membrane_protein__Vaccinia_virus
:0.0000):0.0000[3],
gi_9627594_ref_NP_042117_1__hypothetical_protein_VARVgp073__Variola_virus
:0.0000):0.0000[5],
gi_1143682_emb_CAA53878_1__unnamed_protein_product__Variola_virus :0.0000,
gi_18640320_ref_NP_570476_1__CMLV086__Camelpox_virus :0.0104);

B: F9_L1_gapfree

(((Query_119423 :0.0000,
gi_66275845_ref_YP_232930_1__SS_bond_formation_pathway_protein__Vaccinia_virus
:0.0000):0.0000[42],
gi_325558002_gb_ADZ29383_1__SS_bond_formation_pathway_protein__Cowpox_virus
:0.0000):0.0000[23],
gi_325514063_gb_ADZ24057_1__SS_bond_formation_pathway_protein__Cowpox_virus
:0.0000):0.0000[26], gi_111184232_gb_ABH08152_1__HSPV049__Horsepox_virus
:0.0000):0.0000[21], gi_554571866_gb_AGY97569_1__CPXV056_protein__Cowpox_virus
:0.0000):0.0000[24],
gi_38348914_gb_AAR17890_1__SS_bond_formation_pathway_protein__Vaccinia_virus
:0.0000):0.0000[11], gi_17974954_ref_NP_536468_1__C15L__Monkeypox_virus_Zaire96I16
:0.0000):0.0000[13], gi_44971400_gb_AAS49750_1__RPXV037__Rabbitpox_virus
:0.0000):0.0000[12], gi_20178423_ref_NP_619844_1__CPXV056_protein__Cowpox_virus
:0.0000):0.0000[12], gi_90660286_gb_ABD97400_1__unknown__Cowpox_virus
:0.0000):0.0000[12], gi_18640278_ref_NP_570434_1__CMLV044__Camelpox_virus
:0.0000):0.0000[7],
gi_113195226_ref_YP_717356_1__hypothetical_protein_TATV_DAH68_049__Taterapox_virus
:0.0000):0.0009[9], gi_137849_sp_P24361_1_F9_VACCW_RecName__Full=Protein_F9
:0.0099):0.0007[11],(gi_22164637_ref_NP_671550_1__EVM032__Ectromelia_virus
:0.0083,(((
gi_9627554_ref_NP_042077_1__SS_bond_formation_pathway_protein__Variola_virus :-
0.0052,
gi_544838_gb_AAB29629_1__C13L_product__variola_virus_VAR__India1967__Peptide__212_aa
:0.0162):0.0026[47],
gi_94487114_gb_ABF26214_1__hypothetical_protein_VARV_KUW67_1629_036__Variola_virus
:-0.0026):0.0013[74],
gi_94489329_gb_ABF28418_1__hypothetical_protein_VARV_UNK44_harv_036__Variola_virus
:-0.0013):0.0135[84]):0.0048[42]):0.0054[33],(((
gi_325558218_gb_ADZ29598_1__SS_bond_formation_pathway_protein__Cowpox_virus :-
0.0028,
gi_831934475_ref_YP_009143355_1__SS_bond_formation_pathway_protein__Raccoonpox_virus
:0.0353):0.0014[19], gi_1210076734_emb_SNB49914_1__CPXV056_protein__Cowpox_virus :-
0.0014):0.0037[15],
gi_1169132833_gb_ARB50282_1__SS_bond_formation_pathway_protein__Cowpox_virus
:0.0071):0.0042[17]):0.0208[63],(
gi_1070062745_ref_YP_009282739_1__SS_bond_formation_pathway_protein_substrate__Skunk
pox_virus :0.0223,
gi_1070099067_ref_YP_009281793_1__SS_bond_formation_pathway_protein_substrate__Volep
ox_virus :0.0214):0.0205[75]):0.0289[74],((
gi_1229243048_gb_AST09440_1__SS_bond_formation_pathway_protein_substrate__NY_014_pox
virus :-0.0029,
gi_1229242845_gb_AST09238_1__substrate_for_poxvirus_SS_bond_formation_pathway__Murma
nsk_poxvirus :0.0137):0.0050[66],
gi_345107212_ref_YP_004821377_1__substrate_for_poxvirus_SS_bond_formation_pathway__Y
okapox_virus :0.0852):0.0320[65]):0.0752[81],
gi_1215207784_gb_ASK51226_1__SS_bond_formation_pathway_protein__Eptesipox_virus
:0.3043):0.0599[54],(gi_38229187_ref_NP_938280_1__24L__Yaba_monkey_tumor_virus
:0.0420,(gi_146746361_gb_ABQ43497_1__hypothetical_protein__Tanapox_virus :0.0000,
gi_12085007_ref_NP_073409_1__24L_protein__Yabalike_disease_virus
:0.0000):0.0485[98]):0.1567[99]):0.0359[44],
gi_571797936_ref_YP_008658435_1__envelope_protein_poxlipid_membrane_protein__Squirre
lpox_virus :0.3216):0.0756[31],(((
gi_62637409_ref_YP_227407_1__hypothetical_protein_DpV83gp031__Deerpox_virus_W84883
:0.0141,
gi_115503098_gb_ABI99016_1__hypothetical_protein_DpV84gp031__Deerpox_virus_W117084
:0.0523):0.1875[99],((((
gi_15150463_ref_NP_150458_1__LSDV024_hypothetical_protein__Lumpy_skin_disease_virus_
NI2490 :-0.0042,((

gi_557370484_gb_AGZ95339_1__hypothetical_protein__Goatpox_virus_FZ :-0.0004,
gi_13876676_gb_AAK43564_1__unknown__Lumpy_skin_disease_virus :0.0221):0.0020[29],
gi_148912901_ref_YP_001293215_1__hypothetical_protein_GTPV_gp021__Goatpox_virus_Pell
or :0.0089):0.0095[43]):0.0021[43],
gi_22595717_gb_AAN02749_1__hypothetical_protein__Lumpy_skin_disease_virus :-
0.0021):0.0011[74],
gi_21492478_ref_NP_659597_1__hypothetical_protein_SPPV_21__Sheeppox_virus :-
0.0011):0.1587[100],
gi_18640107_ref_NP_570181_1__SPV021_hypothetical_protein__Swinepox_virus
:0.1251):0.0571[69]):0.0177[16],((
gi_377830027_ref_YP_005296228_1__unnamed_protein_product__Cotia_virus_SPAn232
:0.0680, gi_1119035593_ref_YP_009329644_1__protein_F9__BeAn_58058_virus
:0.0655):0.1845[99],(gi_9633655_ref_NP_051733_1__M019L__Myxoma_virus :-0.0001,((
gi_539191030_gb_AGU99702_1__M019L__Myxoma_virus :0.0000,
gi_982818174_gb_AMB18352_1__M019L__Myxoma_virus :0.0000):0.0234[91],
gi_9633830_ref_NP_051908_1__gp019L__Rabbit_fibroma_virus
:0.0314):0.0163[69]):0.1994[99]):0.0300[28]):0.0360[38]):0.0736[41],((
gi_1158620706_gb_AQY16587_1__MC016__Molluscum_contagiosum_virus_subtype_2 :0.0266,
gi_9628948_ref_NP_043967_1__MC016L__Molluscum_contagiosum_virus_subtype_1
:0.0288):0.2402[100],
gi_115531700_ref_YP_784226_1__hypothetical_protein_CRV036__Nile_crocodilepox_virus
:0.3511):0.1166[51]):0.0531[16],
gi_1046611009_ref_YP_009268732_1__ss_bond_formation_pathway_protein__Pteropox_virus
:0.3249):0.0540[25],((((
gi_659488485_ref_YP_009046346_1__hypothetical_protein_fep_114__Pigeonpox_virus :-
0.0020,
gi_659488247_ref_YP_009046109_1__hypothetical_protein_pepv_116__Penguinpox_virus
:0.0347):0.0075[60],
gi_9634782_ref_NP_039075_1__hypothetical_protein_FPV112__Fowlpox_virus
:0.0033):0.0939[96],(gi_1173596604_gb_ARE67656_1__SWPV1128__Shearwaterpox_virus
:0.0653,
gi_40556077_ref_NP_955162_1__CNPV139_conserved_hypothetical_protein__Canarypox_virus
:0.0518):0.0638[74]):0.0152[47],
gi_946699624_ref_YP_009177103_1__hypothetical_protein__Turkeypox_virus
:0.1378):0.3210[100]):0.0929[35],((
gi_1215835429_ref_YP_009389413_1__vaccinia_virus_F9Llike_protein__Seal_parapoxvirus
:0.0629,((((
gi_28261211_gb_AAO31709_1__vaccinia_virus_F9Llike_protein__Bovine_papular_stomatitis
_virus :0.0031,
gi_806824981_gb_AKC03555_1__putative_membrane_protein__Bovine_papular_stomatitis_vir
us :0.0184):0.0045[84],
gi_41057566_ref_NP_958039_1__ORF131_putative_membrane_protein__Bovine_papular_stomat
itis_virus :-0.0045):0.0023[83],
gi_806824721_gb_AKC03297_1__putative_membrane_protein__Bovine_papular_stomatitis_vir
us :-0.0023):0.1008[100],
gi_738809510_ref_YP_009112870_1__putative_membrane_protein__Parapoxvirus_red_deer/HL
953 :0.1170):0.0532[66]):0.0298[41],((((
gi_28261202_gb_AAO31701_1__vaccinia_virus_F9Llike_protein__Orf_virus_strain_D1701
:0.0172,((gi_576864655_gb_AHH34314_1__putative_membrane_protein__Orf_virus :-
0.0024, gi_41018618_gb_AAR98226_1__ORF131_putative_membrane_protein__Orf_virus
:0.0131):0.0012[42],
gi_915529_gb_AAA86391_1__similar_to_vaccinia_virus_F9L__SwissProt_Accession_Number_P
21018__Orf_virus :-0.0012):0.0044[46]):0.0095[44],
gi_913204007_gb_AKU76621_1__Membrane_protein__Orf_virus :0.0240):0.0115[48],
gi_632123611_gb_AHZ33829_1__membrane_protein__Orf_virus :-0.0008):0.0499[73],((
gi_913204395_gb_AKU77006_1__Membrane_protein__Orf_virus :0.0044,(
gi_41057194_ref_NP_957908_1__ORF131_putative_membrane_protein__Orf_virus :0.0014,(
gi_913204140_gb_AKU76753_1__Membrane_protein__Orf_virus :0.0146,
gi_913204266_gb_AKU76878_1__Membrane_protein__Orf_virus
:0.0178):0.0092[40]):0.0037[24]):0.0115[55],(

gi_289183766_ref_YP_003457307_1__membrane_protein__Pseudocowpox_virus :0.0000,
gi_288804230_gb_ADC53896_1__membrane_protein__Pseudocowpox_virus
:0.0000):0.0369[96]):0.0185[60]):0.0123[30]):0.5720[100]):0.2091[85],(((
gi_582973317_ref_YP_009001691_1__SS_bond_formation_pathway_protein__Anomala_cuprea_e
ntomopoxvirus :0.2638,((
gi_506498247_ref_YP_008004044_1__SS_bond_formation_pathway_protein_substrate__CopF9L
___Adoxophyes_honmai_entomopoxvirus__L :0.0431,((
gi_506498553_ref_YP_008004349_1__SS_bond_formation_pathway_protein_substrate__CopF9L
___Choristoneura_biennis_entomopoxvirus :-0.0022,
gi_506498856_ref_YP_008004650_1__SS_bond_formation_pathway_protein_substrate__CopF9L
___Choristoneura_rosaceana_entomopoxvirus__L :0.0355):0.0415[97],
gi_9964557_ref_NP_065025_1__hypothetical_protein_AMV243__Amsacta_moorei_entomopoxvir
us :0.0149):0.0472[75]):0.0136[52],
gi_506497986_ref_YP_008003785_1__SS_bond_formation_pathway_protein_substrate__CopF9L
___Mythimna_separata_entomopoxvirus__L :0.0519):0.1158[96]):0.0977[83],
gi_9631330_ref_NP_048165_1__ORF_MSV094_putative_membrane_protein__vaccinia_F9L___sim
ilar_to_SW_P24361__Melanoplus_sanguinipes_entomopoxvirus :0.2615):0.1931[91],
gi_51317197_gb_AAT99854_1__putative_membrane_protein__Diachasmimorpha_longicaudata_e
ntomopoxvirus :0.8421):0.0638[46]):0.1112[56],
gi_918014482_ref_YP_009162464_1__myristylated_IMV_envelope_protein_F9L__Salmon_gill_
poxvirus :1.1532):0.1634[76],
gi_918014487_ref_YP_009162469_1__myristylated_IMV_envelope_protein_L1R__Salmon_gill_
poxvirus :0.4699):0.1841[90],
gi_115531746_ref_YP_784272_1__myristylated_IMV_envelope_protein__Nile_crocodilepox_v
irus :0.2622):0.0580[63],(((((
gi_9964531_ref_NP_064999_1__putative_myristylated_membrane_protein__Amsacta_moorei_e
ntomopoxvirus :0.0761,(
gi_506498821_ref_YP_008004615_1__IMV_membrane_protein__CopL1R___Choristoneura_rosace
ana_entomopoxvirus__L :0.0129,
gi_506498520_ref_YP_008004316_1__IMV_membrane_protein__CopL1R___Choristoneura_bienni
s_entomopoxvirus :-0.0028):0.1461[100]):0.0207[16],
gi_506497958_ref_YP_008003757_1__IMV_membrane_protein__CopL1R___Mythimna_separata_en
tomopoxvirus__L :0.1151):0.0199[18],
gi_9631378_ref_NP_048254_1__ORF_MSV183_putative_myristylated_membrane_protein__Mollu
scum_contagiosum_virus_MC069R__vaccinia_L1R__homolog__similar_to_GB_U60315__Melanopl
us_ :0.1314):0.0197[11],
gi_582973196_ref_YP_009001570_1__putative_myristylated_membrane_protein__Anomala_cup
rea_entomopoxvirus :0.0740):0.0132[12],
gi_506498161_ref_YP_008003958_1__IMV_membrane_protein__CopL1R___Adoxophyes_honmai_en
tomopoxvirus__L :0.1061):0.3335[99]):0.1061[87],(
gi_1046611057_ref_YP_009268780_1__imv_membrane_protein__Pteropox_virus :0.1542,(((((
gi_288804151_gb_ADC53817_1__IMV_protein__Pseudocowpox_virus :0.0000,
gi_289183811_ref_YP_003457352_1__IMV_protein__Pseudocowpox_virus
:0.0000):0.0304[96],
gi_738809426_ref_YP_009112786_1__putative_myristylated_IMV_envelope_protein__Parapox
virus_red_deer/HL953 :0.0219):0.0158[58],((((
gi_913203922_gb_AKU76536_1__Myristylated_IMV_envelope_protein__Orf_virus :-0.0021,
gi_325073842_gb_ADY76895_1__PP188__Orf_virus :0.0123):0.0011[46],
gi_41057110_ref_NP_957824_1__ORF047_putative_myristylated_IMV_envelope_protein__Orf_
virus :-0.0011):0.0005[46],
gi_632123526_gb_AHZ33744_1__myristylated_IMV_envelope_protein__Orf_virus :-
0.0005):0.0066[70],(
gi_41018534_gb_AAR98142_1__ORF047_putative_myristylated_IMV_envelope_protein__Orf_vi
rus :0.0056, gi_74230759_gb_ABA00564_1__IMV_protein__Orf_virus
:0.0148):0.0037[25]):0.0023[38]):0.0256[57],((
gi_41057483_ref_NP_957956_1__ORF047_myristylated_IMV_envelope_protein__Bovine_papula
r_stomatitis_virus :0.0000,
gi_806824899_gb_AKC03473_1__myristylated_IMV_envelope_protein__Bovine_papular_stomat
itis_virus :0.0000):0.0000[97],
gi_806824640_gb_AKC03216_1__myristylated_IMV_envelope_protein__Bovine_papular_stomat

itis_virus :0.0000):0.0368[97]):0.0153[51],
gi_1215835349_ref_YP_009389333_1__myristylated_IMV_envelope_proteinlike_protein__Sea
l_parapoxvirus :0.0515):0.1480[99]):0.0399[67]):0.0687[78],((
gi_1158620761_gb_AQY16642_1__MC069__Molluscum_contagiosum_virus_subtype_2 :0.0111,
gi_9629001_ref_NP_044020_1__MC069R__Molluscum_contagiosum_virus_subtype_1
:0.0092):0.0661[98],((((
gi_659488260_ref_YP_009046122_1__myristylated_protein__Penguinpox_virus :-0.0033,(
gi_9634798_ref_NP_039091_1__Myristylated_membrane_protein__Fowlpox_virus :-0.0038,
gi_221404_dbj_BAA00225_1__unnamed_protein_product__Fowlpox_virus
:0.0139):0.0237[87]):0.0100[85],
gi_659488498_ref_YP_009046359_1__myristylated_protein__Pigeonpox_virus
:0.0002):0.0456[96],
gi_946699633_ref_YP_009177112_1__myristylated_membrane_protein__Turkeypox_virus
:0.0693):0.0196[57],((gi_1173596344_gb_ARE67397_1__SWPV2ORF161__Shearwaterpox_virus
:-0.0009,
gi_40556111_ref_NP_955196_1__CNPV173_putative_myristylated_IMV_envelope_protein__Can
arypox_virus :0.0110):0.0244[85],
gi_1174036708_gb_ARF02733_1__SWPV1149__Shearwaterpox_virus
:0.0589):0.0251[65]):0.1153[99]):0.0303[73]):0.0660[71],
gi_571797980_ref_YP_008658479_1__myristylprotein_/_IMV_protein__Squirrelpox_virus
:0.1769):0.0779[93],(((
gi_377829969_ref_YP_005296264_1__unnamed_protein_product__Cotia_virus_SPAn232
:0.0193, gi_1119035641_ref_YP_009329692_1__protein_L1__BeAn_58058_virus
:0.0426):0.0617[96],((gi_539191066_gb_AGU99738_1__m55R__Myxoma_virus :0.0000,
gi_9633691_ref_NP_051769_1__m55R__Myxoma_virus :0.0000):0.0002[83],
gi_9633864_ref_NP_051944_1__gp055R__Rabbit_fibroma_virus
:0.0099):0.1165[100]):0.0116[35],((((
gi_115503135_gb_ABI99053_1__myristylated_IMV_envelope_protein__Deerpox_virus_W117084
:-0.0003,
gi_62637446_ref_YP_227444_1__Myristylated_IMV_envelope_protein__Deerpox_virus_W84883
:0.0106):0.0117[91],(((
gi_148912937_ref_YP_001293251_1__hypothetical_protein_GTPV_gp056__Goatpox_virus_Pell
or :0.0000, gi_154268989_gb_ABS72327_1__L1R__Goatpox_virus :0.0000):0.0105[94],
gi_15150499_ref_NP_150494_1__LSDV060_putative_myristylated_IMV_envelope_protein__Lum
py_skin_disease_virus_NI2490 :-0.0001):0.0091[79],
gi_21492513_ref_NP_659632_1__Myristylated_IMV_envelope_protein__Sheeppox_virus
:0.0117):0.0524[96]):0.0207[58],(
gi_18640143_ref_NP_570217_1__SPV057_putative_myristylated_IMV_envelope_protein__Swin
epox_virus :0.0709,
gi_1237089682_ref_YP_009408015_1__Myristylated_IMV_envelope_protein__Eptesipox_virus
:0.1075):0.0106[21]):0.0081[22],(
gi_38229222_ref_NP_938315_1__60R__Yaba_monkey_tumor_virus :0.0387,
gi_12085043_ref_NP_073445_1__60R_protein__Yabalike_disease_virus
:0.0455):0.0413[82]):0.0421[62]):0.0487[81]):0.0622[92],(
gi_1236513682_ref_YP_009408468_1__IMV_membrane_protein__NY_014_poxvirus :0.0000,
gi_1236513479_ref_YP_009408266_1__IMV_membrane_protein__Murmansk_poxvirus
:0.0000):0.0452[99]):0.0106[50],
gi_345107256_ref_YP_004821421_1__IMV_membrane_protein__Yokapox_virus
:0.0491):0.0549[98],((
gi_1070062787_ref_YP_009282781_1__imv_membrane_protein__Skunkpox_virus :0.0000,
gi_1070099109_ref_YP_009281835_1__imv_membrane_protein__Volepox_virus
:0.0000):0.0205[95],
gi_831934516_ref_YP_009143396_1__IMV_membrane_protein__Raccoonpox_virus
:0.0000):0.0071[63]):0.0043[51],((
gi_90660326_gb_ABD97440_1__IMV_membrane_protein__Cowpox_virus :0.0000,
gi_325558042_gb_ADZ29423_1__IMV_membrane_protein__Cowpox_virus :0.0000):-0.0000[52],
gi_20178464_ref_NP_619885_1__CPXV099_protein__Cowpox_virus
:0.0000):0.0004[50]):0.0049[56],
gi_1210074512_emb_SNB53955_1__CPXV099_protein__Cowpox_virus :0.0054):0.0044[56],(((
gi_29029580_gb_AAN78221_1__M1R__Monkeypox_virus :0.0000,

gi_17974993_ref_NP_536507_1__M1R__Monkeypox_virus_Zaire96I16 :0.0000):0.0000[52],
gi_300872703_gb_ADK39105_1__myristylprotein__Monkeypox_virus :0.0000):-0.0000[47],
gi_661921024_gb_AIE40517_1__myristylprotein__Monkeypox_virus
:0.0000):0.0098[62]):0.0005[29],
gi_562817396_gb_AHB35726_1__IMV_membrane_protein__Vaccinia_virus
:0.0103):0.0001[14], gi_22164677_ref_NP_671590_1__EVM072__Ectromelia_virus
:0.0104):0.0000[8], gi_6969736_gb_AAF33948_1__TL1R__Vaccinia_virus_Tian_Tan
:0.0000):0.0000[7], Query_247142 :0.0000):0.0000[4],
gi_66275885_ref_YP_232970_1__IMV_membrane_protein__Vaccinia_virus
:0.0000):0.0000[3],
gi_113195268_ref_YP_717398_1__IMV_membrane_protein__Taterapox_virus
:0.0000):0.0000[3], gi_1210075651_emb_SNB56540_1__CPXV099_protein__Cowpox_virus
:0.0000):0.0000[3], gi_1210075080_emb_SNB57671_1__CPXV099_protein__Cowpox_virus
:0.0000):0.0000[3], gi_732554838_gb_AIZ72837_1__IMV_membrane_protein__Vaccinia_virus
:0.0000):0.0000[3],
gi_9627594_ref_NP_042117_1__hypothetical_protein_VARVgp073__Variola_virus
:0.0000):0.0000[5],
gi_1143682_emb_CAA53878_1__unnamed_protein_product__Variola_virus :0.0000,
gi_18640320_ref_NP_570476_1__CMLV086__Camelpox_virus :0.0104);

Supplemental figure 1

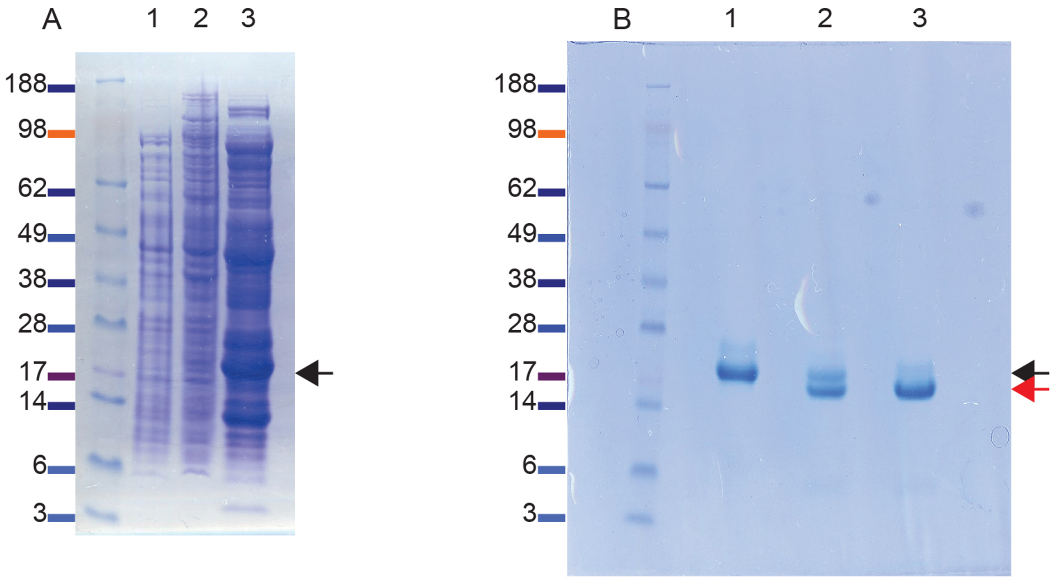

Supplemental figure 2

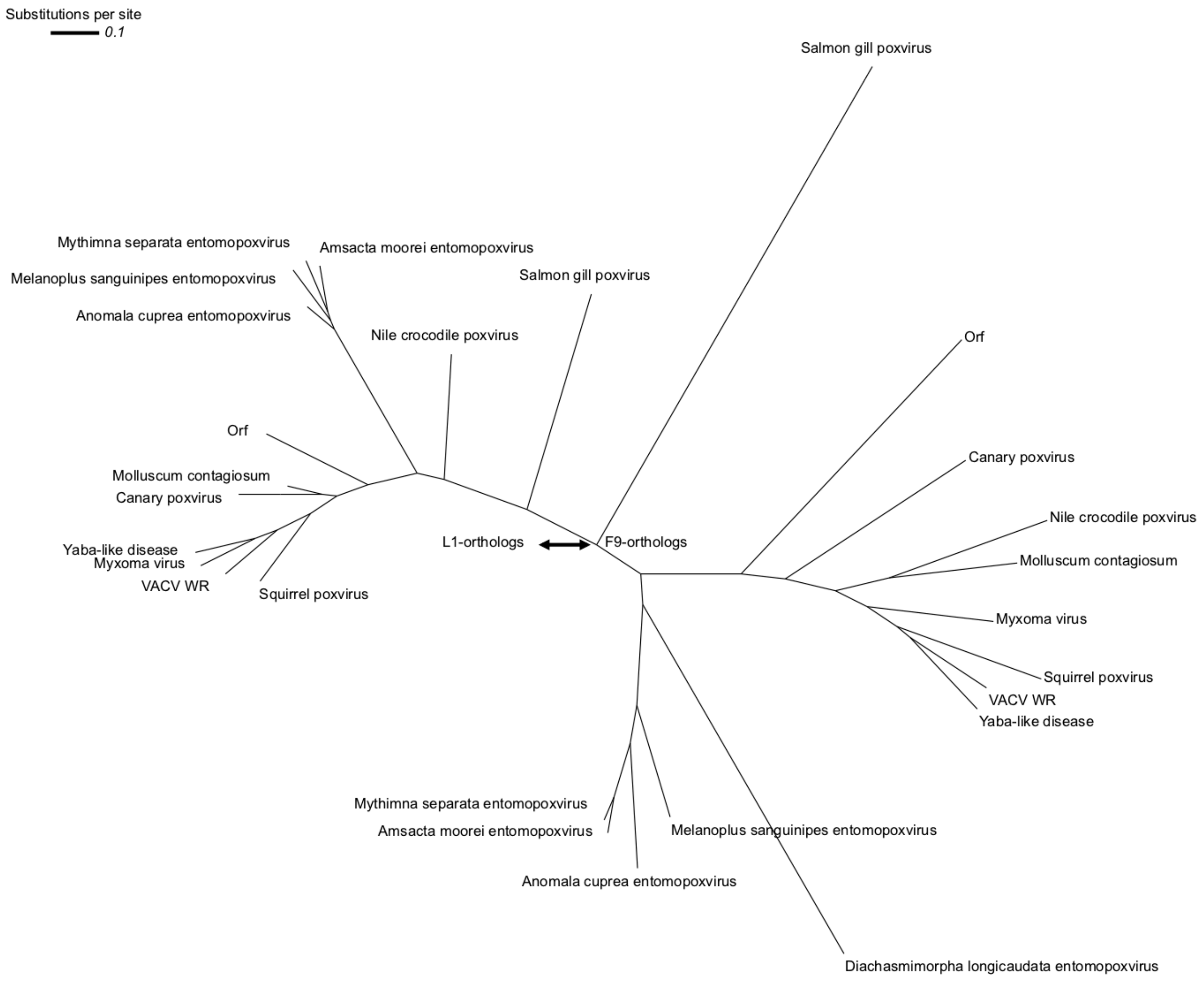

Supplement: Supplementary file 1 — Supplemental Material [file 41598_2018_34244_MOESM1_ESM.pdf]
