# Supplementary material for: The 2.1 Å structure of protein F9 and its comparison to L1, two components of the conserved poxvirus entry-fusion complex
Source: Sci Rep. 2018 Nov 14;8:16807. doi: 10.1038/s41598-018-34244-7 (PMC6235832; doi:10.1038/s41598-018-34244-7)
Supplement: Supplementary file 3 — Quality report [file 41598_2018_34244_MOESM3_ESM.pdf]

Preliminary Full wwPDB X-ray Structure Validation Report (i)

Feb 23, 2018 – 02:47 PM EST

Deposition ID	:	D_1000232800
PDB ID	:	*(not yet assigned)*

This is a Preliminary Full wwPDB X-ray Structure Validation Report.

This report is produced by the wwPDB Deposition System during initial deposition but before annotation of the structure.

We welcome your comments at *validation@mail.wwpdb.org*
A user guide is available at
http://wwpdb.org/validation/2016/XrayValidationReportHelp
with specific help available everywhere you see the (i) symbol.

PRELIMINARY VALIDATION REPORT

The following versions of software and data (see references (i)) were used in the production of this report:

MolProbity	:	4.02b-467
Mogul	:	1.7.2 (RC1), CSD as538be (2017)
Xtriage (Phenix)	:	1.9-1692
EDS	:	rb-20030736
Percentile statistics	:	20161228.v01 (using entries in the PDB archive December 28th 2016)
Refmac	:	5.8.0135
CCP4	:	6.5.0
Ideal geometry (proteins)	:	Engh & Huber (2001)
Ideal geometry (DNA, RNA)	:	Parkinson et al. (1996)
Validation Pipeline (wwPDB-VP)	:	rb-20030736

1 Overall quality at a glance (i)

The following experimental techniques were used to determine the structure:
X-RAY DIFFRACTION

The reported resolution of this entry is 2.10 Å.

Percentile scores (ranging between 0-100) for global validation metrics of the entry are shown in the following graphic. The table shows the number of entries on which the scores are based.

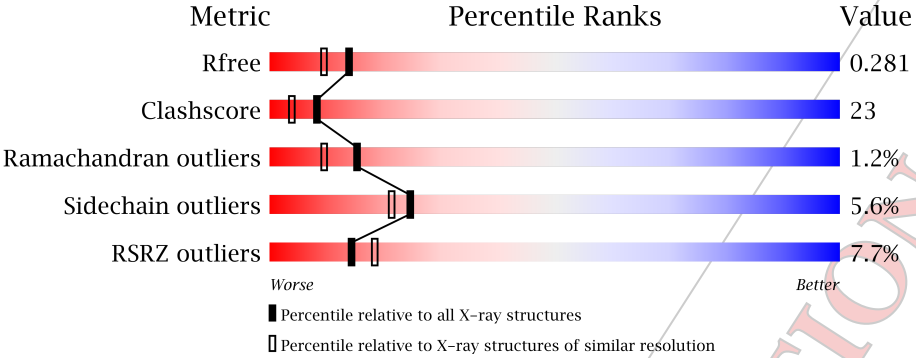

Metric	Whole archive (#Entries)	Similar resolution (#Entries, resolution range(Å))
R_{free}	100719	4243 (2.10-2.10)
Clashscore	112137	4788 (2.10-2.10)
Ramachandran outliers	110173	4740 (2.10-2.10)
Sidechain outliers	110143	4741 (2.10-2.10)
RSRZ outliers	101464	4275 (2.10-2.10)

The table below summarises the geometric issues observed across the polymeric chains and their fit to the electron density. The red, orange, yellow and green segments on the lower bar indicate the fraction of residues that contain outliers for >=3, 2, 1 and 0 types of geometric quality criteria. A grey segment represents the fraction of residues that are not modelled. The numeric value for each fraction is indicated below the corresponding segment, with a dot representing fractions <=5% The upper red bar (where present) indicates the fraction of residues that have poor fit to the electron density. The numeric value is given above the bar.

Mol	Chain	Length	Quality of chain
1	A	176	10% / 64% 28% • 6%
1	B	176	6% / 65% 22% 5% 9%
1	C	176	5% / 68% 22% • 9%
2	D	166	8% / 70% 28% •

The following table lists non-polymeric compounds, carbohydrate monomers and non-standard residues in protein, DNA, RNA chains that are outliers for geometric or electron-density-fit crite-

PRELIMINARY VALIDATION REPORT

ria:

Mol	Type	Chain	Res	Chirality	Geometry	Clashes	Electron density
11	PDO	H	10	-	-	X	X
11	PDO	H	12	-	-	-	X
11	PDO	H	3	-	-	-	X
11	PDO	H	6	-	-	X	X
11	PDO	H	7	-	-	-	X
12	EOH	I	11	-	-	-	X
12	EOH	I	13	-	-	X	-
12	EOH	I	15	-	-	X	X
12	EOH	I	16	-	-	X	-
12	EOH	I	19	-	-	X	X
12	EOH	I	28	-	-	X	-
12	EOH	I	31	-	-	-	X
12	EOH	I	33	-	-	X	-
12	EOH	I	43	-	-	X	X
12	EOH	I	45	-	-	-	X
12	EOH	I	5	-	-	X	-
13	MOH	J	2	-	-	-	X
13	MOH	J	21	-	-	X	-
13	MOH	J	6	-	-	-	X
13	MOH	J	7	-	-	-	X
13	MOH	J	9	-	-	-	X
14	PGO	K	1	X	-	-	-
14	PGO	K	4	-	-	X	-
14	PGO	K	5	-	-	X	-
14	PGO	K	8	-	-	-	X
14	PGO	K	9	-	-	X	-
15	PGE	L	4	-	-	-	X
16	ETX	N	1	-	-	X	-
16	ETX	N	2	-	-	X	-
16	ETX	N	4	-	-	X	-
3	PG4	E	4	-	-	X	X
4	PG0	P	1	-	-	-	X
4	PG0	P	4	-	-	-	X
4	PG0	P	6	-	-	X	X
4	PG0	P	7	-	-	-	X
5	PEG	M	1	-	-	X	X
5	PEG	M	2	-	-	X	X
5	PEG	M	4	-	-	X	X
5	PEG	M	5	-	-	-	X
5	PEG	M	6	-	-	X	X
5	PEG	M	7	-	-	X	-
5	PEG	M	8	-	-	-	X

Continued on next page...

PRELIMINARY VALIDATION REPORT

Continued from previous page...

Mol	Type	Chain	Res	Chirality	Geometry	Clashes	Electron density
6	P6G	Q	2	-	-	X	-
7	P33	R	1	-	-	X	X
9	EDO	F	10	-	-	-	X
9	EDO	F	15	-	-	-	X
9	EDO	F	16	-	-	-	X
9	EDO	F	19	-	-	-	X
9	EDO	F	2	-	-	-	X
9	EDO	F	20	-	-	-	X
9	EDO	F	25	-	-	-	X

PRELIMINARY VALIDATION REPORT

2 Entry composition (i)

There are 17 unique types of molecules in this entry. The entry contains 6062 atoms, of which 0 are hydrogens and 0 are deuteriums.

In the tables below, the ZeroOcc column contains the number of atoms modelled with zero occupancy, the AltConf column contains the number of residues with at least one atom in alternate conformation and the Trace column contains the number of residues modelled with at most 2 atoms.

- Molecule 1 is a protein called Poxvirus entry fusion complex associated protein F9.

Mol	Chain	Residues	Atoms					ZeroOcc	AltConf	Trace
1	A	165	Total 1239	C 781	N 206	O 243	S 9	0	0	0
1	B	161	Total 1211	C 759	N 202	O 241	S 9	0	0	0
1	C	161	Total 1226	C 767	N 204	O 246	S 9	0	1	0

- Molecule 2 is a protein.

Mol	Chain	Residues	Atoms					ZeroOcc	AltConf	Trace
2	D	166	Total 1271	C 800	N 214	O 247	S 10	0	1	0

- Molecule 3 is TETRAETHYLENE GLYCOL (three-letter code: PG4) (formula: $C_8H_{18}O_5$).

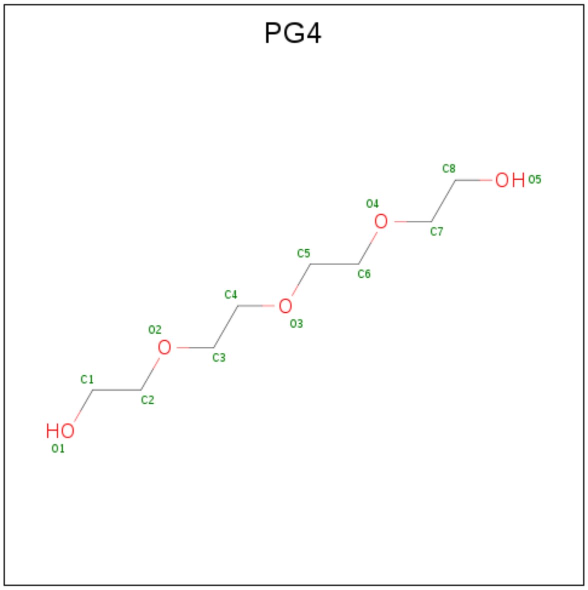

PRELIMINARY VALIDATION REPORT

Mol	Chain	Residues	Atoms	ZeroOcc	AltConf
3	E	1	Total C O<br>13 8 5	0	0
3	E	1	Total C O<br>13 8 5	0	0
3	E	1	Total C O<br>13 8 5	0	0
3	E	1	Total C O<br>13 8 5	0	0
3	E	1	Total C O<br>13 8 5	0	0

- Molecule 4 is 2-(2-METHOXYETHOXY)ETHANOL (three-letter code: PG0) (formula: $C_5H_{12}O_3$).

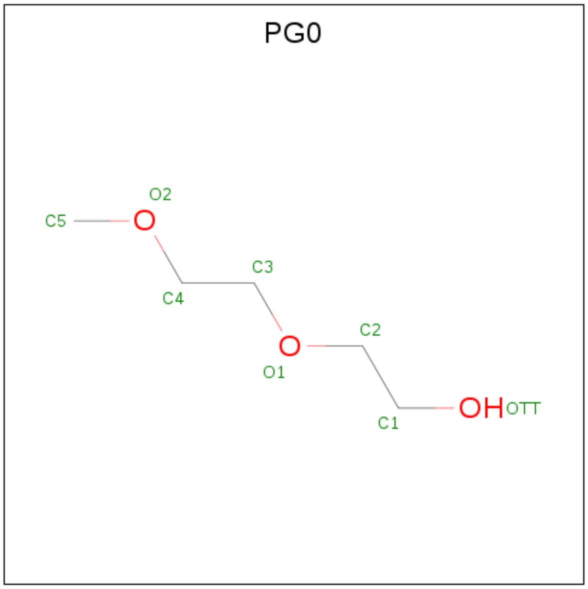

Mol	Chain	Residues	Atoms	ZeroOcc	AltConf
4	P	1	Total C O<br>8 5 3	0	0
4	P	1	Total C O<br>8 5 3	0	0
4	P	1	Total C O<br>8 5 3	0	0
4	P	1	Total C O<br>8 5 3	0	0
4	P	1	Total C O<br>8 5 3	0	0
4	P	1	Total C O<br>8 5 3	0	0

Continued on next page...

PRELIMINARY VALIDATION REPORT

Continued from previous page...

Mol	Chain	Residues	Atoms	ZeroOcc	AltConf
4	P	1	Total C O<br>8 5 3	0	0
4	P	1	Total C O<br>8 5 3	0	0
4	P	1	Total C O<br>8 5 3	0	0

- Molecule 5 is DI(HYDROXYETHYL)ETHER (three-letter code: PEG) (formula: $C_4H_{10}O_3$).

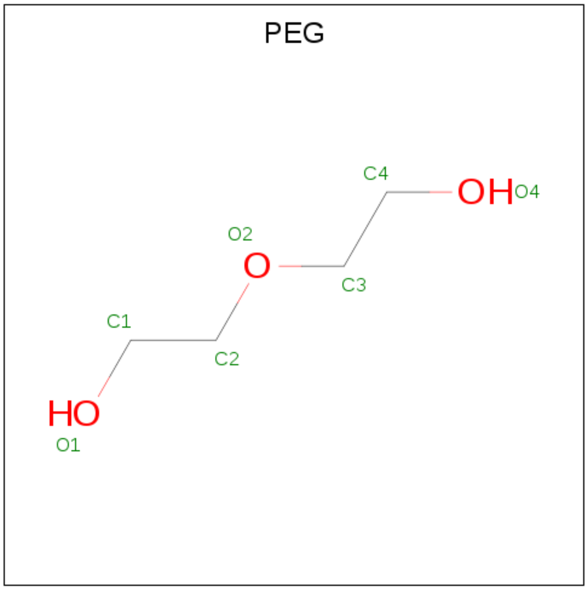

Mol	Chain	Residues	Atoms	ZeroOcc	AltConf
5	M	1	Total C O<br>7 4 3	0	0
5	M	1	Total C O<br>7 4 3	0	0
5	M	1	Total C O<br>7 4 3	0	0
5	M	1	Total C O<br>7 4 3	0	0
5	M	1	Total C O<br>7 4 3	0	0
5	M	1	Total C O<br>7 4 3	0	0
5	M	1	Total C O<br>7 4 3	0	0
5	M	1	Total C O<br>7 4 3	0	0

Continued on next page...

PRELIMINARY VALIDATION REPORT

Continued from previous page...

Mol	Chain	Residues	Atoms	ZeroOcc	AltConf
5	M	1	Total C O<br>7 4 3	0	0
5	M	1	Total C O<br>7 4 3	0	0
5	M	1	Total C O<br>7 4 3	0	0

- Molecule 6 is HEXAETHYLENE GLYCOL (three-letter code: P6G) (formula: $C_{12}H_{26}O_7$).

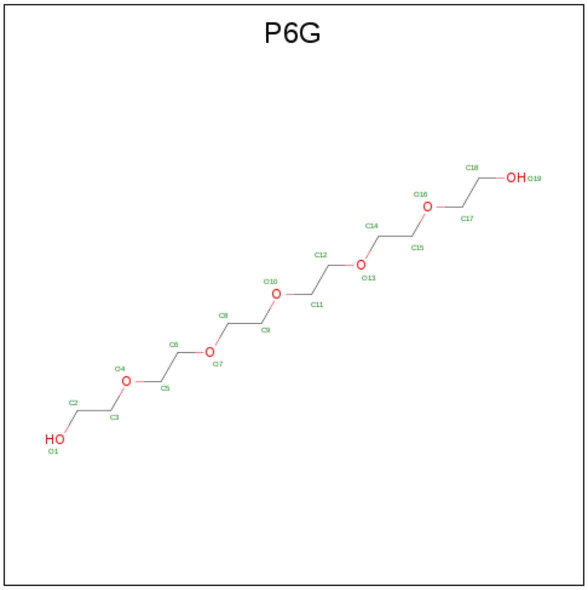

Mol	Chain	Residues	Atoms	ZeroOcc	AltConf
6	Q	1	Total C O<br>19 12 7	0	0
6	Q	1	Total C O<br>19 12 7	0	0
6	Q	1	Total C O<br>19 12 7	0	0

- Molecule 7 is 3,6,9,12,15,18-HEXAOXAICOSANE-1,20-DIOL (three-letter code: P33) (formula: $C_{14}H_{30}O_8$).

PRELIMINARY VALIDATION REPORT

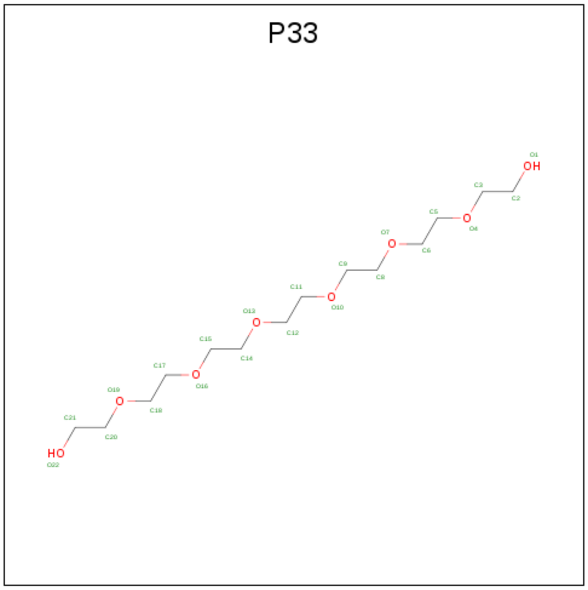

Mol	Chain	Residues	Atoms	ZeroOcc	AltConf
7	R	1	Total C O<br>22 14 8	0	0

- Molecule 8 is 2-{2-[2-2-(METHOXY-ETHOXY)-ETHOXY]-ETHOXY}-ETHANOL (three-letter code: ETE) (formula: $C_9H_{20}O_5$).

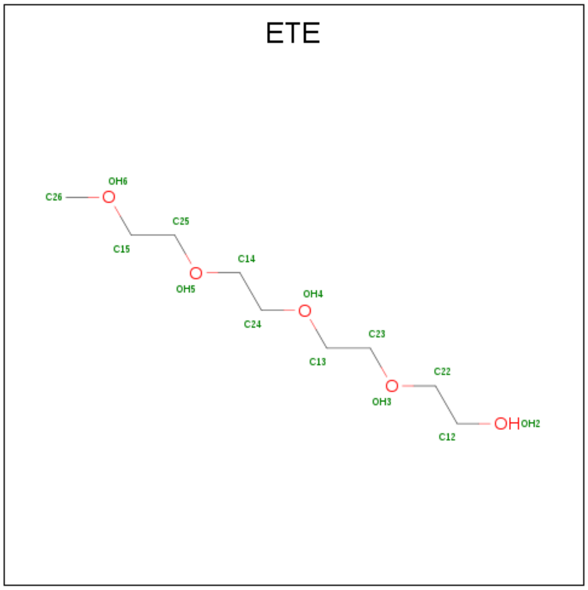

Mol	Chain	Residues	Atoms	ZeroOcc	AltConf
8	S	1	Total C O<br>14 9 5	0	0
8	S	1	Total C O<br>14 9 5	0	0

PRELIMINARY VALIDATION REPORT

- Molecule 9 is 1,2-ETHANEDIOL (three-letter code: EDO) (formula: $C_2H_6O_2$).

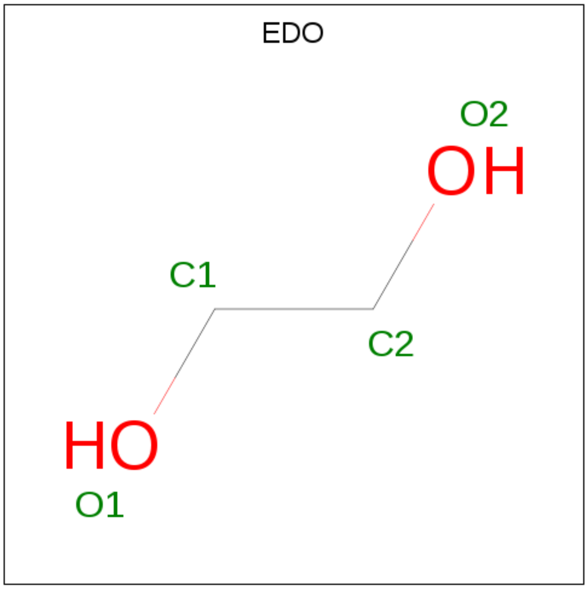

Mol	Chain	Residues	Atoms	ZeroOcc	AltConf
9	F	1	Total C O<br>4 2 2	0	0
9	F	1	Total C O<br>4 2 2	0	0
9	F	1	Total C O<br>4 2 2	0	0
9	F	1	Total C O<br>4 2 2	0	0
9	F	1	Total C O<br>4 2 2	0	0
9	F	1	Total C O<br>4 2 2	0	0
9	F	1	Total C O<br>4 2 2	0	0
9	F	1	Total C O<br>4 2 2	0	0
9	F	1	Total C O<br>4 2 2	0	0
9	F	1	Total C O<br>4 2 2	0	0
9	F	1	Total C O<br>4 2 2	0	0
9	F	1	Total C O<br>4 2 2	0	0
9	F	1	Total C O<br>4 2 2	0	0

Continued on next page...

PRELIMINARY VALIDATION REPORT

Continued from previous page...

Mol	Chain	Residues	Atoms	ZeroOcc	AltConf
9	F	1	Total C O<br>4 2 2	0	0
9	F	1	Total C O<br>4 2 2	0	0
9	F	1	Total C O<br>4 2 2	0	0
9	F	1	Total C O<br>4 2 2	0	0
9	F	1	Total C O<br>4 2 2	0	0
9	F	1	Total C O<br>4 2 2	0	0
9	F	1	Total C O<br>4 2 2	0	0
9	F	1	Total C O<br>4 2 2	0	0
9	F	1	Total C O<br>4 2 2	0	0
9	F	1	Total C O<br>4 2 2	0	0
9	F	1	Total C O<br>4 2 2	0	0
9	F	1	Total C O<br>4 2 2	0	0
9	F	1	Total C O<br>4 2 2	0	0
9	F	1	Total C O<br>4 2 2	0	0
9	F	1	Total C O<br>4 2 2	0	0
9	F	1	Total C O<br>4 2 2	0	0
9	F	1	Total C O<br>4 2 2	0	0
9	F	1	Total C O<br>4 2 2	0	0
9	F	1	Total C O<br>4 2 2	0	0
9	F	1	Total C O<br>4 2 2	0	0

- Molecule 10 is GLYCEROL (three-letter code: GOL) (formula: $C_3H_8O_3$).

PRELIMINARY VALIDATION REPORT

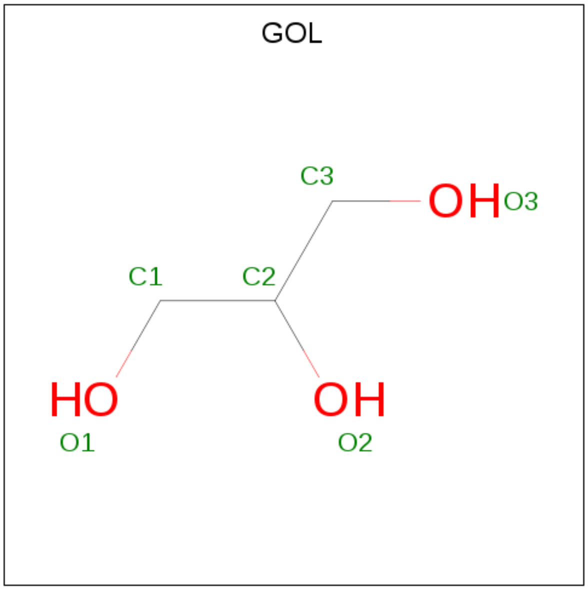

Mol	Chain	Residues	Atoms	ZeroOcc	AltConf
10	G	1	Total C O<br>6 3 3	0	0
10	G	1	Total C O<br>6 3 3	0	0
10	G	1	Total C O<br>6 3 3	0	0
10	G	1	Total C O<br>6 3 3	0	0
10	G	1	Total C O<br>6 3 3	0	0
10	G	1	Total C O<br>6 3 3	0	0

- Molecule 11 is 1,3-PROPANDIOL (three-letter code: PDO) (formula: $C_3H_8O_2$).

PRELIMINARY VALIDATION REPORT

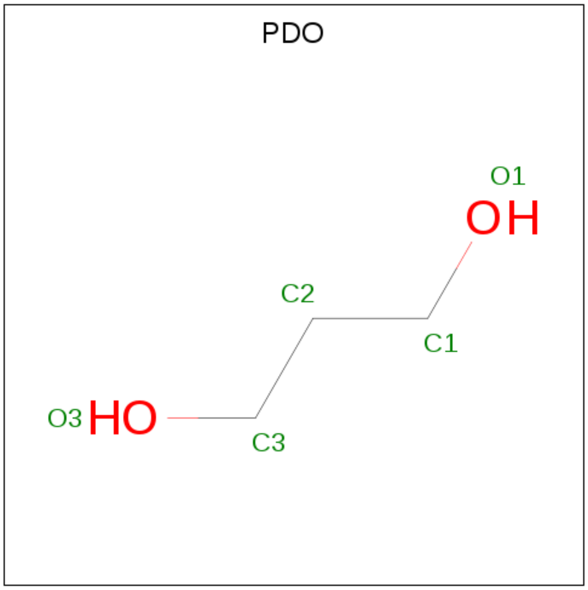

Mol	Chain	Residues	Atoms	ZeroOcc	AltConf
11	H	1	Total C O<br>5 3 2	0	0
11	H	1	Total C O<br>5 3 2	0	0
11	H	1	Total C O<br>5 3 2	0	0
11	H	1	Total C O<br>5 3 2	0	0
11	H	1	Total C O<br>5 3 2	0	0
11	H	1	Total C O<br>5 3 2	0	0
11	H	1	Total C O<br>5 3 2	0	0
11	H	1	Total C O<br>5 3 2	0	0
11	H	1	Total C O<br>5 3 2	0	0
11	H	1	Total C O<br>5 3 2	0	0
11	H	1	Total C O<br>5 3 2	0	0
11	H	1	Total C O<br>5 3 2	0	0
11	H	1	Total C O<br>5 3 2	0	0
11	H	1	Total C O<br>5 3 2	0	0

PRELIMINARY VALIDATION REPORT

- Molecule 12 is ETHANOL (three-letter code: EOH) (formula: C_2H_6O).

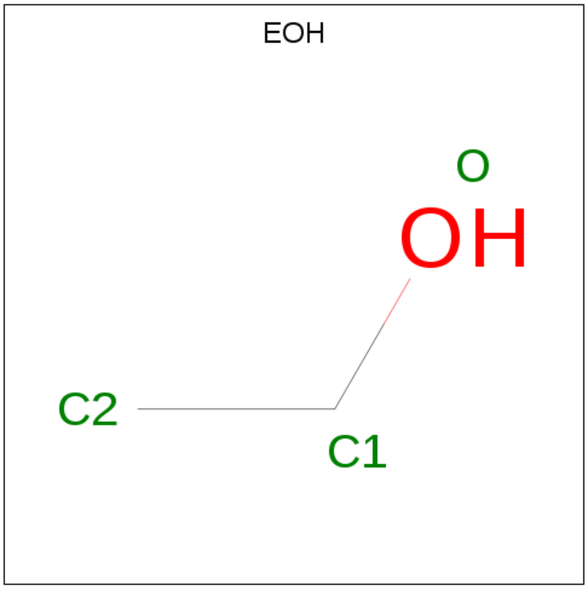

Mol	Chain	Residues	Atoms	ZeroOcc	AltConf
12	I	1	Total C O<br>3 2 1	0	0
12	I	1	Total C O<br>3 2 1	0	0
12	I	1	Total C O<br>3 2 1	0	0
12	I	1	Total C O<br>3 2 1	0	0
12	I	1	Total C O<br>3 2 1	0	0
12	I	1	Total C O<br>3 2 1	0	0
12	I	1	Total C O<br>3 2 1	0	0
12	I	1	Total C O<br>3 2 1	0	0
12	I	1	Total C O<br>3 2 1	0	0
12	I	1	Total C O<br>3 2 1	0	0
12	I	1	Total C O<br>3 2 1	0	0
12	I	1	Total C O<br>3 2 1	0	0
12	I	1	Total C O<br>3 2 1	0	0

Continued on next page...

PRELIMINARY VALIDATION REPORT

Continued from previous page...

Mol	Chain	Residues	Atoms	ZeroOcc	AltConf
12	I	1	Total C O<br>3 2 1	0	0
12	I	1	Total C O<br>3 2 1	0	0
12	I	1	Total C O<br>3 2 1	0	0
12	I	1	Total C O<br>3 2 1	0	0
12	I	1	Total C O<br>3 2 1	0	0
12	I	1	Total C O<br>3 2 1	0	0
12	I	1	Total C O<br>3 2 1	0	0
12	I	1	Total C O<br>3 2 1	0	0
12	I	1	Total C O<br>3 2 1	0	0
12	I	1	Total C O<br>3 2 1	0	0
12	I	1	Total C O<br>3 2 1	0	0
12	I	1	Total C O<br>3 2 1	0	0
12	I	1	Total C O<br>3 2 1	0	0
12	I	1	Total C O<br>3 2 1	0	0
12	I	1	Total C O<br>3 2 1	0	0
12	I	1	Total C O<br>3 2 1	0	0
12	I	1	Total C O<br>3 2 1	0	0
12	I	1	Total C O<br>3 2 1	0	0
12	I	1	Total C O<br>3 2 1	0	0
12	I	1	Total C O<br>3 2 1	0	0
12	I	1	Total C O<br>3 2 1	0	0

Continued on next page...

PRELIMINARY VALIDATION REPORT

Continued from previous page...

Mol	Chain	Residues	Atoms	ZeroOcc	AltConf
12	I	1	Total C O<br>3 2 1	0	0
12	I	1	Total C O<br>3 2 1	0	0
12	I	1	Total C O<br>3 2 1	0	0
12	I	1	Total C O<br>3 2 1	0	0
12	I	1	Total C O<br>3 2 1	0	0
12	I	1	Total C O<br>3 2 1	0	0
12	I	1	Total C O<br>3 2 1	0	0
12	I	1	Total C O<br>3 2 1	0	0
12	I	1	Total C O<br>3 2 1	0	0
12	I	1	Total C O<br>3 2 1	0	0
12	I	1	Total C O<br>3 2 1	0	0
12	I	1	Total C O<br>3 2 1	0	0
12	I	1	Total C O<br>3 2 1	0	0

- Molecule 13 is METHANOL (three-letter code: MOH) (formula: CH_4O).

PRELIMINARY VALIDATION REPORT

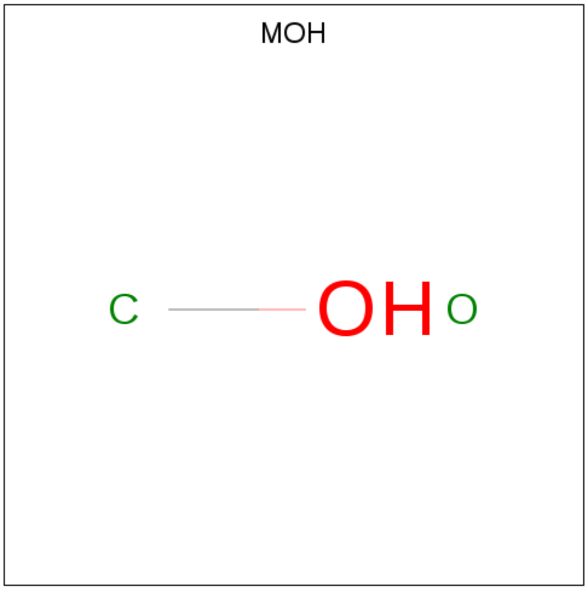

Mol	Chain	Residues	Atoms	ZeroOcc	AltConf
13	J	1	Total C O<br>2 1 1	0	0
13	J	1	Total C O<br>2 1 1	0	0
13	J	1	Total C O<br>2 1 1	0	0
13	J	1	Total C O<br>2 1 1	0	0
13	J	1	Total C O<br>2 1 1	0	0
13	J	1	Total C O<br>2 1 1	0	0
13	J	1	Total C O<br>2 1 1	0	0
13	J	1	Total C O<br>2 1 1	0	0
13	J	1	Total C O<br>2 1 1	0	0
13	J	1	Total C O<br>2 1 1	0	0
13	J	1	Total C O<br>2 1 1	0	0
13	J	1	Total C O<br>2 1 1	0	0
13	J	1	Total C O<br>2 1 1	0	0
13	J	1	Total C O<br>2 1 1	0	0

Continued on next page...

PRELIMINARY VALIDATION REPORT

Continued from previous page...

Mol	Chain	Residues	Atoms	ZeroOcc	AltConf
13	J	1	Total C O<br>2 1 1	0	0
13	J	1	Total C O<br>2 1 1	0	0
13	J	1	Total C O<br>2 1 1	0	0
13	J	1	Total C O<br>2 1 1	0	0
13	J	1	Total C O<br>2 1 1	0	0
13	J	1	Total C O<br>2 1 1	0	0
13	J	1	Total C O<br>2 1 1	0	0
13	J	1	Total C O<br>2 1 1	0	0
13	J	1	Total C O<br>2 1 1	0	0
13	J	1	Total C O<br>2 1 1	0	0
13	J	1	Total C O<br>2 1 1	0	0

- Molecule 14 is S-1,2-PROPANEDIOL (three-letter code: PGO) (formula: $C_3H_8O_2$).

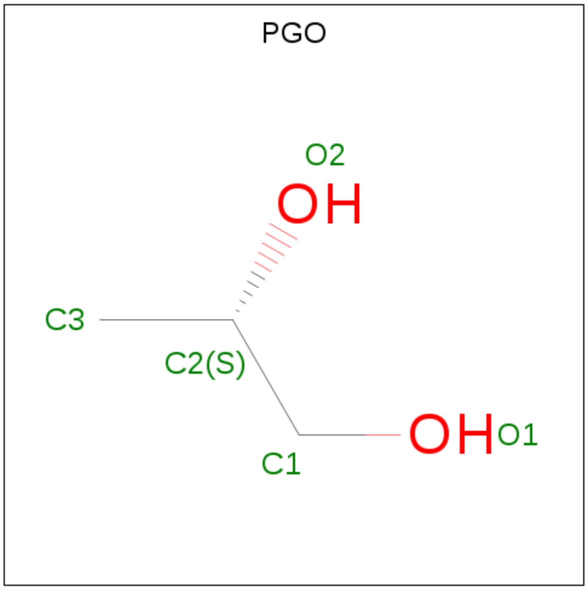

Mol	Chain	Residues	Atoms	ZeroOcc	AltConf
14	K	1	Total C O<br>5 3 2	0	0
14	K	1	Total C O<br>5 3 2	0	0
14	K	1	Total C O<br>5 3 2	0	0
14	K	1	Total C O<br>5 3 2	0	0
14	K	1	Total C O<br>5 3 2	0	0
14	K	1	Total C O<br>5 3 2	0	0
14	K	1	Total C O<br>5 3 2	0	0
14	K	1	Total C O<br>5 3 2	0	0
14	K	1	Total C O<br>5 3 2	0	0
14	K	1	Total C O<br>5 3 2	0	0

- Molecule 15 is TRIETHYLENE GLYCOL (three-letter code: PGE) (formula: $C_6H_{14}O_4$).

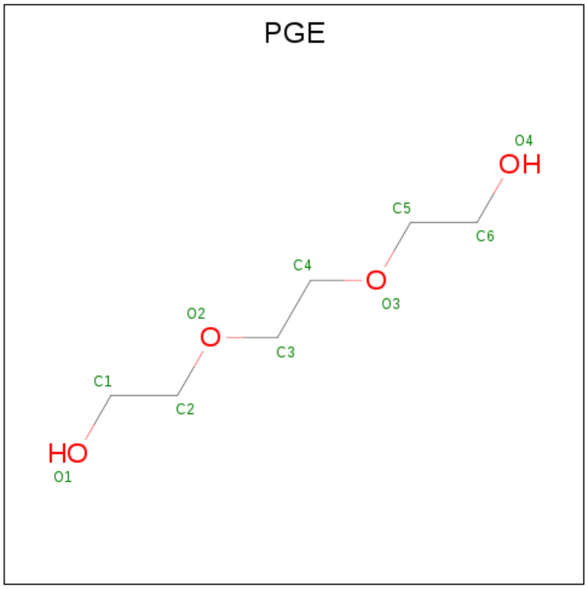

Mol	Chain	Residues	Atoms	ZeroOcc	AltConf
15	L	1	Total C O<br>10 6 4	0	0
15	L	1	Total C O<br>10 6 4	0	0

Continued on next page...

PRELIMINARY VALIDATION REPORT

Continued from previous page...

Mol	Chain	Residues	Atoms	ZeroOcc	AltConf
15	L	1	Total C O<br>10 6 4	0	0
15	L	1	Total C O<br>10 6 4	0	0
15	L	1	Total C O<br>10 6 4	0	0

- Molecule 16 is 2-ETHOXYETHANOL (three-letter code: ETX) (formula: $C_4H_{10}O_2$).

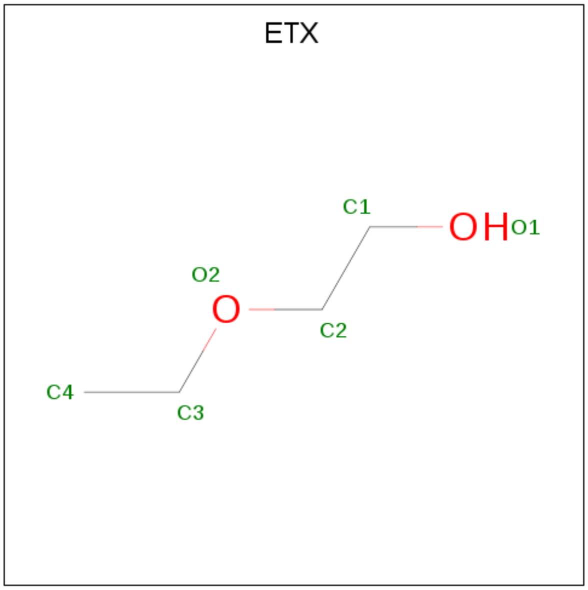

Mol	Chain	Residues	Atoms	ZeroOcc	AltConf
16	N	1	Total C O<br>6 4 2	0	0
16	N	1	Total C O<br>6 4 2	0	0
16	N	1	Total C O<br>6 4 2	0	0
16	N	1	Total C O<br>6 4 2	0	0
16	N	1	Total C O<br>6 4 2	0	0
16	N	1	Total C O<br>6 4 2	0	0
16	N	1	Total C O<br>6 4 2	0	0
16	N	1	Total C O<br>6 4 2	0	0

PRELIMINARY VALIDATION REPORT

- Molecule 17 is water.

Mol	Chain	Residues	Atoms		ZeroOcc	AltConf
			Total	O		
17	W	216	217	217	0	1

PRELIMINARY VALIDATION REPORT

3 Residue-property plots (i)

These plots are drawn for all protein, RNA and DNA chains in the entry. The first graphic for a chain summarises the proportions of the various outlier classes displayed in the second graphic. The second graphic shows the sequence view annotated by issues in geometry and electron density. Residues are color-coded according to the number of geometric quality criteria for which they contain at least one outlier: green = 0, yellow = 1, orange = 2 and red = 3 or more. A red dot above a residue indicates a poor fit to the electron density (RSRZ > 2). Stretches of 2 or more consecutive residues without any outlier are shown as a green connector. Residues present in the sample, but not in the model, are shown in grey.

- Molecule 1: Poxvirus entry fusion complex associated protein F9

Chain A:

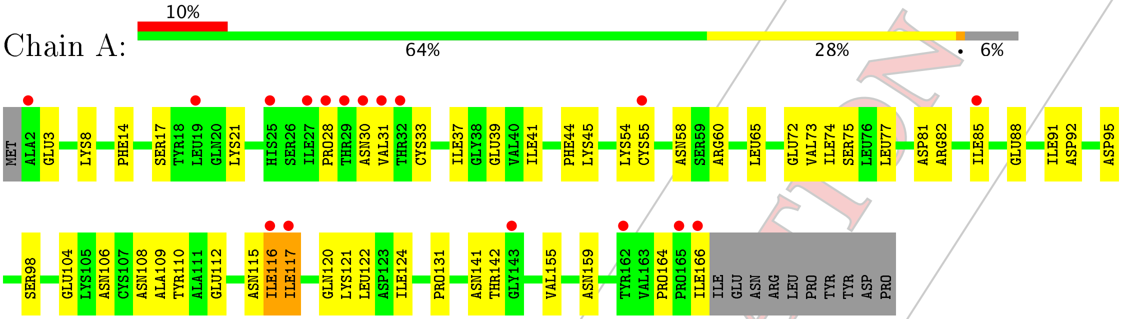

- Molecule 1: Poxvirus entry fusion complex associated protein F9

Chain B:

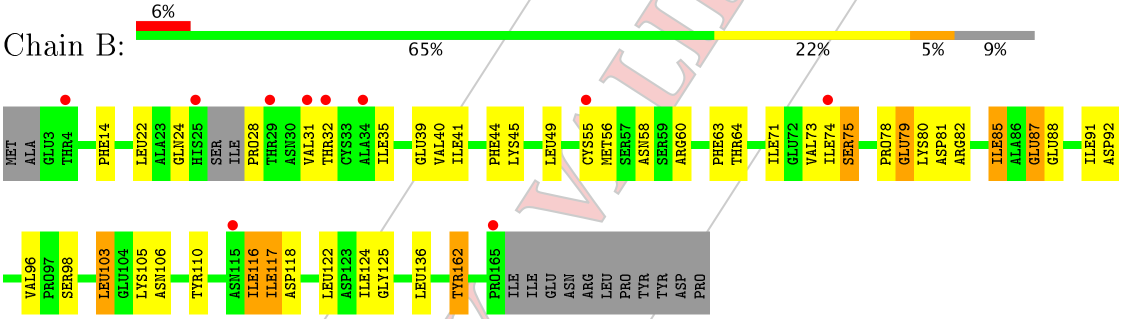

- Molecule 1: Poxvirus entry fusion complex associated protein F9

Chain C:

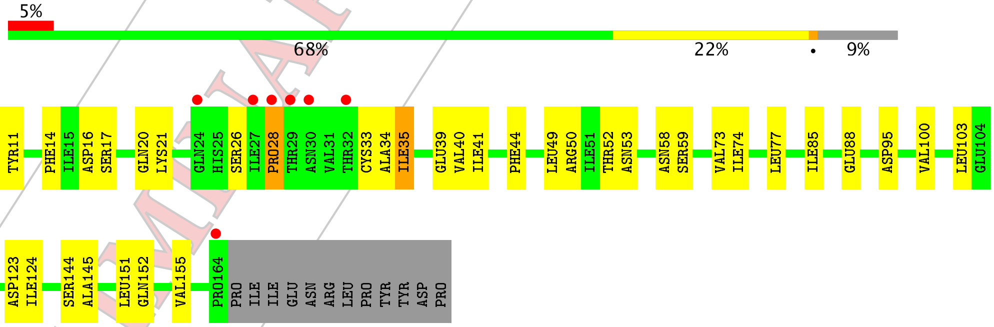

- Molecule 2:

Chain D:

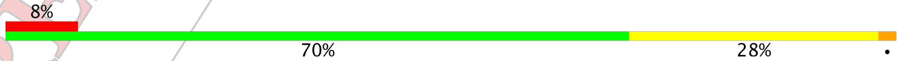

PRELIMINARY VALIDATION REPORT

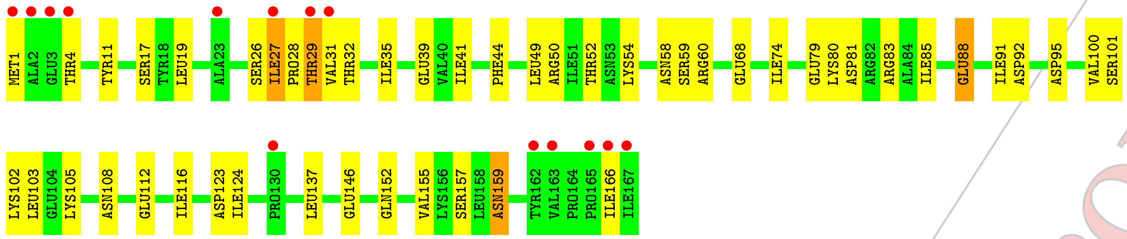

PRELIMINARY VALIDATION REPORT

4 Data and refinement statistics (i)

Property	Value	Source
Space group	P 21 21 21	Depositor
Cell constants<br>a, b, c, α, β, γ	65.45Å 75.08Å 136.25Å<br>90.00° 90.00° 90.00°	Depositor
Resolution (Å)	38.86 – 2.10<br>39.96 – 2.10	Depositor<br>EDS
% Data completeness<br>(in resolution range)	93.8 (38.86-2.10)<br>93.9 (39.96-2.10)	Depositor<br>EDS
R_{merge}	(Not available)	Depositor
R_{sym}	(Not available)	Depositor
$< I/\sigma(I) >$ [1]	2.20 (at 2.10Å)	Xtriage
Refinement program	PHENIX (1.12_2829: ???)	Depositor
R, R_{free}	0.226 , 0.269<br>0.234 , 0.281	Depositor<br>DCC
R_{free} test set	2000 reflections (5.63%)	DCC
Wilson B-factor (Å^2)	30.5	Xtriage
Anisotropy	0.854	Xtriage
Bulk solvent k_{sol}(e/Å^3), B_{sol}(Å^2)	0.33 , 67.9	EDS
L-test for twinning[2]	$< \|L\| >$ = 0.46, $< L^2 >$ = 0.29	Xtriage
Estimated twinning fraction	No twinning to report.	Xtriage
F_o,F_c correlation	0.94	EDS
Total number of atoms	6062	wwPDB-VP
Average B, all atoms (Å^2)	42.0	wwPDB-VP

Xtriage's analysis on translational NCS is as follows: *The analyses of the Patterson function reveals a significant off-origin peak that is 31.08 % of the origin peak, indicating pseudo translational symmetry. The chance of finding a peak of this or larger height randomly in a structure without pseudo translational symmetry is equal to 1.1740e-03. The detected translational NCS is most likely also responsible for the elevated intensity ratio.*

[1] Intensities estimated from amplitudes.
[2] Theoretical values of $< |L| >$, $< L^2 >$ for acentric reflections are 0.5, 0.333 respectively for untwinned datasets, and 0.375, 0.2 for perfectly twinned datasets.

PRELIMINARY VALIDATION REPORT

5 Model quality (i)

5.1 Standard geometry (i)

Bond lengths and bond angles in the following residue types are not validated in this section: GOL, PGE, P6G, PGO, ETX, EOH, EDO, PDO, PG4, MOH, PG0, ETE, P33, PEG

The Z score for a bond length (or angle) is the number of standard deviations the observed value is removed from the expected value. A bond length (or angle) with $|Z| > 5$ is considered an outlier worth inspection. RMSZ is the root-mean-square of all Z scores of the bond lengths (or angles).

Mol	Chain	Bond lengths		Bond angles	
		RMSZ	$\#|Z| >5$	RMSZ	$\#|Z| >5$
1	A	0.32	0/1257	0.44	0/1706
1	B	0.39	1/1226 (0.1%)	0.51	1/1659 (0.1%)
1	C	0.34	0/1240	0.49	1/1679 (0.1%)
2	D	0.28	0/1287	0.48	0/1742
All	All	0.33	1/5010 (0.0%)	0.48	2/6786 (0.0%)

All (1) bond length outliers are listed below:

Mol	Chain	Res	Type	Atoms	Z	Observed(Å)	Ideal(Å)
1	B	162	TYR	CB-CG	-6.29	1.42	1.51

All (2) bond angle outliers are listed below:

Mol	Chain	Res	Type	Atoms	Z	Observed(^{o})	Ideal(^{o})
1	C	28	PRO	N-CA-CB	6.10	110.62	103.30
1	B	28	PRO	N-CA-CB	5.91	110.39	103.30

There are no chirality outliers.

There are no planarity outliers.

5.2 Too-close contacts (i)

In the following table, the Non-H and H(model) columns list the number of non-hydrogen atoms and hydrogen atoms in the chain respectively. The H(added) column lists the number of hydrogen atoms added and optimized by MolProbity. The Clashes column lists the number of clashes within the asymmetric unit, whereas Symm-Clashes lists symmetry related clashes.

Mol	Chain	Non-H	H(model)	H(added)	Clashes	Symm-Clashes
1	A	1239	0	1217	49	0

Continued on next page...

PRELIMINARY VALIDATION REPORT

Continued from previous page...

Mol	Chain	Non-H	H(model)	H(added)	Clashes	Symm-Clashes
1	B	1211	0	1189	34	0
1	C	1226	0	1196	41	0
2	D	1271	0	1264	65	0
3	E	65	0	88	12	0
4	P	72	0	108	32	0
5	M	77	0	109	29	0
6	Q	57	0	78	22	0
7	R	22	0	30	14	0
8	S	28	0	40	5	0
9	F	132	0	188	21	0
10	G	36	0	41	4	0
11	H	70	0	110	26	0
12	I	141	0	276	35	0
13	J	50	0	0	4	0
14	K	50	0	76	22	0
15	L	50	0	69	12	0
16	N	48	0	79	27	0
17	W	217	0	0	11	0
All	All	6062	0	6158	280	0

The all-atom clashscore is defined as the number of clashes found per 1000 atoms (including hydrogen atoms). The all-atom clashscore for this structure is 23.

All (280) close contacts within the same asymmetric unit are listed below, sorted by their clash magnitude.

Atom-1	Atom-2	Interatomic distance (Å)	Clash overlap (Å)
2:D:101:SER:HA	11:H:10:PDO:H32	1.21	1.16
5:M:6:PEG:H11	11:H:1:PDO:H31	1.22	1.15
14:K:5:PGO:C3	16:N:4:ETX:H12	1.78	1.12
8:S:1:ETE:H141	16:N:2:ETX:H41	1.20	1.11
14:K:5:PGO:H31	16:N:4:ETX:H12	1.17	1.09
4:P:2:PG0:H42	17:W:49:HOH:O	1.56	1.05
7:R:1:P33:H202	7:R:1:P33:H152	1.33	1.04
6:Q:2:P6G:H142	6:Q:2:P6G:H181	1.38	1.03
7:R:1:P33:H171	7:R:1:P33:H212	1.38	1.03
6:Q:2:P6G:H112	6:Q:2:P6G:H51	1.40	1.03
9:F:3:EDO:H21	10:G:2:GOL:H11	1.42	1.01
2:D:100:VAL:HG11	3:E:4:PG4:H32	1.41	1.00
14:K:5:PGO:H31	16:N:4:ETX:C1	1.93	0.99
5:M:6:PEG:C1	11:H:1:PDO:H31	1.93	0.98
3:E:1:PG4:H41	3:E:1:PG4:H12	1.45	0.98

Continued on next page...

PRELIMINARY VALIDATION REPORT

Continued from previous page...

Atom-1	Atom-2	Interatomic distance (Å)	Clash overlap (Å)
5:M:6:PEG:H11	11:H:1:PDO:C3	1.93	0.97
1:A:108:ASN:HB3	11:H:6:PDO:H12	1.45	0.97
2:D:17:SER:HA	5:M:2:PEG:H21	1.46	0.96
5:M:1:PEG:H32	9:F:9:EDO:H12	1.47	0.94
2:D:101:SER:CA	11:H:10:PDO:H32	1.98	0.93
8:S:1:ETE:C14	16:N:2:ETX:H41	1.99	0.92
3:E:3:PG4:C3	3:E:4:PG4:H82	2.00	0.92
4:P:5:PG0:H51	4:P:7:PG0:C5	2.03	0.89
6:Q:2:P6G:H151	6:Q:2:P6G:C5	2.04	0.87
6:Q:2:P6G:H151	6:Q:2:P6G:H51	1.56	0.87
1:C:144:SER:HA	5:M:1:PEG:H42	1.55	0.86
8:S:1:ETE:H141	16:N:2:ETX:C4	2.02	0.86
4:P:4:PG0:H21	5:M:2:PEG:H12	1.57	0.85
2:D:101:SER:HA	11:H:10:PDO:C3	2.05	0.84
5:M:7:PEG:H41	12:I:28:EOH:H12	1.59	0.83
6:Q:2:P6G:C14	6:Q:2:P6G:H181	2.02	0.83
7:R:1:P33:H202	7:R:1:P33:C15	2.08	0.83
12:I:13:EOH:H11	16:N:1:ETX:O2	1.80	0.82
14:K:5:PGO:C3	16:N:4:ETX:C1	2.55	0.82
1:A:88:GLU:HA	4:P:9:PG0:H11	1.62	0.81
1:A:33:CYS:SG	9:F:13:EDO:H22	2.21	0.81
11:H:9:PDO:H31	12:I:5:EOH:H11	1.64	0.80
2:D:92:ASP:H	11:H:10:PDO:H22	1.48	0.79
2:D:92:ASP:H	11:H:10:PDO:C2	1.97	0.78
1:C:53:ASN:HB2	15:L:5:PGE:H2	1.63	0.78
1:C:5:LYS:HE3	12:I:29:EOH:H21	1.66	0.78
1:A:120:GLN:HA	4:P:6:PG0:H12	1.65	0.77
12:I:13:EOH:C1	16:N:1:ETX:H12	2.15	0.77
1:C:33:CYS:SG	15:L:5:PGE:H42	2.25	0.77
3:E:3:PG4:H31	3:E:4:PG4:H82	1.66	0.76
1:A:8:LYS:NZ	14:K:5:PGO:H2	2.00	0.76
1:A:3:GLU:HB3	5:M:6:PEG:H41	1.69	0.74
12:I:13:EOH:H21	16:N:1:ETX:C1	2.17	0.74
1:C:52:THR:HG21	9:F:5:EDO:H11	1.68	0.74
6:Q:2:P6G:C11	6:Q:2:P6G:H51	2.15	0.74
12:I:13:EOH:H21	16:N:1:ETX:H12	1.69	0.74
7:R:1:P33:H171	7:R:1:P33:C21	2.05	0.73
4:P:4:PG0:H21	5:M:2:PEG:C1	2.20	0.72
1:C:88:GLU:HG2	9:F:6:EDO:O2	1.90	0.72
9:F:3:EDO:H21	10:G:2:GOL:C1	2.18	0.72
2:D:27:ILE:HG23	14:K:9:PGO:H12	1.71	0.72

Continued on next page...

PRELIMINARY VALIDATION REPORT

Continued from previous page...

Atom-1	Atom-2	Interatomic distance (Å)	Clash overlap (Å)
4:P:9:PG0:C5	4:P:9:PG0:H22	2.20	0.71
1:B:75:SER:HA	1:B:82:ARG:HE	1.54	0.70
2:D:88:GLU:HB2	6:Q:3:P6G:H92	1.74	0.70
4:P:9:PG0:H22	4:P:9:PG0:O2	1.90	0.70
1:B:96:VAL:HG21	12:I:33:EOH:H21	1.74	0.70
4:P:4:PG0:H11	12:I:13:EOH:H12	1.73	0.69
6:Q:2:P6G:H151	6:Q:2:P6G:H112	1.73	0.69
1:C:95:ASP:OD2	11:H:7:PDO:H12	1.93	0.68
1:A:108:ASN:HB2	11:H:6:PDO:H32	1.74	0.68
1:B:125:GLY:HA2	4:P:1:PG0:H11	1.76	0.67
4:P:5:PG0:H51	4:P:7:PG0:H52	1.76	0.67
2:D:31:VAL:HG12	2:D:32:THR:HG23	1.77	0.67
1:A:109:ALA:N	11:H:6:PDO:H31	2.09	0.66
1:A:121:LYS:H	4:P:6:PG0:H22	1.60	0.66
2:D:54:LYS:HE3	14:K:4:PGO:H2	1.77	0.66
2:D:88:GLU:HB2	6:Q:3:P6G:C9	2.26	0.65
2:D:44:PHE:HE1	2:D:124:ILE:HD11	1.61	0.65
2:D:54:LYS:NZ	5:M:4:PEG:O1	2.25	0.65
2:D:17:SER:CA	5:M:2:PEG:H21	2.24	0.65
6:Q:2:P6G:H112	6:Q:2:P6G:C5	2.23	0.65
12:I:13:EOH:C2	16:N:1:ETX:H12	2.26	0.65
6:Q:2:P6G:C18	6:Q:2:P6G:H142	2.21	0.65
2:D:35:ILE:HD11	2:D:116:ILE:HG22	1.78	0.65
4:P:1:PG0:C3	9:F:10:EDO:H12	2.27	0.65
1:B:39:GLU:HG3	2:D:41:ILE:HG22	1.77	0.65
1:A:75:SER:HA	1:A:82:ARG:HD3	1.79	0.64
4:P:5:PG0:H51	4:P:7:PG0:H53	1.78	0.64
12:I:16:EOH:H23	17:W:102:HOH:O	1.96	0.64
14:K:5:PGO:H32	16:N:4:ETX:H12	1.75	0.63
1:B:40:VAL:HG11	1:B:49:LEU:HD21	1.79	0.63
2:D:105:LYS:HE2	3:E:4:PG4:H22	1.80	0.63
1:B:60:ARG:HG3	16:N:8:ETX:H12	1.82	0.62
1:B:87:GLU:CG	9:F:14:EDO:H21	2.30	0.61
1:A:108:ASN:CB	11:H:6:PDO:H32	2.31	0.61
1:B:32:THR:CA	11:H:2:PDO:H21	2.31	0.60
3:E:3:PG4:O2	3:E:4:PG4:H82	2.01	0.60
1:C:10:LEU:HD21	1:C:77:LEU:HD11	1.82	0.60
1:C:145:ALA:H	5:M:1:PEG:H42	1.67	0.60
2:D:112:GLU:OE1	17:W:58:HOH:O	2.17	0.60
5:M:7:PEG:H32	10:G:6:GOL:H12	1.82	0.59
1:C:20:GLN:HB3	7:R:1:P33:H31	1.85	0.59

Continued on next page...

PRELIMINARY VALIDATION REPORT

Continued from previous page...

Atom-1	Atom-2	Interatomic distance (Å)	Clash overlap (Å)
14:K:5:PGO:C3	16:N:4:ETX:C2	2.81	0.59
1:C:100:VAL:HB	5:M:5:PEG:H41	1.85	0.58
1:A:60:ARG:NH2	1:A:98:SER:O	2.36	0.58
2:D:19:LEU:HD22	2:D:54:LYS:HZ3	1.67	0.58
13:J:21:MOH:C	16:N:6:ETX:H42	2.34	0.58
2:D:19:LEU:HD13	5:M:4:PEG:H22	1.85	0.58
5:M:7:PEG:H41	12:I:28:EOH:C1	2.32	0.57
1:A:166:ILE:N	17:W:193:HOH:O	2.38	0.57
1:C:5:LYS:HD2	12:I:10:EOH:H11	1.87	0.57
2:D:52:THR:CG2	5:M:4:PEG:H41	2.35	0.57
1:C:144:SER:HB3	5:M:1:PEG:H21	1.87	0.57
4:P:8:PG0:H12	9:F:27:EDO:O1	2.06	0.56
1:A:115:ASN:O	1:A:117:ILE:N	2.38	0.56
1:A:17:SER:CB	11:H:3:PDO:H21	2.36	0.56
1:C:16:ASP:OD2	4:P:3:PG0:H31	2.06	0.56
2:D:95:ASP:CB	11:H:10:PDO:H11	2.36	0.56
1:C:112:GLU:OE2	9:F:33:EDO:H12	2.06	0.56
1:B:24:GLN:CG	1:B:24:GLN:O	2.54	0.55
1:A:91:ILE:HG23	12:I:43:EOH:H11	1.86	0.55
1:C:40:VAL:HG11	1:C:49:LEU:HD21	1.87	0.55
16:N:1:ETX:H43	16:N:2:ETX:H22	1.88	0.55
1:B:64:THR:HG21	15:L:4:PGE:H5	1.87	0.55
2:D:52:THR:HG23	5:M:4:PEG:H41	1.88	0.55
1:C:144:SER:CA	5:M:1:PEG:H42	2.35	0.55
1:A:41:ILE:HG22	1:C:39:GLU:HG3	1.89	0.55
4:P:9:PG0:H52	4:P:9:PG0:H22	1.89	0.55
1:B:162:TYR:HB2	9:F:19:EDO:H11	1.90	0.54
4:P:4:PG0:C2	5:M:2:PEG:C1	2.84	0.54
1:C:105:LYS:HB3	14:K:1:PGO:H12	1.89	0.54
1:C:144:SER:HA	5:M:1:PEG:C4	2.35	0.54
2:D:74:ILE:HD11	2:D:85:ILE:HG21	1.89	0.53
7:R:1:P33:H61	15:L:2:PGE:O2	2.08	0.53
6:Q:2:P6G:H112	6:Q:2:P6G:O7	2.02	0.53
1:B:22:LEU:HD22	1:B:56:MET:HE1	1.91	0.53
1:A:8:LYS:HZ3	14:K:5:PGO:H2	1.72	0.53
1:A:39:GLU:HG3	1:C:41:ILE:HG22	1.90	0.53
1:B:87:GLU:HG3	9:F:14:EDO:H21	1.90	0.53
4:P:3:PG0:H32	12:I:16:EOH:H11	1.91	0.53
3:E:4:PG4:O2	4:P:7:PG0:H31	2.08	0.53
4:P:3:PG0:H31	12:I:16:EOH:H21	1.91	0.52
1:B:92:ASP:HB3	12:I:15:EOH:H21	1.91	0.52

Continued on next page...

PRELIMINARY VALIDATION REPORT

Continued from previous page...

Atom-1	Atom-2	Interatomic distance (Å)	Clash overlap (Å)
1:A:106:ASN:ND2	17:W:7:HOH:O	2.30	0.52
1:B:87:GLU:HG2	9:F:14:EDO:H21	1.90	0.52
1:C:11:TYR:CZ	1:C:155:VAL:HG22	2.46	0.51
12:I:16:EOH:C2	17:W:102:HOH:O	2.57	0.51
6:Q:2:P6G:H181	6:Q:2:P6G:H122	1.92	0.51
2:D:54:LYS:HE3	14:K:4:PGO:C2	2.41	0.51
2:D:54:LYS:HG2	14:K:4:PGO:C2	2.41	0.51
12:I:13:EOH:C1	16:N:1:ETX:C1	2.85	0.51
2:D:108:ASN:CG	6:Q:1:P6G:H141	2.31	0.51
1:A:120:GLN:HG3	4:P:6:PG0:C1	2.40	0.51
1:A:121:LYS:HE3	4:P:6:PG0:H32	1.93	0.51
16:N:1:ETX:C4	16:N:2:ETX:H22	2.41	0.51
12:I:13:EOH:C2	16:N:1:ETX:C1	2.85	0.50
9:F:7:EDO:O1	12:I:5:EOH:H23	2.12	0.50
6:Q:2:P6G:H151	6:Q:2:P6G:C11	2.40	0.50
2:D:100:VAL:HG11	3:E:4:PG4:C3	2.28	0.50
5:M:7:PEG:C4	12:I:28:EOH:H12	2.37	0.50
1:B:96:VAL:HG21	12:I:33:EOH:C2	2.40	0.50
1:B:136:LEU:HD21	2:D:1:MET:HE3	1.94	0.49
1:A:155:VAL:O	1:A:159:ASN:HB2	2.12	0.49
1:C:35:ILE:HD11	1:C:116:ILE:HG22	1.93	0.49
2:D:101:SER:CB	11:H:10:PDO:H32	2.41	0.49
1:A:17:SER:OG	11:H:3:PDO:H21	2.11	0.49
1:A:95:ASP:HB2	12:I:43:EOH:H23	1.93	0.49
8:S:2:ETE:H252	12:I:40:EOH:H23	1.93	0.49
1:B:81:ASP:O	1:B:85:ILE:HG22	2.13	0.49
2:D:29:THR:O	2:D:29:THR:OG1	2.20	0.49
6:Q:2:P6G:C15	6:Q:2:P6G:C5	2.85	0.49
4:P:7:PG0:H42	9:F:28:EDO:C2	2.43	0.49
7:R:1:P33:H61	15:L:2:PGE:C3	2.43	0.49
1:A:55:CYS:HA	1:A:142:THR:HA	1.95	0.49
12:I:13:EOH:H21	16:N:1:ETX:H11	1.92	0.49
1:C:17:SER:HB2	7:R:1:P33:H92	1.93	0.49
2:D:58:ASN:HD22	2:D:60:ARG:HB2	1.77	0.48
1:C:14:PHE:HA	1:C:73:VAL:HG21	1.95	0.48
1:C:74:ILE:HD11	1:C:85:ILE:HG21	1.95	0.48
12:I:13:EOH:H11	16:N:1:ETX:C1	2.43	0.48
5:M:1:PEG:H32	9:F:9:EDO:C1	2.30	0.48
1:B:41:ILE:HG12	2:D:39:GLU:HG3	1.96	0.48
1:C:16:ASP:OD2	4:P:3:PG0:H21	2.14	0.48
14:K:5:PGO:C3	16:N:4:ETX:H22	2.44	0.48

Continued on next page...

PRELIMINARY VALIDATION REPORT

Continued from previous page...

Atom-1	Atom-2	Interatomic distance (Å)	Clash overlap (Å)
2:D:92:ASP:H	11:H:10:PDO:H21	1.74	0.48
1:C:44:PHE:HE1	1:C:124:ILE:HD11	1.79	0.47
1:C:77:LEU:O	9:F:25:EDO:O2	2.32	0.47
2:D:68:GLU:HA	13:J:6:MOH:C	2.44	0.47
8:S:1:ETE:H141	16:N:2:ETX:C3	2.42	0.47
1:B:79:GLU:CB	12:I:19:EOH:H12	2.44	0.47
2:D:100:VAL:CG1	3:E:4:PG4:H32	2.30	0.47
1:C:34:ALA:HA	9:F:1:EDO:H22	1.95	0.47
2:D:17:SER:OG	5:M:2:PEG:H41	2.14	0.47
7:R:1:P33:H182	7:R:1:P33:H151	1.60	0.47
2:D:124:ILE:HD12	2:D:137:LEU:HD23	1.97	0.47
1:A:121:LYS:N	4:P:6:PG0:H22	2.28	0.47
1:C:44:PHE:CE1	1:C:124:ILE:HD11	2.50	0.47
2:D:19:LEU:HD13	5:M:4:PEG:C2	2.45	0.46
2:D:80:LYS:HA	13:J:10:MOH:O	2.15	0.46
1:A:44:PHE:HE1	1:A:124:ILE:HD11	1.80	0.46
1:B:106:ASN:ND2	17:W:93:HOH:O	2.44	0.46
1:C:52:THR:CG2	9:F:5:EDO:H11	2.41	0.46
15:L:2:PGE:H32	15:L:2:PGE:H1	1.55	0.46
1:A:122:LEU:HD12	1:A:122:LEU:HA	1.83	0.46
12:I:25:EOH:H22	12:I:26:EOH:H21	1.95	0.46
6:Q:2:P6G:H181	6:Q:2:P6G:C12	2.46	0.46
1:A:121:LYS:H	4:P:6:PG0:C2	2.25	0.46
2:D:85:ILE:HD11	2:D:157:SER:HB3	1.96	0.46
1:A:60:ARG:HD2	1:A:104:GLU:OE2	2.15	0.46
2:D:92:ASP:HB3	11:H:10:PDO:H22	1.98	0.46
1:B:117:ILE:HG21	12:I:39:EOH:H21	1.98	0.46
1:C:11:TYR:CE1	1:C:155:VAL:HG22	2.51	0.46
1:A:81:ASP:O	1:A:85:ILE:HG22	2.16	0.45
1:B:91:ILE:HG23	12:I:15:EOH:H12	1.98	0.45
4:P:9:PG0:C2	4:P:9:PG0:H52	2.46	0.45
2:D:54:LYS:HG2	14:K:4:PGO:O2	2.17	0.45
4:P:3:PG0:H12	17:W:210:HOH:O	2.17	0.45
2:D:26:SER:O	2:D:28:PRO:HD3	2.16	0.45
1:A:58:ASN:CB	9:F:30:EDO:H21	2.47	0.45
1:B:64:THR:CG2	15:L:4:PGE:H5	2.47	0.45
1:B:79:GLU:H	12:I:19:EOH:H12	1.81	0.45
1:A:112:GLU:HG3	11:H:6:PDO:H11	1.99	0.45
1:B:105:LYS:HB3	14:K:1:PGO:H31	1.98	0.45
6:Q:3:P6G:H182	15:L:1:PGE:H6	1.98	0.44
2:D:11:TYR:CZ	2:D:155:VAL:HG22	2.51	0.44

Continued on next page...

PRELIMINARY VALIDATION REPORT

Continued from previous page...

Atom-1	Atom-2	Interatomic distance (Å)	Clash overlap (Å)
3:E:1:PG4:H12	3:E:1:PG4:C4	2.18	0.44
7:R:1:P33:H81	15:L:2:PGE:H2	2.00	0.44
1:A:44:PHE:CE1	1:A:124:ILE:HD11	2.53	0.44
2:D:91:ILE:HA	11:H:10:PDO:H21	2.00	0.44
14:K:5:PGO:H33	16:N:4:ETX:H22	1.99	0.44
2:D:26:SER:O	14:K:9:PGO:H11	2.18	0.44
1:C:17:SER:N	7:R:1:P33:H91	2.33	0.44
1:B:98:SER:HB3	15:L:4:PGE:H6	1.99	0.44
2:D:50:ARG:CB	5:M:4:PEG:H42	2.48	0.44
1:A:164:PRO:HB2	12:I:35:EOH:H12	1.99	0.43
1:C:123:ASP:O	1:C:152:GLN:HG3	2.18	0.43
2:D:155:VAL:O	2:D:159:ASN:HB2	2.18	0.43
1:B:14:PHE:HA	1:B:73:VAL:HG21	2.00	0.43
6:Q:2:P6G:H151	6:Q:2:P6G:H52	1.97	0.43
1:B:63:PHE:HE1	1:B:103:LEU:HD13	1.83	0.43
1:C:112:GLU:HA	4:P:2:PG0:H21	2.01	0.43
2:D:95:ASP:HB2	11:H:10:PDO:H11	2.00	0.43
1:B:58:ASN:HB2	17:W:206:HOH:O	2.17	0.43
1:A:109:ALA:CA	11:H:6:PDO:H31	2.48	0.43
1:A:131:PRO:HB2	3:E:5:PG4:H52	2.01	0.43
15:L:3:PGE:H4	15:L:3:PGE:H22	1.47	0.43
7:R:1:P33:C6	15:L:2:PGE:O2	2.67	0.43
1:C:21:LYS:HG3	7:R:1:P33:H32	2.00	0.43
1:A:77:LEU:O	1:A:82:ARG:NE	2.51	0.42
2:D:146:GLU:H	2:D:146:GLU:CD	2.23	0.42
2:D:27:ILE:CG2	14:K:9:PGO:H12	2.45	0.42
4:P:2:PG0:H52	17:W:49:HOH:O	2.18	0.42
1:A:120:GLN:CA	4:P:6:PG0:H12	2.43	0.42
2:D:102:LYS:NZ	11:H:6:PDO:H22	2.34	0.42
2:D:123:ASP:O	2:D:152:GLN:HG3	2.20	0.42
2:D:81:ASP:O	2:D:85:ILE:HG22	2.20	0.42
3:E:3:PG4:C4	3:E:4:PG4:H82	2.47	0.42
1:B:78:PRO:O	1:B:82:ARG:HG3	2.20	0.42
2:D:74:ILE:HA	2:D:74:ILE:HD12	1.89	0.42
1:A:92:ASP:HB2	14:K:8:PGO:C1	2.50	0.42
12:I:13:EOH:H11	16:N:1:ETX:H12	2.00	0.42
1:A:45:LYS:O	1:C:52:THR:HA	2.20	0.41
1:A:21:LYS:HB3	1:A:65:LEU:HD13	2.02	0.41
1:B:45:LYS:O	2:D:52:THR:HA	2.21	0.41
2:D:27:ILE:HG23	14:K:9:PGO:C1	2.45	0.41
1:A:37:ILE:HG12	1:A:141:ASN:HB2	2.02	0.41

Continued on next page...

PRELIMINARY VALIDATION REPORT

Continued from previous page...

Atom-1	Atom-2	Interatomic distance (Å)	Clash overlap (Å)
2:D:88:GLU:HB2	6:Q:3:P6G:H91	2.01	0.41
1:A:41:ILE:HD13	1:A:121:LYS:HD3	2.03	0.41
1:A:58:ASN:HB2	9:F:30:EDO:H21	2.01	0.41
1:C:59:SER:OG	10:G:3:GOL:H32	2.21	0.41
2:D:59:SER:OG	6:Q:1:P6G:H171	2.21	0.41
1:C:151:LEU:O	1:C:155:VAL:HG23	2.20	0.41
1:A:72:GLU:HG2	17:W:113:HOH:O	2.21	0.41
1:A:74:ILE:HD11	1:A:85:ILE:HG21	2.02	0.41
2:D:79:GLU:HG2	2:D:83[B]:ARG:NH1	2.36	0.41
2:D:137:LEU:HD11	2:D:159:ASN:OD1	2.21	0.41
2:D:26:SER:N	14:K:9:PGO:H2	2.36	0.41
1:A:14:PHE:HA	1:A:73:VAL:HG21	2.03	0.40
2:D:102:LYS:HZ2	12:I:1:EOH:H21	1.86	0.40
1:B:71:ILE:HA	1:B:74:ILE:HG22	2.03	0.40
1:C:145:ALA:N	5:M:1:PEG:H42	2.34	0.40
7:R:1:P33:H21	7:R:1:P33:H52	1.57	0.40
1:B:44:PHE:CE1	1:B:124:ILE:HD11	2.57	0.40
13:J:21:MOH:C	16:N:6:ETX:C4	3.00	0.40
6:Q:3:P6G:H112	6:Q:3:P6G:H142	1.33	0.40

There are no symmetry-related clashes.

5.3 Torsion angles (i)

5.3.1 Protein backbone (i)

In the following table, the Percentiles column shows the percent Ramachandran outliers of the chain as a percentile score with respect to all X-ray entries followed by that with respect to entries of similar resolution.

The Analysed column shows the number of residues for which the backbone conformation was analysed, and the total number of residues.

Mol	Chain	Analysed	Favoured	Allowed	Outliers	Percentiles	
1	A	163/176 (93%)	151 (93%)	10 (6%)	2 (1%)	15	9
1	B	157/176 (89%)	144 (92%)	9 (6%)	4 (2%)	6	2
1	C	160/176 (91%)	149 (93%)	10 (6%)	1 (1%)	28	24
2	D	163/166 (98%)	153 (94%)	9 (6%)	1 (1%)	28	24
All	All	643/694 (93%)	597 (93%)	38 (6%)	8 (1%)	15	9

PRELIMINARY VALIDATION REPORT

All (8) Ramachandran outliers are listed below:

Mol	Chain	Res	Type
1	A	116	ILE
1	B	117	ILE
1	A	28	PRO
1	B	116	ILE
1	C	28	PRO
1	B	31	VAL
1	B	79	GLU
2	D	166	ILE

5.3.2 Protein sidechains (i)

In the following table, the Percentiles column shows the percent sidechain outliers of the chain as a percentile score with respect to all X-ray entries followed by that with respect to entries of similar resolution.

The Analysed column shows the number of residues for which the sidechain conformation was analysed, and the total number of residues.

Mol	Chain	Analysed	Rotameric	Outliers	Percentiles	
1	A	135/157 (86%)	129 (96%)	6 (4%)	33	31
1	B	132/157 (84%)	120 (91%)	12 (9%)	11	7
1	C	134/157 (85%)	129 (96%)	5 (4%)	39	39
2	D	141/147 (96%)	134 (95%)	7 (5%)	28	26
All	All	542/618 (88%)	512 (94%)	30 (6%)	25	22

All (30) residues with a non-rotameric sidechain are listed below:

Mol	Chain	Res	Type
1	A	30	ASN
1	A	31	VAL
1	A	54	LYS
1	A	110	TYR
1	A	116	ILE
1	A	117	ILE
1	B	35	ILE
1	B	55	CYS
1	B	75	SER
1	B	80	LYS
1	B	85	ILE
1	B	87	GLU

Continued on next page...

PRELIMINARY VALIDATION REPORT

Continued from previous page...

Mol	Chain	Res	Type
1	B	88	GLU
1	B	103	LEU
1	B	110	TYR
1	B	116	ILE
1	B	118	ASP
1	B	122	LEU
1	C	26	SER
1	C	35	ILE
1	C	50	ARG
1	C	58	ASN
1	C	103	LEU
2	D	4	THR
2	D	27	ILE
2	D	29	THR
2	D	49	LEU
2	D	88	GLU
2	D	103	LEU
2	D	159	ASN

Some sidechains can be flipped to improve hydrogen bonding and reduce clashes. There are no such sidechains identified.

5.3.3 RNA (i)

There are no RNA molecules in this entry.

5.4 Non-standard residues in protein, DNA, RNA chains (i)

There are no non-standard protein/DNA/RNA residues in this entry.

5.5 Carbohydrates (i)

There are no carbohydrates in this entry.

5.6 Ligand geometry (i)

179 ligands are modelled in this entry.

In the following table, the Counts columns list the number of bonds (or angles) for which Mogul statistics could be retrieved, the number of bonds (or angles) that are observed in the model and

PRELIMINARY VALIDATION REPORT

the number of bonds (or angles) that are defined in the Chemical Component Dictionary. The Link column lists molecule types, if any, to which the group is linked. The Z score for a bond length (or angle) is the number of standard deviations the observed value is removed from the expected value. A bond length (or angle) with $|Z| > 2$ is considered an outlier worth inspection. RMSZ is the root-mean-square of all Z scores of the bond lengths (or angles).

Mol	Type	Chain	Res	Link	Bond lengths			Bond angles		
					Counts	RMSZ	#|Z| > 2	Counts	RMSZ	#|Z| > 2
3	PG4	E	1	-	12,12,12	0.95	0	11,11,11	1.38	1 (9%)
3	PG4	E	2	-	12,12,12	0.55	0	11,11,11	0.46	0
3	PG4	E	3	-	12,12,12	1.34	2 (16%)	11,11,11	1.63	1 (9%)
3	PG4	E	4	-	12,12,12	0.46	0	11,11,11	0.29	0
3	PG4	E	5	-	12,12,12	0.58	0	11,11,11	1.24	3 (27%)
9	EDO	F	1	-	3,3,3	1.14	0	2,2,2	1.41	0
9	EDO	F	10	-	3,3,3	0.45	0	2,2,2	0.36	0
9	EDO	F	11	-	3,3,3	0.46	0	2,2,2	0.37	0
9	EDO	F	12	-	3,3,3	0.57	0	2,2,2	0.46	0
9	EDO	F	13	-	3,3,3	0.45	0	2,2,2	0.36	0
9	EDO	F	14	-	3,3,3	0.53	0	2,2,2	1.21	0
9	EDO	F	15	-	3,3,3	0.45	0	2,2,2	0.36	0
9	EDO	F	16	-	3,3,3	0.46	0	2,2,2	0.36	0
9	EDO	F	17	-	3,3,3	0.45	0	2,2,2	0.37	0
9	EDO	F	18	-	3,3,3	0.35	0	2,2,2	0.23	0
9	EDO	F	19	-	3,3,3	0.48	0	2,2,2	0.20	0
9	EDO	F	2	-	3,3,3	0.48	0	2,2,2	0.37	0
9	EDO	F	20	-	3,3,3	0.88	0	2,2,2	0.74	0
9	EDO	F	21	-	3,3,3	0.58	0	2,2,2	0.14	0
9	EDO	F	22	-	3,3,3	0.70	0	2,2,2	1.05	0
9	EDO	F	23	-	3,3,3	0.82	0	2,2,2	0.42	0
9	EDO	F	24	-	3,3,3	1.19	0	2,2,2	0.55	0
9	EDO	F	25	-	3,3,3	0.98	0	2,2,2	1.19	0
9	EDO	F	26	-	3,3,3	0.45	0	2,2,2	0.37	0
9	EDO	F	27	-	3,3,3	0.38	0	2,2,2	0.85	0
9	EDO	F	28	-	3,3,3	0.30	0	2,2,2	0.66	0
9	EDO	F	29	-	3,3,3	0.45	0	2,2,2	0.36	0
9	EDO	F	3	-	3,3,3	0.43	0	2,2,2	0.51	0
9	EDO	F	30	-	3,3,3	0.79	0	2,2,2	0.80	0
9	EDO	F	31	-	3,3,3	0.45	0	2,2,2	0.37	0
9	EDO	F	32	-	3,3,3	0.45	0	2,2,2	0.37	0
9	EDO	F	33	-	3,3,3	1.41	0	2,2,2	0.41	0
9	EDO	F	4	-	3,3,3	0.44	0	2,2,2	0.49	0
9	EDO	F	5	-	3,3,3	0.45	0	2,2,2	0.36	0
9	EDO	F	6	-	3,3,3	0.46	0	2,2,2	0.37	0
9	EDO	F	7	-	3,3,3	0.45	0	2,2,2	0.36	0
9	EDO	F	8	-	3,3,3	0.45	0	2,2,2	0.36	0

PRELIMINARY VALIDATION REPORT

Mol	Type	Chain	Res	Link	Bond lengths			Bond angles		
					Counts	RMSZ	#|Z| > 2	Counts	RMSZ	#|Z| > 2
9	EDO	F	9	-	3,3,3	0.45	0	2,2,2	0.37	0
10	GOL	G	1	-	5,5,5	0.97	0	5,5,5	0.93	0
10	GOL	G	2	-	5,5,5	1.55	1 (20%)	5,5,5	0.75	0
10	GOL	G	3	-	5,5,5	1.35	1 (20%)	5,5,5	1.26	1 (20%)
10	GOL	G	4	-	5,5,5	1.74	2 (40%)	5,5,5	2.21	2 (40%)
10	GOL	G	5	-	5,5,5	1.47	1 (20%)	5,5,5	2.49	3 (60%)
10	GOL	G	6	-	5,5,5	1.20	0	5,5,5	2.11	3 (60%)
11	PDO	H	1	-	4,4,4	0.55	0	3,3,3	2.02	1 (33%)
11	PDO	H	10	-	4,4,4	0.32	0	3,3,3	0.36	0
11	PDO	H	11	-	4,4,4	0.33	0	3,3,3	0.36	0
11	PDO	H	12	-	4,4,4	0.33	0	3,3,3	0.36	0
11	PDO	H	13	-	4,4,4	0.92	0	3,3,3	0.81	0
11	PDO	H	14	-	4,4,4	0.40	0	3,3,3	0.94	0
11	PDO	H	2	-	4,4,4	0.33	0	3,3,3	0.36	0
11	PDO	H	3	-	4,4,4	0.33	0	3,3,3	0.36	0
11	PDO	H	4	-	4,4,4	0.33	0	3,3,3	0.37	0
11	PDO	H	5	-	4,4,4	0.33	0	3,3,3	0.36	0
11	PDO	H	6	-	4,4,4	0.33	0	3,3,3	0.36	0
11	PDO	H	7	-	4,4,4	0.33	0	3,3,3	0.36	0
11	PDO	H	8	-	4,4,4	0.33	0	3,3,3	0.36	0
11	PDO	H	9	-	4,4,4	0.33	0	3,3,3	0.36	0
12	EOH	I	1	-	2,2,2	0.44	0	1,1,1	0.28	0
12	EOH	I	10	-	2,2,2	0.39	0	1,1,1	0.38	0
12	EOH	I	11	-	2,2,2	0.39	0	1,1,1	0.38	0
12	EOH	I	12	-	2,2,2	0.40	0	1,1,1	0.38	0
12	EOH	I	13	-	2,2,2	0.40	0	1,1,1	0.38	0
12	EOH	I	14	-	2,2,2	0.40	0	1,1,1	0.38	0
12	EOH	I	15	-	2,2,2	0.39	0	1,1,1	0.38	0
12	EOH	I	16	-	2,2,2	0.40	0	1,1,1	0.38	0
12	EOH	I	17	-	2,2,2	0.40	0	1,1,1	0.38	0
12	EOH	I	18	-	2,2,2	0.39	0	1,1,1	0.38	0
12	EOH	I	19	-	2,2,2	0.39	0	1,1,1	0.38	0
12	EOH	I	2	-	2,2,2	0.40	0	1,1,1	0.38	0
12	EOH	I	20	-	2,2,2	0.21	0	1,1,1	0.55	0
12	EOH	I	21	-	2,2,2	0.40	0	1,1,1	0.38	0
12	EOH	I	22	-	2,2,2	0.39	0	1,1,1	0.38	0
12	EOH	I	23	-	2,2,2	0.39	0	1,1,1	0.38	0
12	EOH	I	24	-	2,2,2	0.40	0	1,1,1	0.64	0
12	EOH	I	25	-	2,2,2	0.40	0	1,1,1	0.38	0
12	EOH	I	26	-	2,2,2	0.40	0	1,1,1	0.38	0
12	EOH	I	27	-	2,2,2	0.39	0	1,1,1	0.38	0
12	EOH	I	28	-	2,2,2	0.49	0	1,1,1	0.38	0

PRELIMINARY VALIDATION REPORT

Mol	Type	Chain	Res	Link	Bond lengths			Bond angles		
					Counts	RMSZ	#|Z| > 2	Counts	RMSZ	#|Z| > 2
12	EOH	I	29	-	2,2,2	0.39	0	1,1,1	0.38	0
12	EOH	I	3	-	2,2,2	0.40	0	1,1,1	0.38	0
12	EOH	I	30	-	2,2,2	0.52	0	1,1,1	0.27	0
12	EOH	I	31	-	2,2,2	0.39	0	1,1,1	0.38	0
12	EOH	I	32	-	2,2,2	0.40	0	1,1,1	0.38	0
12	EOH	I	33	-	2,2,2	0.39	0	1,1,1	0.38	0
12	EOH	I	34	-	2,2,2	0.39	0	1,1,1	0.38	0
12	EOH	I	35	-	2,2,2	0.62	0	1,1,1	0.83	0
12	EOH	I	36	-	2,2,2	0.47	0	1,1,1	0.42	0
12	EOH	I	37	-	2,2,2	0.59	0	1,1,1	0.43	0
12	EOH	I	38	-	2,2,2	0.24	0	1,1,1	0.39	0
12	EOH	I	39	-	2,2,2	0.37	0	1,1,1	0.18	0
12	EOH	I	4	-	2,2,2	0.40	0	1,1,1	0.39	0
12	EOH	I	40	-	2,2,2	0.39	0	1,1,1	0.38	0
12	EOH	I	41	-	2,2,2	0.40	0	1,1,1	0.38	0
12	EOH	I	42	-	2,2,2	0.39	0	1,1,1	0.38	0
12	EOH	I	43	-	2,2,2	0.54	0	1,1,1	0.11	0
12	EOH	I	44	-	2,2,2	0.68	0	1,1,1	0.71	0
12	EOH	I	45	-	2,2,2	0.55	0	1,1,1	0.82	0
12	EOH	I	46	-	2,2,2	0.39	0	1,1,1	0.38	0
12	EOH	I	47	-	2,2,2	0.18	0	1,1,1	0.28	0
12	EOH	I	5	-	2,2,2	0.03	0	1,1,1	0.13	0
12	EOH	I	6	-	2,2,2	0.39	0	1,1,1	0.38	0
12	EOH	I	7	-	2,2,2	0.39	0	1,1,1	0.38	0
12	EOH	I	8	-	2,2,2	0.39	0	1,1,1	0.38	0
12	EOH	I	9	-	2,2,2	0.39	0	1,1,1	0.38	0
13	MOH	J	1	-	1,1,1	0.19	0	0,0,0	0.00	-
13	MOH	J	10	-	1,1,1	0.10	0	0,0,0	0.00	-
13	MOH	J	11	-	1,1,1	0.08	0	0,0,0	0.00	-
13	MOH	J	12	-	1,1,1	0.07	0	0,0,0	0.00	-
13	MOH	J	13	-	1,1,1	0.05	0	0,0,0	0.00	-
13	MOH	J	14	-	1,1,1	0.01	0	0,0,0	0.00	-
13	MOH	J	15	-	1,1,1	0.07	0	0,0,0	0.00	-
13	MOH	J	16	-	1,1,1	0.07	0	0,0,0	0.00	-
13	MOH	J	17	-	1,1,1	0.07	0	0,0,0	0.00	-
13	MOH	J	18	-	1,1,1	0.11	0	0,0,0	0.00	-
13	MOH	J	19	-	1,1,1	0.08	0	0,0,0	0.00	-
13	MOH	J	2	-	1,1,1	0.08	0	0,0,0	0.00	-
13	MOH	J	20	-	1,1,1	0.11	0	0,0,0	0.00	-
13	MOH	J	21	-	1,1,1	0.07	0	0,0,0	0.00	-
13	MOH	J	22	-	1,1,1	0.08	0	0,0,0	0.00	-
13	MOH	J	23	-	1,1,1	0.07	0	0,0,0	0.00	-
13	MOH	J	24	-	1,1,1	0.30	0	0,0,0	0.00	-

PRELIMINARY VALIDATION REPORT

Mol	Type	Chain	Res	Link	Bond lengths			Bond angles		
					Counts	RMSZ	#|Z| > 2	Counts	RMSZ	#|Z| > 2
13	MOH	J	25	-	1,1,1	0.07	0	0,0,0	0.00	-
13	MOH	J	3	-	1,1,1	0.07	0	0,0,0	0.00	-
13	MOH	J	4	-	1,1,1	0.08	0	0,0,0	0.00	-
13	MOH	J	5	-	1,1,1	0.06	0	0,0,0	0.00	-
13	MOH	J	6	-	1,1,1	0.07	0	0,0,0	0.00	-
13	MOH	J	7	-	1,1,1	0.08	0	0,0,0	0.00	-
13	MOH	J	8	-	1,1,1	0.08	0	0,0,0	0.00	-
13	MOH	J	9	-	1,1,1	0.06	0	0,0,0	0.00	-
14	PGO	K	1	-	4,4,4	2.61	1 (25%)	2,4,4	1.33	0
14	PGO	K	10	-	4,4,4	1.34	1 (25%)	2,4,4	1.45	0
14	PGO	K	2	-	4,4,4	0.46	0	2,4,4	0.42	0
14	PGO	K	3	-	4,4,4	1.47	0	2,4,4	1.05	0
14	PGO	K	4	-	4,4,4	1.06	0	2,4,4	2.30	2 (100%)
14	PGO	K	5	-	4,4,4	0.88	0	2,4,4	3.57	1 (50%)
14	PGO	K	6	-	4,4,4	0.66	0	2,4,4	3.18	1 (50%)
14	PGO	K	7	-	4,4,4	0.35	0	2,4,4	0.77	0
14	PGO	K	8	-	4,4,4	0.46	0	2,4,4	6.04	1 (50%)
14	PGO	K	9	-	4,4,4	0.46	0	2,4,4	0.44	0
15	PGE	L	1	-	9,9,9	0.45	0	8,8,8	0.30	0
15	PGE	L	2	-	9,9,9	0.45	0	8,8,8	0.30	0
15	PGE	L	3	-	9,9,9	0.45	0	8,8,8	0.30	0
15	PGE	L	4	-	9,9,9	0.45	0	8,8,8	0.30	0
15	PGE	L	5	-	9,9,9	1.26	2 (22%)	8,8,8	2.09	2 (25%)
5	PEG	M	1	-	6,6,6	0.44	0	5,5,5	0.32	0
5	PEG	M	10	-	6,6,6	0.47	0	5,5,5	0.91	0
5	PEG	M	11	-	6,6,6	0.31	0	5,5,5	1.29	1 (20%)
5	PEG	M	2	-	6,6,6	0.44	0	5,5,5	0.32	0
5	PEG	M	3	-	6,6,6	0.44	0	5,5,5	0.32	0
5	PEG	M	4	-	6,6,6	0.44	0	5,5,5	0.32	0
5	PEG	M	5	-	6,6,6	0.43	0	5,5,5	0.32	0
5	PEG	M	6	-	6,6,6	0.54	0	5,5,5	1.95	2 (40%)
5	PEG	M	7	-	6,6,6	0.65	0	5,5,5	2.39	3 (60%)
5	PEG	M	8	-	6,6,6	0.59	0	5,5,5	1.21	0
5	PEG	M	9	-	6,6,6	0.61	0	5,5,5	1.13	1 (20%)
16	ETX	N	1	-	5,5,5	0.42	0	4,4,4	0.53	0
16	ETX	N	2	-	5,5,5	0.65	0	4,4,4	0.80	0
16	ETX	N	3	-	5,5,5	0.44	0	4,4,4	0.30	0
16	ETX	N	4	-	5,5,5	0.44	0	4,4,4	0.29	0
16	ETX	N	5	-	5,5,5	0.80	0	4,4,4	1.63	1 (25%)
16	ETX	N	6	-	5,5,5	0.33	0	4,4,4	0.76	0

PRELIMINARY VALIDATION REPORT

Mol	Type	Chain	Res	Link	Bond lengths			Bond angles		
					Counts	RMSZ	#|Z| > 2	Counts	RMSZ	#|Z| > 2
16	ETX	N	7	-	5,5,5	0.45	0	4,4,4	0.30	0
16	ETX	N	8	-	5,5,5	0.54	0	4,4,4	1.20	1 (25%)
4	PG0	P	1	-	7,7,7	0.45	0	6,6,6	0.27	0
4	PG0	P	2	-	7,7,7	0.55	0	6,6,6	1.53	1 (16%)
4	PG0	P	3	-	7,7,7	0.72	0	6,6,6	0.83	0
4	PG0	P	4	-	7,7,7	0.45	0	6,6,6	0.27	0
4	PG0	P	5	-	7,7,7	0.77	0	6,6,6	0.67	0
4	PG0	P	6	-	7,7,7	0.45	0	6,6,6	0.27	0
4	PG0	P	7	-	7,7,7	0.45	0	6,6,6	0.27	0
4	PG0	P	8	-	7,7,7	0.32	0	6,6,6	1.43	2 (33%)
4	PG0	P	9	-	7,7,7	1.08	1 (14%)	6,6,6	1.87	3 (50%)
6	P6G	Q	1	-	18,18,18	0.46	0	17,17,17	0.28	0
6	P6G	Q	2	-	18,18,18	0.55	0	17,17,17	0.78	1 (5%)
6	P6G	Q	3	-	18,18,18	0.82	0	17,17,17	1.62	4 (23%)
7	P33	R	1	-	21,21,21	0.48	0	20,20,20	0.31	0
8	ETE	S	1	-	13,13,13	0.70	0	12,12,12	1.46	2 (16%)
8	ETE	S	2	-	13,13,13	0.65	0	12,12,12	1.34	3 (25%)

In the following table, the Chirals column lists the number of chiral outliers, the number of chiral centers analysed, the number of these observed in the model and the number defined in the Chemical Component Dictionary. Similar counts are reported in the Torsion and Rings columns. '-' means no outliers of that kind were identified.

Mol	Type	Chain	Res	Link	Chirals	Torsions	Rings
3	PG4	E	1	-	-	0/10/10/10	0/0/0/0
3	PG4	E	2	-	-	0/10/10/10	0/0/0/0
3	PG4	E	3	-	-	0/10/10/10	0/0/0/0
3	PG4	E	4	-	-	0/10/10/10	0/0/0/0
3	PG4	E	5	-	-	0/10/10/10	0/0/0/0
9	EDO	F	1	-	-	0/1/1/1	0/0/0/0
9	EDO	F	10	-	-	0/1/1/1	0/0/0/0
9	EDO	F	11	-	-	0/1/1/1	0/0/0/0
9	EDO	F	12	-	-	0/1/1/1	0/0/0/0
9	EDO	F	13	-	-	0/1/1/1	0/0/0/0
9	EDO	F	14	-	-	0/1/1/1	0/0/0/0
9	EDO	F	15	-	-	0/1/1/1	0/0/0/0
9	EDO	F	16	-	-	0/1/1/1	0/0/0/0
9	EDO	F	17	-	-	0/1/1/1	0/0/0/0
9	EDO	F	18	-	-	0/1/1/1	0/0/0/0
9	EDO	F	19	-	-	0/1/1/1	0/0/0/0
9	EDO	F	2	-	-	0/1/1/1	0/0/0/0

Continued on next page...

PRELIMINARY VALIDATION REPORT

Continued from previous page...

Mol	Type	Chain	Res	Link	Chirals	Torsions	Rings
9	EDO	F	20	-	-	0/1/1/1	0/0/0/0
9	EDO	F	21	-	-	0/1/1/1	0/0/0/0
9	EDO	F	22	-	-	0/1/1/1	0/0/0/0
9	EDO	F	23	-	-	0/1/1/1	0/0/0/0
9	EDO	F	24	-	-	0/1/1/1	0/0/0/0
9	EDO	F	25	-	-	0/1/1/1	0/0/0/0
9	EDO	F	26	-	-	0/1/1/1	0/0/0/0
9	EDO	F	27	-	-	0/1/1/1	0/0/0/0
9	EDO	F	28	-	-	0/1/1/1	0/0/0/0
9	EDO	F	29	-	-	0/1/1/1	0/0/0/0
9	EDO	F	3	-	-	0/1/1/1	0/0/0/0
9	EDO	F	30	-	-	0/1/1/1	0/0/0/0
9	EDO	F	31	-	-	0/1/1/1	0/0/0/0
9	EDO	F	32	-	-	0/1/1/1	0/0/0/0
9	EDO	F	33	-	-	0/1/1/1	0/0/0/0
9	EDO	F	4	-	-	0/1/1/1	0/0/0/0
9	EDO	F	5	-	-	0/1/1/1	0/0/0/0
9	EDO	F	6	-	-	0/1/1/1	0/0/0/0
9	EDO	F	7	-	-	0/1/1/1	0/0/0/0
9	EDO	F	8	-	-	0/1/1/1	0/0/0/0
9	EDO	F	9	-	-	0/1/1/1	0/0/0/0
10	GOL	G	1	-	-	0/4/4/4	0/0/0/0
10	GOL	G	2	-	-	0/4/4/4	0/0/0/0
10	GOL	G	3	-	-	0/4/4/4	0/0/0/0
10	GOL	G	4	-	-	0/4/4/4	0/0/0/0
10	GOL	G	5	-	-	0/4/4/4	0/0/0/0
10	GOL	G	6	-	-	0/4/4/4	0/0/0/0
11	PDO	H	1	-	-	0/2/2/2	0/0/0/0
11	PDO	H	10	-	-	0/2/2/2	0/0/0/0
11	PDO	H	11	-	-	0/2/2/2	0/0/0/0
11	PDO	H	12	-	-	0/2/2/2	0/0/0/0
11	PDO	H	13	-	-	0/2/2/2	0/0/0/0
11	PDO	H	14	-	-	0/2/2/2	0/0/0/0
11	PDO	H	2	-	-	0/2/2/2	0/0/0/0
11	PDO	H	3	-	-	0/2/2/2	0/0/0/0
11	PDO	H	4	-	-	0/2/2/2	0/0/0/0
11	PDO	H	5	-	-	0/2/2/2	0/0/0/0
11	PDO	H	6	-	-	0/2/2/2	0/0/0/0
11	PDO	H	7	-	-	0/2/2/2	0/0/0/0
11	PDO	H	8	-	-	0/2/2/2	0/0/0/0
11	PDO	H	9	-	-	0/2/2/2	0/0/0/0
12	EOH	I	1	-	-	0/0/0/0	0/0/0/0

Continued on next page...

PRELIMINARY VALIDATION REPORT

Continued from previous page...

Mol	Type	Chain	Res	Link	Chirals	Torsions	Rings
12	EOH	I	10	-	-	0/0/0/0	0/0/0/0
12	EOH	I	11	-	-	0/0/0/0	0/0/0/0
12	EOH	I	12	-	-	0/0/0/0	0/0/0/0
12	EOH	I	13	-	-	0/0/0/0	0/0/0/0
12	EOH	I	14	-	-	0/0/0/0	0/0/0/0
12	EOH	I	15	-	-	0/0/0/0	0/0/0/0
12	EOH	I	16	-	-	0/0/0/0	0/0/0/0
12	EOH	I	17	-	-	0/0/0/0	0/0/0/0
12	EOH	I	18	-	-	0/0/0/0	0/0/0/0
12	EOH	I	19	-	-	0/0/0/0	0/0/0/0
12	EOH	I	2	-	-	0/0/0/0	0/0/0/0
12	EOH	I	20	-	-	0/0/0/0	0/0/0/0
12	EOH	I	21	-	-	0/0/0/0	0/0/0/0
12	EOH	I	22	-	-	0/0/0/0	0/0/0/0
12	EOH	I	23	-	-	0/0/0/0	0/0/0/0
12	EOH	I	24	-	-	0/0/0/0	0/0/0/0
12	EOH	I	25	-	-	0/0/0/0	0/0/0/0
12	EOH	I	26	-	-	0/0/0/0	0/0/0/0
12	EOH	I	27	-	-	0/0/0/0	0/0/0/0
12	EOH	I	28	-	-	0/0/0/0	0/0/0/0
12	EOH	I	29	-	-	0/0/0/0	0/0/0/0
12	EOH	I	3	-	-	0/0/0/0	0/0/0/0
12	EOH	I	30	-	-	0/0/0/0	0/0/0/0
12	EOH	I	31	-	-	0/0/0/0	0/0/0/0
12	EOH	I	32	-	-	0/0/0/0	0/0/0/0
12	EOH	I	33	-	-	0/0/0/0	0/0/0/0
12	EOH	I	34	-	-	0/0/0/0	0/0/0/0
12	EOH	I	35	-	-	0/0/0/0	0/0/0/0
12	EOH	I	36	-	-	0/0/0/0	0/0/0/0
12	EOH	I	37	-	-	0/0/0/0	0/0/0/0
12	EOH	I	38	-	-	0/0/0/0	0/0/0/0
12	EOH	I	39	-	-	0/0/0/0	0/0/0/0
12	EOH	I	4	-	-	0/0/0/0	0/0/0/0
12	EOH	I	40	-	-	0/0/0/0	0/0/0/0
12	EOH	I	41	-	-	0/0/0/0	0/0/0/0
12	EOH	I	42	-	-	0/0/0/0	0/0/0/0
12	EOH	I	43	-	-	0/0/0/0	0/0/0/0
12	EOH	I	44	-	-	0/0/0/0	0/0/0/0
12	EOH	I	45	-	-	0/0/0/0	0/0/0/0
12	EOH	I	46	-	-	0/0/0/0	0/0/0/0
12	EOH	I	47	-	-	0/0/0/0	0/0/0/0
12	EOH	I	5	-	-	0/0/0/0	0/0/0/0

Continued on next page...

PRELIMINARY VALIDATION REPORT

Continued from previous page...

Mol	Type	Chain	Res	Link	Chirals	Torsions	Rings
12	EOH	I	6	-	-	0/0/0/0	0/0/0/0
12	EOH	I	7	-	-	0/0/0/0	0/0/0/0
12	EOH	I	8	-	-	0/0/0/0	0/0/0/0
12	EOH	I	9	-	-	0/0/0/0	0/0/0/0
13	MOH	J	1	-	-	0/0/0/0	0/0/0/0
13	MOH	J	10	-	-	0/0/0/0	0/0/0/0
13	MOH	J	11	-	-	0/0/0/0	0/0/0/0
13	MOH	J	12	-	-	0/0/0/0	0/0/0/0
13	MOH	J	13	-	-	0/0/0/0	0/0/0/0
13	MOH	J	14	-	-	0/0/0/0	0/0/0/0
13	MOH	J	15	-	-	0/0/0/0	0/0/0/0
13	MOH	J	16	-	-	0/0/0/0	0/0/0/0
13	MOH	J	17	-	-	0/0/0/0	0/0/0/0
13	MOH	J	18	-	-	0/0/0/0	0/0/0/0
13	MOH	J	19	-	-	0/0/0/0	0/0/0/0
13	MOH	J	2	-	-	0/0/0/0	0/0/0/0
13	MOH	J	20	-	-	0/0/0/0	0/0/0/0
13	MOH	J	21	-	-	0/0/0/0	0/0/0/0
13	MOH	J	22	-	-	0/0/0/0	0/0/0/0
13	MOH	J	23	-	-	0/0/0/0	0/0/0/0
13	MOH	J	24	-	-	0/0/0/0	0/0/0/0
13	MOH	J	25	-	-	0/0/0/0	0/0/0/0
13	MOH	J	3	-	-	0/0/0/0	0/0/0/0
13	MOH	J	4	-	-	0/0/0/0	0/0/0/0
13	MOH	J	5	-	-	0/0/0/0	0/0/0/0
13	MOH	J	6	-	-	0/0/0/0	0/0/0/0
13	MOH	J	7	-	-	0/0/0/0	0/0/0/0
13	MOH	J	8	-	-	0/0/0/0	0/0/0/0
13	MOH	J	9	-	-	0/0/0/0	0/0/0/0
14	PGO	K	1	-	1/1/1/1	0/2/2/2	0/0/0/0
14	PGO	K	10	-	-	0/2/2/2	0/0/0/0
14	PGO	K	2	-	-	0/2/2/2	0/0/0/0
14	PGO	K	3	-	-	0/2/2/2	0/0/0/0
14	PGO	K	4	-	-	0/2/2/2	0/0/0/0
14	PGO	K	5	-	-	0/2/2/2	0/0/0/0
14	PGO	K	6	-	-	0/2/2/2	0/0/0/0
14	PGO	K	7	-	-	0/2/2/2	0/0/0/0
14	PGO	K	8	-	-	0/2/2/2	0/0/0/0
14	PGO	K	9	-	-	0/2/2/2	0/0/0/0
15	PGE	L	1	-	-	0/7/7/7	0/0/0/0
15	PGE	L	2	-	-	0/7/7/7	0/0/0/0
15	PGE	L	3	-	-	0/7/7/7	0/0/0/0

Continued on next page...

PRELIMINARY VALIDATION REPORT

Continued from previous page...

Mol	Type	Chain	Res	Link	Chirals	Torsions	Rings
15	PGE	L	4	-	-	0/7/7/7	0/0/0/0
15	PGE	L	5	-	-	0/7/7/7	0/0/0/0
5	PEG	M	1	-	-	0/4/4/4	0/0/0/0
5	PEG	M	10	-	-	0/4/4/4	0/0/0/0
5	PEG	M	11	-	-	0/4/4/4	0/0/0/0
5	PEG	M	2	-	-	0/4/4/4	0/0/0/0
5	PEG	M	3	-	-	0/4/4/4	0/0/0/0
5	PEG	M	4	-	-	0/4/4/4	0/0/0/0
5	PEG	M	5	-	-	0/4/4/4	0/0/0/0
5	PEG	M	6	-	-	0/4/4/4	0/0/0/0
5	PEG	M	7	-	-	0/4/4/4	0/0/0/0
5	PEG	M	8	-	-	0/4/4/4	0/0/0/0
5	PEG	M	9	-	-	0/4/4/4	0/0/0/0
16	ETX	N	1	-	-	0/3/3/3	0/0/0/0
16	ETX	N	2	-	-	0/3/3/3	0/0/0/0
16	ETX	N	3	-	-	0/3/3/3	0/0/0/0
16	ETX	N	4	-	-	0/3/3/3	0/0/0/0
16	ETX	N	5	-	-	0/3/3/3	0/0/0/0
16	ETX	N	6	-	-	0/3/3/3	0/0/0/0
16	ETX	N	7	-	-	0/3/3/3	0/0/0/0
16	ETX	N	8	-	-	0/3/3/3	0/0/0/0
4	PG0	P	1	-	-	0/5/5/5	0/0/0/0
4	PG0	P	2	-	-	0/5/5/5	0/0/0/0
4	PG0	P	3	-	-	0/5/5/5	0/0/0/0
4	PG0	P	4	-	-	0/5/5/5	0/0/0/0
4	PG0	P	5	-	-	0/5/5/5	0/0/0/0
4	PG0	P	6	-	-	0/5/5/5	0/0/0/0
4	PG0	P	7	-	-	0/5/5/5	0/0/0/0
4	PG0	P	8	-	-	0/5/5/5	0/0/0/0
4	PG0	P	9	-	-	0/5/5/5	0/0/0/0
6	P6G	Q	1	-	-	0/16/16/16	0/0/0/0
6	P6G	Q	2	-	-	0/16/16/16	0/0/0/0
6	P6G	Q	3	-	-	0/16/16/16	0/0/0/0
7	P33	R	1	-	-	0/19/19/19	0/0/0/0
8	ETE	S	1	-	-	0/11/11/11	0/0/0/0
8	ETE	S	2	-	-	0/11/11/11	0/0/0/0

All (12) bond length outliers are listed below:

Mol	Chain	Res	Type	Atoms	Z	Observed(Å)	Ideal(Å)
14	K	1	PGO	O2-C2	-5.10	1.19	1.43
10	G	4	GOL	O2-C2	-3.12	1.34	1.43

Continued on next page...

PRELIMINARY VALIDATION REPORT

Continued from previous page...

Mol	Chain	Res	Type	Atoms	Z	Observed(Å)	Ideal(Å)
10	G	2	GOL	O2-C2	-2.89	1.34	1.43
10	G	5	GOL	O2-C2	-2.87	1.35	1.43
3	E	3	PG4	O4-C6	-2.37	1.31	1.42
4	P	9	PG0	O1-C3	-2.36	1.31	1.42
10	G	3	GOL	O2-C2	-2.35	1.36	1.43
14	K	10	PGO	O1-C1	-2.33	1.32	1.42
3	E	3	PG4	O3-C5	-2.31	1.32	1.42
15	L	5	PGE	O1-C1	-2.26	1.30	1.42
15	L	5	PGE	O2-C2	-2.20	1.32	1.42
10	G	4	GOL	C3-C2	-2.17	1.44	1.52

All (47) bond angle outliers are listed below:

Mol	Chain	Res	Type	Atoms	Z	Observed(°)	Ideal(°)
15	L	5	PGE	C3-O2-C2	-4.34	94.52	113.30
10	G	4	GOL	O2-C2-C3	-3.70	91.34	108.84
3	E	3	PG4	C5-O3-C4	-3.35	98.79	113.30
11	H	1	PDO	O3-C3-C2	-3.23	90.31	111.65
5	M	7	PEG	O2-C3-C4	-2.85	96.98	110.15
10	G	4	GOL	O3-C3-C2	-2.74	96.26	110.07
10	G	5	GOL	O2-C2-C3	-2.72	96.01	108.84
16	N	5	ETX	O1-C1-C2	-2.68	96.48	111.89
5	M	7	PEG	O1-C1-C2	-2.64	96.71	111.89
6	Q	3	P6G	C14-O13-C12	-2.35	103.14	113.30
4	P	9	PG0	O1-C3-C4	-2.33	99.70	110.41
14	K	4	PGO	O2-C2-C3	-2.32	98.81	109.46
10	G	6	GOL	C3-C2-C1	-2.19	102.83	111.52
5	M	11	PEG	O2-C3-C4	-2.16	100.16	110.15
6	Q	2	P6G	O16-C17-C18	-2.06	100.64	110.15
8	S	2	ETE	OH4-C13-C23	-2.04	101.03	110.41
3	E	5	PG4	C3-O2-C2	-2.03	104.49	113.30
4	P	8	PG0	O1-C3-C4	2.03	119.73	110.41
8	S	2	ETE	OH3-C22-C12	2.05	119.63	110.15
10	G	3	GOL	O3-C3-C2	2.10	120.64	110.07
6	Q	3	P6G	O13-C14-C15	2.20	120.49	110.41
3	E	5	PG4	C7-O4-C6	2.20	122.83	113.30
3	E	5	PG4	O4-C6-C5	2.22	120.61	110.41
5	M	9	PEG	C3-O2-C2	2.26	123.07	113.30
14	K	4	PGO	O1-C1-C2	2.28	116.00	110.83
16	N	8	ETX	C3-O2-C2	2.33	121.31	112.88
8	S	2	ETE	OH5-C14-C24	2.44	121.62	110.41
5	M	6	PEG	O2-C3-C4	2.45	121.48	110.15

Continued on next page...

PRELIMINARY VALIDATION REPORT

Continued from previous page...

Mol	Chain	Res	Type	Atoms	Z	Observed(°)	Ideal(°)
10	G	5	GOL	O2-C2-C1	2.46	120.46	108.84
4	P	8	PG0	OTT-C1-C2	2.60	126.84	111.89
6	Q	3	P6G	O7-C8-C9	2.65	122.59	110.41
8	S	1	ETE	OH5-C14-C24	2.72	122.89	110.41
4	P	9	PG0	O1-C2-C1	2.72	122.71	110.15
4	P	9	PG0	C2-O1-C3	2.75	125.19	113.30
10	G	6	GOL	O3-C3-C2	2.76	123.95	110.07
10	G	6	GOL	O2-C2-C3	2.83	122.20	108.84
15	L	5	PGE	O2-C2-C1	2.86	123.33	110.15
8	S	1	ETE	C23-OH3-C22	2.92	125.94	113.30
3	E	1	PG4	O4-C7-C8	3.06	124.27	110.15
5	M	7	PEG	C3-O2-C2	3.08	126.66	113.30
4	P	2	PG0	O1-C2-C1	3.23	125.04	110.15
10	G	5	GOL	O1-C1-C2	3.38	127.11	110.07
5	M	6	PEG	O2-C2-C1	3.58	126.65	110.15
6	Q	3	P6G	O7-C6-C5	3.87	128.18	110.41
14	K	6	PGO	O1-C1-C2	4.48	120.96	110.83
14	K	5	PGO	O1-C1-C2	4.87	121.84	110.83
14	K	8	PGO	O1-C1-C2	8.54	130.16	110.83

All (1) chirality outliers are listed below:

Mol	Chain	Res	Type	Atom
14	K	1	PGO	C2

There are no torsion outliers.

There are no ring outliers.

84 monomers are involved in 212 short contacts:

Mol	Chain	Res	Type	Clashes	Symm-Clashes
3	E	1	PG4	2	0
3	E	3	PG4	4	0
3	E	4	PG4	9	0
3	E	5	PG4	1	0
9	F	1	EDO	1	0
9	F	10	EDO	1	0
9	F	13	EDO	1	0
9	F	14	EDO	3	0
9	F	19	EDO	1	0
9	F	25	EDO	1	0
9	F	27	EDO	1	0

Continued on next page...

PRELIMINARY VALIDATION REPORT

Continued from previous page...

Mol	Chain	Res	Type	Clashes	Symm-Clashes
9	F	28	EDO	1	0
9	F	3	EDO	2	0
9	F	30	EDO	2	0
9	F	33	EDO	1	0
9	F	5	EDO	2	0
9	F	6	EDO	1	0
9	F	7	EDO	1	0
9	F	9	EDO	2	0
10	G	2	GOL	2	0
10	G	3	GOL	1	0
10	G	6	GOL	1	0
11	H	1	PDO	3	0
11	H	10	PDO	11	0
11	H	2	PDO	1	0
11	H	3	PDO	2	0
11	H	6	PDO	7	0
11	H	7	PDO	1	0
11	H	9	PDO	1	0
12	I	1	EOH	1	0
12	I	10	EOH	1	0
12	I	13	EOH	11	0
12	I	15	EOH	2	0
12	I	16	EOH	4	0
12	I	19	EOH	2	0
12	I	25	EOH	1	0
12	I	26	EOH	1	0
12	I	28	EOH	3	0
12	I	29	EOH	1	0
12	I	33	EOH	2	0
12	I	35	EOH	1	0
12	I	39	EOH	1	0
12	I	40	EOH	1	0
12	I	43	EOH	2	0
12	I	5	EOH	2	0
13	J	10	MOH	1	0
13	J	21	MOH	2	0
13	J	6	MOH	1	0
14	K	1	PGO	2	0
14	K	4	PGO	4	0
14	K	5	PGO	10	0
14	K	8	PGO	1	0
14	K	9	PGO	5	0

Continued on next page...

PRELIMINARY VALIDATION REPORT

Continued from previous page...

Mol	Chain	Res	Type	Clashes	Symm-Clashes
15	L	1	PGE	1	0
15	L	2	PGE	5	0
15	L	3	PGE	1	0
15	L	4	PGE	3	0
15	L	5	PGE	2	0
5	M	1	PEG	8	0
5	M	2	PEG	6	0
5	M	4	PEG	6	0
5	M	5	PEG	1	0
5	M	6	PEG	4	0
5	M	7	PEG	4	0
16	N	1	ETX	12	0
16	N	2	ETX	6	0
16	N	4	ETX	8	0
16	N	6	ETX	2	0
16	N	8	ETX	1	0
4	P	1	PG0	2	0
4	P	2	PG0	3	0
4	P	3	PG0	5	0
4	P	4	PG0	4	0
4	P	5	PG0	3	0
4	P	6	PG0	7	0
4	P	7	PG0	5	0
4	P	8	PG0	1	0
4	P	9	PG0	5	0
6	Q	1	P6G	2	0
6	Q	2	P6G	15	0
6	Q	3	P6G	5	0
7	R	1	P33	14	0
8	S	1	ETE	4	0
8	S	2	ETE	1	0

5.7 Other polymers (i)

There are no such residues in this entry.

5.8 Polymer linkage issues (i)

The following chains have linkage breaks:

PRELIMINARY VALIDATION REPORT

Mol	Chain	Number of breaks
2	D	1

All chain breaks are listed below:

Model	Chain	Residue-1	Atom-1	Residue-2	Atom-2	Distance (Å)
1	D	29:THR	C	31:VAL	N	3.07

PRELIMINARY VALIDATION REPORT

6 Fit of model and data (i)

6.1 Protein, DNA and RNA chains (i)

In the following table, the column labelled '#RSRZ> 2' contains the number (and percentage) of RSRZ outliers, followed by percent RSRZ outliers for the chain as percentile scores relative to all X-ray entries and entries of similar resolution. The OWAB column contains the minimum, median, 95th percentile and maximum values of the occupancy-weighted average B-factor per residue. The column labelled 'Q< 0.9' lists the number of (and percentage) of residues with an average occupancy less than 0.9.

Mol	Chain	Analysed	<RSRZ>	#RSRZ>2			OWAB(Å^2)	Q<0.9
1	A	165/176 (93%)	0.58	17 (10%)	7	9	23, 38, 71, 84	0
1	B	161/176 (91%)	0.40	10 (6%)	21	27	22, 38, 62, 77	0
1	C	161/176 (91%)	0.38	9 (5%)	25	31	21, 33, 56, 83	0
2	D	166/166 (100%)	0.48	14 (8%)	12	15	21, 36, 63, 91	0
All	All	653/694 (94%)	0.46	50 (7%)	14	18	21, 37, 64, 91	0

All (50) RSRZ outliers are listed below:

Mol	Chain	Res	Type	RSRZ
1	C	29	THR	8.6
1	A	2	ALA	7.1
1	A	32	THR	6.3
1	A	28	PRO	5.1
1	A	29	THR	4.8
2	D	166	ILE	4.0
1	A	166	ILE	4.0
1	A	116	ILE	3.9
2	D	165	PRO	3.8
2	D	31	VAL	3.8
1	B	74	ILE	3.7
2	D	167	ILE	3.6
1	C	32	THR	3.5
2	D	4	THR	3.4
1	A	27	ILE	3.3
1	B	32	THR	3.3
1	A	31	VAL	3.2
1	C	27	ILE	3.1
2	D	162	TYR	3.0
2	D	1	MET	3.0
1	A	117	ILE	2.9

Continued on next page...

PRELIMINARY VALIDATION REPORT

Continued from previous page...

Mol	Chain	Res	Type	RSRZ
1	B	165	PRO	2.9
1	C	117	ILE	2.9
1	A	165	PRO	2.8
1	A	30	ASN	2.7
1	B	29	THR	2.7
1	B	4	THR	2.7
1	C	28	PRO	2.7
2	D	27	ILE	2.6
1	B	31	VAL	2.6
1	C	5	LYS	2.6
2	D	3	GLU	2.5
1	B	25	HIS	2.4
2	D	29	THR	2.4
2	D	130	PRO	2.4
1	A	19	LEU	2.3
1	C	24	GLN	2.3
1	A	85	ILE	2.3
1	B	115	ASN	2.2
1	B	34	ALA	2.2
1	A	25	HIS	2.1
1	C	30	ASN	2.1
1	A	55	CYS	2.1
1	B	55	CYS	2.1
1	C	164	PRO	2.1
1	A	162	TYR	2.1
2	D	2	ALA	2.1
1	A	143	GLY	2.1
2	D	163	VAL	2.1
2	D	23	ALA	2.0

6.2 Non-standard residues in protein, DNA, RNA chains (i)

There are no non-standard protein/DNA/RNA residues in this entry.

6.3 Carbohydrates (i)

There are no carbohydrates in this entry.

PRELIMINARY VALIDATION REPORT

6.4 Ligands ⓘ

In the following table, the Atoms column lists the number of modelled atoms in the group and the number defined in the chemical component dictionary. LLDF column lists the quality of electron density of the group with respect to its neighbouring residues in protein, DNA or RNA chains. The B-factors column lists the minimum, median, 95th percentile and maximum values of B factors of atoms in the group. The column labelled 'Q< 0.9' lists the number of atoms with occupancy less than 0.9.

Mol	Type	Chain	Res	Atoms	RSCC	RSR	LLDF	B-factors(Å^2)	Q<0.9
12	EOH	I	43	3/?	0.83	0.44	20.48	41,41,45,47	0
9	EDO	F	20	4/?	0.76	0.44	18.64	41,42,43,43	0
3	PG4	E	4	13/?	0.84	0.34	15.32	30,42,48,53	0
13	MOH	J	6	2/?	0.91	0.46	14.21	35,35,35,41	0
5	PEG	M	2	7/?	0.76	0.25	12.54	38,45,52,52	0
4	PG0	P	7	8/?	0.68	0.32	10.40	42,49,57,66	0
5	PEG	M	4	7/?	0.73	0.48	9.97	30,39,50,51	0
14	PGO	K	8	5/?	0.64	0.29	7.69	42,44,48,48	0
13	MOH	J	7	2/?	0.66	0.32	7.63	34,34,34,41	0
9	EDO	F	2	4/?	0.92	0.22	6.40	40,42,47,59	0
11	PDO	H	12	5/?	0.68	0.40	6.21	70,71,72,72	0
11	PDO	H	6	5/?	0.86	0.26	6.14	35,35,38,50	0
4	PG0	P	4	8/?	0.70	0.34	6.13	57,62,63,64	0
4	PG0	P	6	8/?	0.67	0.28	6.01	36,49,55,58	0
5	PEG	M	1	7/?	0.86	0.48	5.97	35,39,44,50	0
5	PEG	M	8	7/?	0.52	0.25	5.65	50,55,61,66	0
11	PDO	H	10	5/?	0.88	0.25	5.61	21,26,32,35	0
9	EDO	F	10	4/?	0.80	0.29	5.42	36,40,42,42	0
9	EDO	F	19	4/?	0.57	0.46	5.31	63,64,64,65	0
4	PG0	P	1	8/?	0.73	0.25	5.20	43,57,59,64	0
13	MOH	J	9	2/?	0.88	0.23	5.02	29,29,29,34	0
12	EOH	I	19	3/?	0.82	0.33	4.97	54,54,58,59	0
9	EDO	F	25	4/?	0.81	0.25	4.71	52,56,56,56	0
11	PDO	H	3	5/?	0.79	0.22	4.51	46,51,54,55	0
11	PDO	H	7	5/?	0.80	0.20	4.10	29,34,40,40	0
9	EDO	F	16	4/?	0.79	0.39	3.83	44,47,51,54	0
7	P33	R	1	22/?	0.74	0.38	3.82	44,59,74,77	0
13	MOH	J	2	2/?	0.84	0.21	3.66	40,40,40,42	0
15	PGE	L	4	10/?	0.78	0.36	3.54	56,63,69,84	0
12	EOH	I	11	3/?	0.84	0.19	2.97	32,32,32,44	0
5	PEG	M	5	7/?	0.79	0.19	2.77	30,32,36,37	0
12	EOH	I	15	3/?	0.91	0.23	2.76	30,30,36,42	0
12	EOH	I	31	3/?	0.79	0.29	2.42	49,49,52,55	0
12	EOH	I	45	3/?	0.74	0.26	2.19	46,46,46,51	0

Continued on next page...

PRELIMINARY VALIDATION REPORT

Continued from previous page...

Mol	Type	Chain	Res	Atoms	RSCC	RSR	LLDF	B-factors(Å^2)	Q<0.9
9	EDO	F	15	4/?	0.82	0.19	2.13	44,46,50,51	0
5	PEG	M	6	7/?	0.61	0.23	2.10	63,70,73,74	0
9	EDO	F	33	4/?	0.89	0.18	1.98	38,48,51,54	0
14	PGO	K	9	5/?	0.51	0.26	1.83	54,55,59,60	0
9	EDO	F	5	4/?	0.84	0.20	1.66	51,55,55,60	0
14	PGO	K	1	5/?	0.84	0.16	1.53	30,35,41,45	0
13	MOH	J	24	2/?	0.75	0.25	1.48	44,44,44,47	0
10	GOL	G	5	6/?	0.82	0.19	1.39	48,50,53,66	0
9	EDO	F	6	4/?	0.85	0.20	1.16	37,45,45,46	0
12	EOH	I	36	3/?	0.87	0.27	1.10	54,54,58,61	0
6	P6G	Q	1	19/?	0.84	0.16	0.97	27,47,51,61	0
4	PG0	P	2	8/?	0.78	0.20	0.92	47,51,58,59	0
9	EDO	F	7	4/?	0.83	0.17	0.89	43,46,46,52	0
9	EDO	F	13	4/?	0.75	0.26	0.87	55,58,60,62	0
6	P6G	Q	3	19/?	0.49	0.38	0.86	56,71,87,88	0
5	PEG	M	3	7/?	0.74	0.22	0.78	56,60,63,64	0
13	MOH	J	3	2/?	0.90	0.17	0.77	39,39,39,39	0
15	PGE	L	5	10/?	0.86	0.23	0.73	38,51,59,63	0
11	PDO	H	8	5/?	0.79	0.23	0.72	40,41,45,48	0
13	MOH	J	23	2/?	0.82	0.20	0.53	55,55,55,60	0
8	ETE	S	1	14/?	0.64	0.23	0.46	61,69,76,77	0
16	ETX	N	7	6/?	0.83	0.16	0.35	45,55,56,61	0
11	PDO	H	11	5/?	0.71	0.17	0.34	44,45,50,57	0
11	PDO	H	2	5/?	0.81	0.18	0.01	40,46,49,51	0
12	EOH	I	1	3/?	0.95	0.12	-0.18	33,33,35,42	0
12	EOH	I	44	3/?	0.76	0.24	-0.23	64,64,66,70	0
14	PGO	K	7	5/?	0.86	0.13	-0.35	44,50,60,61	0
12	EOH	I	3	3/?	0.89	0.16	-0.38	46,46,51,63	0
12	EOH	I	29	3/?	0.86	0.14	-0.63	53,53,54,61	0
12	EOH	I	6	3/?	0.84	0.15	-0.83	59,59,59,69	0
12	EOH	I	18	3/?	0.91	0.11	-1.14	39,39,46,56	0
13	MOH	J	10	2/?	0.55	0.28	-	62,62,62,62	0
8	ETE	S	2	14/?	0.59	0.27	-	56,69,75,80	0
6	P6G	Q	2	19/?	0.67	0.42	-	79,90,96,97	0
9	EDO	F	23	4/?	0.77	0.24	-	47,52,52,52	0
9	EDO	F	4	4/?	0.81	0.18	-	47,51,51,58	0
13	MOH	J	13	2/?	0.75	0.24	-	60,60,60,63	0
9	EDO	F	12	4/?	0.71	0.27	-	50,51,54,54	0
9	EDO	F	9	4/?	0.89	0.27	-	48,51,57,58	0
10	GOL	G	4	6/?	0.80	0.30	-	53,57,63,66	0
12	EOH	I	16	3/?	0.77	0.18	-	50,50,54,60	0
14	PGO	K	3	5/?	0.85	0.16	-	40,44,52,53	0

Continued on next page...

PRELIMINARY VALIDATION REPORT

Continued from previous page...

Mol	Type	Chain	Res	Atoms	RSCC	RSR	LLDF	B-factors(Å^2)	Q<0.9
12	EOH	I	10	3/?	0.67	0.20	-	57,57,59,63	0
4	PG0	P	8	8/?	0.58	0.30	-	55,61,65,70	0
9	EDO	F	3	4/?	0.91	0.16	-	41,43,43,44	0
13	MOH	J	4	2/?	0.82	0.25	-	39,39,39,44	0
13	MOH	J	20	2/?	0.56	0.46	-	65,65,65,67	0
12	EOH	I	42	3/?	0.90	0.21	-	31,31,38,42	0
12	EOH	I	5	3/?	0.80	0.19	-	49,49,52,55	0
9	EDO	F	32	4/?	0.73	0.24	-	70,74,74,79	0
11	PDO	H	9	5/?	0.81	0.20	-	55,56,60,66	0
5	PEG	M	7	7/?	0.72	0.19	-	55,59,62,67	0
13	MOH	J	25	2/?	0.85	0.26	-	55,55,55,68	0
14	PGO	K	2	5/?	0.79	0.17	-	51,60,64,65	0
12	EOH	I	20	3/?	0.71	0.23	-	59,59,68,71	0
16	ETX	N	3	6/?	0.43	0.60	-	52,63,63,70	0
13	MOH	J	11	2/?	0.87	0.23	-	63,63,63,65	0
11	PDO	H	1	5/?	0.72	0.33	-	68,70,76,77	0
12	EOH	I	17	3/?	0.83	0.18	-	52,52,55,56	0
11	PDO	H	5	5/?	0.82	0.17	-	53,55,59,60	0
14	PGO	K	6	5/?	0.74	0.23	-	49,50,57,58	0
13	MOH	J	19	2/?	0.83	0.19	-	47,47,47,54	0
4	PG0	P	3	8/?	0.68	0.28	-	49,59,62,68	0
3	PG4	E	3	13/?	0.83	0.28	-	41,47,52,56	0
15	PGE	L	3	10/?	0.68	0.39	-	56,71,77,83	0
16	ETX	N	4	6/?	0.67	0.36	-	63,74,75,78	0
15	PGE	L	2	10/?	0.53	0.26	-	68,71,76,76	0
12	EOH	I	13	3/?	0.30	0.23	-	57,57,60,62	0
12	EOH	I	4	3/?	0.85	0.27	-	41,41,48,51	0
12	EOH	I	38	3/?	0.62	0.27	-	50,50,54,57	0
9	EDO	F	1	4/?	0.83	0.30	-	52,57,57,61	0
10	GOL	G	3	6/?	0.83	0.23	-	42,47,51,59	0
12	EOH	I	30	3/?	0.86	0.31	-	45,45,48,56	0
11	PDO	H	13	5/?	0.66	0.30	-	48,54,56,61	0
12	EOH	I	26	3/?	0.87	0.12	-	59,59,63,63	0
12	EOH	I	35	3/?	0.83	0.17	-	55,55,67,69	0
13	MOH	J	17	2/?	0.73	0.22	-	45,45,45,45	0
12	EOH	I	23	3/?	0.83	0.23	-	48,48,51,51	0
9	EDO	F	26	4/?	0.76	0.26	-	73,75,77,88	0
14	PGO	K	5	5/?	0.75	0.19	-	62,65,69,77	0
12	EOH	I	33	3/?	0.82	0.26	-	46,46,49,57	0
12	EOH	I	25	3/?	0.83	0.19	-	65,65,72,80	0
12	EOH	I	24	3/?	0.64	0.19	-	67,67,71,73	0
11	PDO	H	14	5/?	0.76	0.21	-	50,54,65,75	0

Continued on next page...

PRELIMINARY VALIDATION REPORT

Continued from previous page...

Mol	Type	Chain	Res	Atoms	RSCC	RSR	LLDF	B-factors(Å^2)	Q<0.9
12	EOH	I	9	3/?	0.91	0.20	-	42,42,44,50	0
9	EDO	F	24	4/?	0.76	0.34	-	50,56,60,67	0
4	PG0	P	5	8/?	0.77	0.27	-	45,50,60,63	0
16	ETX	N	2	6/?	0.60	0.26	-	58,62,65,66	0
12	EOH	I	28	3/?	0.74	0.35	-	55,55,56,58	0
9	EDO	F	18	4/?	0.78	0.23	-	56,58,62,62	0
3	PG4	E	5	13/?	0.64	0.33	-	52,61,76,77	0
13	MOH	J	21	2/?	0.76	0.20	-	65,65,65,65	0
9	EDO	F	14	4/?	0.61	0.26	-	56,61,61,67	0
9	EDO	F	28	4/?	0.57	0.81	-	68,68,72,73	0
12	EOH	I	40	3/?	0.90	0.22	-	61,61,63,63	0
14	PGO	K	4	5/?	0.71	0.39	-	49,51,54,66	0
12	EOH	I	2	3/?	0.92	0.23	-	46,46,50,50	0
12	EOH	I	21	3/?	0.93	0.15	-	44,44,51,64	0
10	GOL	G	6	6/?	0.62	0.31	-	66,71,74,78	0
14	PGO	K	10	5/?	0.80	0.19	-	40,44,49,52	0
9	EDO	F	22	4/?	0.59	0.24	-	75,76,77,80	0
9	EDO	F	27	4/?	0.80	0.21	-	63,69,72,73	0
9	EDO	F	8	4/?	0.86	0.15	-	62,70,70,74	0
9	EDO	F	29	4/?	0.83	0.26	-	54,55,56,65	0
4	PG0	P	9	8/?	0.59	0.34	-	64,68,72,73	0
5	PEG	M	11	7/?	0.67	0.33	-	61,67,74,76	0
12	EOH	I	46	3/?	0.93	0.63	-	50,50,55,61	0
13	MOH	J	1	2/?	0.69	0.24	-	45,45,45,48	0
13	MOH	J	18	2/?	0.79	0.23	-	51,51,51,55	0
5	PEG	M	10	7/?	0.68	0.34	-	62,75,81,84	0
3	PG4	E	2	13/?	0.72	0.30	-	55,63,68,69	0
9	EDO	F	21	4/?	0.79	0.52	-	54,55,57,61	0
13	MOH	J	14	2/?	0.70	0.29	-	50,50,50,64	0
13	MOH	J	5	2/?	0.66	0.20	-	46,46,46,49	0
16	ETX	N	5	6/?	0.75	0.17	-	59,71,74,78	0
13	MOH	J	16	2/?	0.62	0.24	-	56,56,56,58	0
10	GOL	G	1	6/?	0.40	0.53	-	59,64,71,72	0
9	EDO	F	31	4/?	0.65	0.27	-	70,70,75,77	0
12	EOH	I	22	3/?	0.78	0.26	-	49,49,53,53	0
13	MOH	J	15	2/?	0.90	0.28	-	45,45,45,48	0
16	ETX	N	8	6/?	0.57	0.46	-	61,64,70,73	0
12	EOH	I	37	3/?	0.82	0.19	-	48,48,49,57	0
3	PG4	E	1	13/?	0.81	0.16	-	54,58,63,66	0
12	EOH	I	12	3/?	0.81	0.32	-	53,53,56,58	0
15	PGE	L	1	10/?	0.75	0.29	-	54,65,76,79	0
12	EOH	I	34	3/?	0.67	0.46	-	68,68,70,74	0

Continued on next page...

PRELIMINARY VALIDATION REPORT

Continued from previous page...

Mol	Type	Chain	Res	Atoms	RSCC	RSR	LLDF	B-factors(Å^2)	Q<0.9
9	EDO	F	17	4/?	0.78	0.19	-	58,58,59,60	0
9	EDO	F	11	4/?	0.77	0.21	-	67,70,71,73	0
12	EOH	I	32	3/?	0.85	0.24	-	49,49,58,59	0
13	MOH	J	12	2/?	0.93	0.13	-	54,54,54,57	0
16	ETX	N	1	6/?	0.77	0.18	-	60,64,67,70	0
12	EOH	I	39	3/?	0.67	0.29	-	64,64,70,71	0
12	EOH	I	47	3/?	0.68	0.32	-	60,60,62,65	0
12	EOH	I	8	3/?	0.51	0.26	-	48,48,62,67	0
9	EDO	F	30	4/?	0.81	0.15	-	47,51,52,56	0
13	MOH	J	22	2/?	0.61	0.31	-	62,62,62,70	0
12	EOH	I	27	3/?	0.84	0.33	-	55,55,59,68	0
13	MOH	J	8	2/?	0.87	0.13	-	51,51,51,56	0
16	ETX	N	6	6/?	0.77	0.31	-	55,62,69,73	0
11	PDO	H	4	5/?	0.75	0.25	-	60,64,70,71	0
10	GOL	G	2	6/?	0.68	0.35	-	56,58,65,65	0
12	EOH	I	7	3/?	0.86	0.20	-	55,55,55,59	0
12	EOH	I	41	3/?	0.87	0.31	-	50,50,51,61	0
12	EOH	I	14	3/?	0.75	0.26	-	58,58,61,64	0
5	PEG	M	9	7/?	0.75	0.21	-	68,73,78,81	0

6.5 Other polymers ⓘ

There are no such residues in this entry.

PRELIMINARY VALIDATION REPORT
